# Hospital mortality, withdrawal of life-sustaining therapy decisions and early secondary brain insults for critically ill traumatic brain injury patients in England, Wales and Northern Ireland (2009–2024): an observational cohort study

**DOI:** 10.1016/j.lanepe.2025.101538

**Published:** 2025-11-20

**Authors:** Xavier Chapalain, Olivier Huet, Kathryn M. Rowan, Paul R. Mouncey, Olivier Langeron, David K. Menon, David A. Harrison

**Affiliations:** a Clinical Trials Unit, Intensive Care National Audit & Research Centre, Napier House, 24 High Holborn, WC1V 6AZ, London, United Kingdom; b Department of Anaesthesiology and Critical Care, Brest University Hospital, Boulevard Tanguy Prigent, 29609 Brest Cedex, France; c Division of Anaesthesia, University of Cambridge, Addenbrooke's Hospital, Cambridge, CB2 2QQ, United Kingdom

**Keywords:** Traumatic brain injury, Cohort study, Critical care outcomes, Withdrawal life sustaining therapy, Secondary brain insults

## Abstract

**Background:**

Recent epidemiological studies reported conflicting results regarding mortality trends for traumatic brain injury (TBI) patients. Mortality trends for the critically ill TBI population, and their drivers of changes, remains understudied. Particularly, withdrawal of life-sustaining therapy (WLST) decisions were rarely evaluated concurrently. In this study, we aimed to describe hospital mortality and WLST trends over the past 15 years in England, Wales and Northern Ireland for TBI patients admitted to an intensive care unit (ICU).

**Methods:**

Observational cohort study, involving 235 adult ICUs participating in the Intensive Care National Audit & Research Centre (ICNARC) Case Mix Programme (CMP). From April 1, 2009 to March 31, 2024, all TBI patients were included. Comparator cohorts consisted of patients with trauma, sepsis, and vascular brain injury recorded in the CMP. The primary outcome was hospital mortality. The secondary outcome was the incidence of WLST decisions. We also examined the proportion of patients experiencing predefined early secondary brain insults.

**Findings:**

Of the 2,324,961 ICU admissions, we identified 45,684 unique TBI patients. Over the study period, hospital mortality for TBI patients increased from 25.6% (1021/3988) to 35.0% (1306/3727). The proportion of WLST decisions rose from 7.5% (301/4024) to 19.7% (759/3850). After adjustment for main confounders, multivariable analyses confirmed these trends. No similar trends were observed among the comparator cohorts. TBI patients were exposed to hypotension, hypocapnia, hypercapnia and hyperglycaemia in 49.8% (22,559/45,298), 29.9% (12,356/41,262), 33.6% (13,869/41,262) and 29.2% (12,127/41,505) of cases, respectively. Half of patients (50.3%, 20,747/41,265) were exposed to hypoxaemia, and this proportion increased markedly from 36.9% (1359/3684) to 61.2% (2186/3572) over time.

**Interpretation:**

For critically ill TBI patients, hospital mortality and WLST decisions rates increased over time. These findings raise important questions regarding the processes and ethical frameworks underpinning WLST decisions.

**Funding:**

10.13039/100014013UKRI, NIHR, UK Ministry of Defence, Alzheimer’s Research UK, French Society of Anaesthesiology and Critical Care, 10.13039/501100022278Gueules Cassées Foundation, INNOVEO donation fund.

**Research in context**

**Evidence before this study**

Traumatic brain injury (TBI) represents a substantial part of the global injury burden and is a leading cause of death and disability. Recent epidemiological reports indicate a growing proportion of older patients are admitted to hospital for TBI. This demographic shift could drive an increase in mortality, although advances in trauma care might counterbalance this trend. We searched PubMed on September 30, 2024, for publications in English of observational studies describing trends in mortality and care for TBI patients using the terms ‘TBI’ [All fields] OR ‘Trauma brain injury’ [All fields] AND ‘cohort’ [All fields] OR ‘cross-sectional’ [All fields] OR ‘longitudinal’ [All fields] OR ‘survey’ [All fields] ‘brain injuries’ [MeSH terms] AND ‘cohort analyses’ [MeSH terms]. We restricted our search to the articles published from January 1, 2010 to September 30, 2024. Among the 5144 studies identified, only 22 studies were retained according to the following criteria: sample size (>500 patients), long timeframe (at least five years) and well-documented database. These studies reported conflicting results depending on geographical location, setting and type of patients included. Only two studies reported mortality for critically ill patients. Crucially, no recent studies had concurrently evaluated trends in decisions to withdrawal life-sustaining therapy (WLST) or in exposure to secondary brain insults.

**Added value of this study**

In this national, multicentre, observational cohort study from the Intensive Care National Audit & Research Centre (ICNARC) Case Mix Programme (CMP), we analysed data from 45,684 critically ill patients with TBI admitted to 235 ICUs in England, Wales, and Northern Ireland from April 1, 2009 to March 31, 2024. We found that crude hospital mortality increased from 25.6% in 2009–2010 to 35.0% in 2023–2024. Concurrently, the proportion of patients for whom a WLST decision was made increased from 7.5% to 19.7%. These trends remained after adjustment for measured prognostic factors. We did not observe similar trends for other critically ill populations. Interestingly, 52.3% of WLST decisions were made during the first 3 days of ICU admission and the proportion of all hospital deaths preceded by a WLST decision grew from 30.7% to 57.8%. A substantial proportion of TBI patients were exposed to early secondary brain insults. In particular, patient exposure to early hypoxaemia increased from 36.9% in 2009–2010 to 61.2% in 2023–2024. To our knowledge, this is the largest study reported concurrent trends in hospital mortality, WLST decisions, and early secondary brain insults for the critically ill TBI population.

**Implications of all the available evidence**

Our findings reveal that for critically ill TBI patients in England, Wales, and Northern Ireland, both hospital mortality and WLST decisions have increased over the past 15 years. The substantial and growing contribution of WLST decisions to overall hospital mortality highlights an urgent need to understand factors driving WLST decisions. Moreover, a persistent exposure to secondary brain insults and the rising exposure to hypoxaemia indicates a potential target for quality improvement initiatives. Further research is needed to explore the decision-making processes for WLST in daily practice and to reinforce optimal management of fundamental physiological parameters to prevent early secondary brain insults for TBI patients.

## Introduction

Traumatic brain injury (TBI) constitutes a significant proportion of the global injury burden, affecting more than 28 million individuals annually.^1^ Among major trauma patients, TBI remains the leading cause of disability-adjusted life years lost in individuals aged from 10 to 49 years.^2^ Globally, recent data indicate a rise in TBI-related hospital admissions, with an increasing proportion of older patients.^3^, ^4^, ^5^, ^6^ Conversely, advances in trauma systems, pre-hospital and early in-hospital management may have improved outcomes for TBI patients, potentially counterbalancing the negative impact of ageing demographics.^7^^,^^8^

European and North American registries report conflicting trends in mortality rates, varying according to time-period, geographical location and patients’ characteristics.^3^^,^^8^, ^9^, ^10^, ^11^ Many report are based on large government registries, encompassing all TBI patients regardless of severity.^3^, ^4^, ^5^, ^6^^,^^10^ However, only two relatively small recent studies have evaluated trends in hospital mortality specifically in adult critically ill TBI patients or examined the drivers of these trends.^9^^,^^12^ The largest cohort study was conducted by the Finnish Intensive Care Consortium and included 7044 patients across five university hospitals.^12^ This study found a 10% absolute reduction in one-year mortality between 2003 and 2013.^12^ Moreover, recent research has highlighted a high, and variable, prevalence of withdrawal of life-sustaining therapy (WLST) in TBI patients, ranging from 20% to 85% depending on the study and intensive care unit (ICU).^13^, ^14^, ^15^, ^16^ Importantly, the majority of deaths for critically ill TBI patients occur following WLST.^15^^,^^16^ Therefore, evaluating mortality without considering for WLST decisions risks misinterpretation of outcomes for the TBI population in ICU.^17^^,^^18^ To our knowledge, no large-scale study has recently explored concurrent trends in both hospital mortality and WLST decisions among adult critically ill TBI patients.

The Intensive Care National Audit & Research Centre (ICNARC) Case Mix Programme (CMP) provides a valuable resource of clinical data on adult ICU patients.^19^ The primary objective of this study was to examine trends in hospital mortality over the past 15 years for adult critically ill TBI patients. The secondary objective was to explore trends in WLST decision-making. Our final exploratory objective was to describe the frequency of potential early secondary brain insult during the first 24 h following ICU admission.

## Methods

### Study design

This was an observational cohort study using routinely collected data from the ICNARC CMP, the national clinical audit of adult ICU admissions. The CMP database collects information on patient characteristics, care and outcomes, with coverage of all adult general critical care (intensive and intensive/high dependency care) units across England, Wales and Northern Ireland, and a high proportion of specialist units, including dedicated neuroscience ICUs. CMP data collection is authorised under Section 251, of the NHS Act 2006, which permits the use of patient-identifiable data without individual consent. This report consisted of a health service evaluation through the ICNARC national CMP and no ethical approval was needed. This study was reported in accordance with the STROBE (Strengthening the Reporting of Observational Studies in Epidemiology) guidelines.^20^

### Patients

All patients admitted to ICUs participating in the CMP between April 1, 2009 and March 31, 2024 were considered for inclusion. TBI patients were identified using the ICNARC Coding Method, based on codes in the primary and secondary reasons for ICU admission fields. The following codes were used to identify TBI patients: ‘primary brain injury’, ‘traumatic subdural haemorrhage’, ‘traumatic subarachnoid haemorrhage’, ‘extradural haemorrhage’, ‘focal brain injury’ and ‘non accidental injury to brain’. These patients formed the principal cohort for the main analysis.

From the same selected CMP cohort, three comparator cohorts were identified: trauma patients (excluding TBI, vascular brain injury or sepsis), sepsis patients (excluding TBI, vascular brain injury or trauma) and, subsequently, vascular brain injury patients (excluding TBI, trauma, or sepsis). The remaining wider ICU population (patients not included in the principal or comparator cohorts) was retained for broader comparative analyses. Patients meeting criteria for more than one comparator cohort (among trauma, sepsis and vascular brain injury) were included in the wider ICU population only. Details on ICNARC Coding Method codes are provided in the Supplementary Appendix (pp 3 and 4).

In all cohorts, to ensure independence of observations, for patients with more than one ICU admission, only the first ICU admission in any participating ICU was included.

### Outcomes and prognostic factors

The primary outcome measure was hospital mortality measured as ultimate status (alive/dead) at discharge from acute hospital. The following patient-level prognostic factors were selected, *a priori*, based on their known association with mortality following TBI: socio-demographic factors (age, sex and deprivation), surgical procedure before ICU admission (yes/no), neurological examination in the first 24 h in ICU (pupillary reactivity and motor component of Glasgow Coma Scale) and five physiological factors measured in the first 24 h in ICU (systolic blood pressure, PaO_2_, PaCO_2_, glucose level and haemoglobin). Type of ICU (general ICU, general ICU with neurosurgery and specialist neuroscience ICU) was also, *a priori*, included as a system-level factor.

The secondary outcome measure was WLST decision (yes/no), defined as the withdrawal of all clinically indicated treatments, other than comfort measures, on the grounds of lack of benefit to the patient. The same factors were selected, *a priori*, except two patient-level factors (sex and PaCO_2_) which were excluded and the level of dependence at home prior to hospital admission which was added to the analysis.

The rationale for selecting all prognostic factors and their definitions is detailed in the Supplementary Appendix (pp 5 and 6).

As an exploratory analysis, we also described the proportion of patients exposed to early secondary brain insults within the first 24 h of ICU admission. These were pre-defined as follows: hypotension (lowest systolic arterial blood pressure <100 mmHg), hypoxaemia (lowest PaO_2_ < 80 mmHg), hyperoxia (lowest PaO_2_ > 100 mmHg), hyponatremia (lowest sodium level <135 mmol/l), hypernatraemia (highest sodium level >145 mmol/l), hypothermia (lowest temperature <36 °C), hyperthermia (lowest temperature >38 °C), hypoglycaemia (lowest glucose level <4.5 mmol/l), hyperglycaemia (highest glucose level >10 mmol/l), hypocapnia (PaCO_2_ < 35 mmHg), hypercapnia (PaCO_2_ > 45 mmHg), alkalosis (pH level, associated with lowest PaO_2_ > 7.45), acidosis (lowest pH level <7.35), anaemia (lowest haemoglobin level <7 g/dl) and thrombocytopaenia (lowest platelet count <75 G/l).

### Data collection

Data for consecutive patients admitted to ICUs are prospectively captured or collected by trained local data collectors and uploaded onto a digital platform. All patients included in the CMP are followed from hospital admission to final discharge from acute hospital, including after transfer to another hospital. In the CMP, the following data are routinely collected: socio-demographic characteristics (including ethnic origin), residence prior to hospital admission, severe comorbidities, date for hospital/ICU admission, route prior to ICU admission, type of ICU, lowest/highest physiological parameters during the first 24 h in ICU, worst neurological examination during the first 24 h in ICU, daily advanced/basic organ support received in ICU, date of ICU/hospital discharge, WLST decision during ICU stay and ultimate status (alive/dead) at hospital discharge. Data quality is continuously assessed locally and curated centrally at ICNARC prior to integration into the final database. Further details regarding data elements collected and data validation have been reported previously.^19^ Throughout the study period, definitions of the CMP variables used in the analysis were unchanged, except glucose levels which were introduced at the end of year 2009. Two severity scores were derived from the CMP: the APACHE II (Acute Physiologic and Chronic Health Evaluation) and the core IMPACT (International Mission for Prognosis and Analysis of Clinical Trials) score.^21^^,^^22^ APACHE II is routinely calculated on the CMP according to age, comorbidities and physiological data recorded during the first 24 h.^22^ The core IMPACT score was used to estimate probabilities of death and poor neurological outcome.^21^ It was calculated using CMP–recorded values for age, pupillary reactivity, and the motor component of Glasgow Coma Scale (GCS).^21^

### Statistical analysis

All continuous variables were summarised as means and standard deviations (SD). Medians and interquartile ranges (IQR) were also reported for age. Categorical variables were described as counts and percentages.

Trends in hospital mortality and WLST decisions were assessed in the overall study population and in matched cohorts. To compare TBI patients with other ICU cohorts, we used a matching procedure combining exact matching for two categorical variables (type of hospital and year of ICU admission) and nearest-neighbour matching for two continuous variables (age and APACHE II score), without replacement.

Generalised linear mixed models were used to assess the association between ICU admission year and both primary (hospital mortality) and secondary (WLST decision) outcomes. The year of ICU admission was the main independent variable, with the first study period (2009–2010) serving as the reference. Other independent covariates were rigorously selected according to a process described in the Supplementary Material (Appendix pp 5 and 6).

Univariable models were initially fitted for each covariate, and results were expressed as unadjusted odds ratios (OR) with 95% confidence intervals (CI). To account for potential non-linear relationships between continuous variables and outcomes, we applied restricted cubic spline functions using three, four, and five knots. Model fit was compared using the Akaike Information Criterion (AIC) and Bayesian Information Criterion (BIC), using the three-knot model as the reference. Final spline specifications were chosen based on model fit, clinical plausibility, and consistency with existing literature.

Multivariable models included all selected covariates, regardless of their significance in univariable analysis. One random effect for admission hospital was included to account for clustering effect by hospital. All other categorical variables were introduced in the model as fixed-effect variables. Multivariable analyses were reported as adjusted odds ratios (aOR) with their 95% CI.

Primary and secondary outcomes were analysed using complete-case data. A sensitivity analysis was conducted using a dataset with missing values imputed.

As a post hoc analysis, we evaluated trends in both outcomes across subgroups defined by age, ICU type, and predicted probability of poor neurological outcome at 6 months (based on the core IMPACT model).^21^ For these trend analyses, the same generalised linear mixed models were applied in each sub-group. All details about statistical analysis are provided in the Supplementary Appendix (pp 9 and 10).

All statistical analyses were performed with R statistical software (version 4.4.1).

### Role of the funding source

These funders had no role in conceiving the study design or its conduct, and had no responsibility in writing or publishing this report.

## Results

### Study setting and participants

**Fig. 1:**
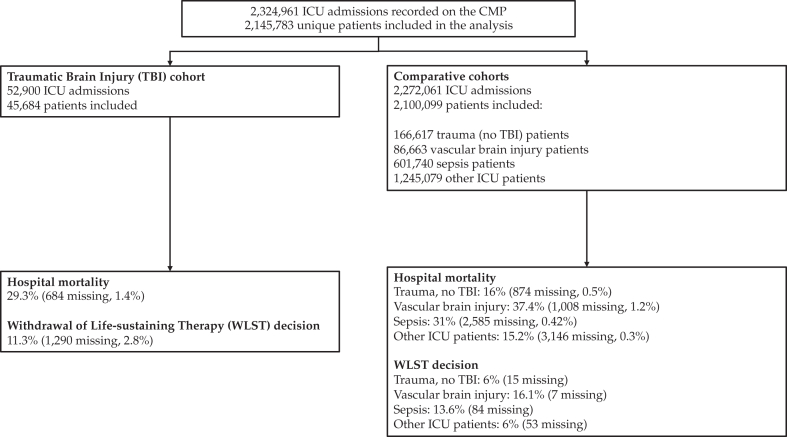
Flow diagram.

Between April 1, 2009, and March 31, 2024, a total of 2,324,961 ICU admissions were recorded in the CMP database. Of these, 52,900 admissions were for TBI as a primary or secondary diagnosis. We excluded 7216 admissions that were readmissions for TBI (Fig. 1). The final cohort for analysis comprised 45,684 patients with TBI from 235 participating ICUs: 199 general ICUs without neurosurgery, 26 general ICUs with neurosurgery, and 10 specialist neuroscience ICUs (Fig. 1, Appendix p 11). In addition, we identified 166,617 trauma patients, 601,740 sepsis patients, 86,663 vascular brain injury patients, and 1,245,079 other ICU patients (Fig. 1).

TBI patients were predominantly male (73.6%, 33,628/45,684) with a mean (SD) age of 50.1 (20.2) years (Table 1). They were admitted to general ICUs without neurosurgery (37.6%, 17,178/45,684), general ICUs with neurosurgery (41.9%, 19,120/45,684) and specialist neuroscience ICUs (20.5%, 9386/45,684) (Table 1). The predicted probabilities of death and poor neurological outcome calculated with the core IMPACT score increased over time, primarily driven by a rise in mean patient age from 44.8 (20.0) years in 2009–2010 to 52.6 (19.8) years in 2023–2024 (Table 1). Other characteristics of the TBI patients and patient characteristics for comparator cohorts are reported in the Supplementary Appendix (pp 12–16).

**Table 1:** Patient- and centre-level characteristics for TBI patients.

Variables	Overall	April 2009–2010	2011–2012	2013–2014	2015–2016	2017–2018	2019–2020	2021–2022	2023–March 2024
	N = 45,684	N = 4072	N = 5011	N = 5386	N = 6919	N = 7390	N = 6730	N = 6318	N = 3858
**Patient-level factors**									
Age, years									
Mean (SD)	50.1 (20.2)	44.8 (20.0)	46.9 (20.4)	50.0 (20.5)	50.3 (20.6)	51.2 (20.0)	51.7 (19.8)	51.3 (19.6)	52.6 (19.8)
Median (IQR)	51 (33–67)	44 (27–60)	46 (29–63)	50 (32–67)	51 (32–68)	52 (34–68)	53 (35–68)	53 (35–68)	55 (36–69)
Sex—no. (%)									
Male	33,628 (73.6)	3079 (75.6)	3813 (76.1)	3975 (73.8)	5088 (73.5)	5364 (72.6)	4880 (72.5)	4624 (73.2)	2805 (72.7)
Female	12,055 (26.4)	993 (24.4)	1198 (23.9)	1411 (26.2)	1831 (26.5)	2026 (27.4)	1850 (27.5)	1693 (26.8)	1053 (27.3)
Dependence category^a^—no. (%)									
Without assistance	40,645 (91.0)	3692 (93.6)	4428 (91.0)	4753 (90.7)	6101 (90.2)	6596 (90.8)	6111 (91.9)	5633 (91.3)	3331 (89.0)
Some assistance	3768 (8.4)	240 (6.1)	420 (8.6)	467 (8.9)	632 (9.3)	628 (8.6)	520 (7.8)	497 (8.1)	364 (9.7)
Total assistance	228 (0.5)	14 (0.4)	19 (0.4)	21 (0.4)	31 (0.5)	43 (0.6)	16 (0.2)	38 (0.6)	46 (1.2)
Residence prior to ICU—no. (%)									
Home	44,326 (97.0)	3921 (96.3)	4831 (96.4)	5206 (96.7)	6711 (97.0)	7170 (97.0)	6564 (97.5)	6165 (97.6)	3758 (97.4)
Other location	1355 (3.0)	151 (3.7)	180 (3.6)	180 (3.3)	208 (3.0)	218 (3.0)	166 (2.5)	153 (2.4)	99 (2.6)
Deprivation index^b^—no. (%)									
1 (least deprived)	6636 (14.5)	586 (14.4)	751 (15.0)	759 (14.1)	998 (14.4)	1025 (13.9)	976 (14.5)	952 (15.1)	589 (15.3)
2	7572 (16.6)	670 (16.5)	818 (16.3)	861 (16.0)	1148 (16.6)	1214 (16.4)	1127 (16.7)	1070 (16.9)	664 (17.2)
3	8487 (18.6)	732 (18.0)	939 (18.7)	973 (18.1)	1310 (18.9)	1354 (18.3)	1294 (19.2)	1175 (18.6)	710 (18.4)
4	9660 (21.1)	908 (22.3)	1062 (21.2)	1177 (21.9)	1411 (20.4)	1593 (21.6)	1399 (20.8)	1323 (20.9)	787 (20.4)
5 (most deprived)	11,710 (25.6)	1052 (25.8)	1259 (25.1)	1425 (26.5)	1819 (26.3)	1928 (26.1)	1704 (25.3)	1590 (25.2)	933 (24.2)
Severe comorbidities—no. (%)									
Cardiovascular disease^c^	123 (0.3)	11 (0.3)	10 (0.2)	10 (0.2)	26 (0.4)	24 (0.3)	24 (0.4)	13 (0.2)	5 (0.1)
End-stage kidney disease^c^	172 (0.4)	6 (0.1)	10 (0.2)	28 (0.5)	24 (0.3)	32 (0.4)	22 (0.3)	29 (0.5)	21 (0.5)
Cirrhosis	349 (0.8)	19 (0.5)	36 (0.7)	33 (0.6)	33 (0.5)	30 (0.4)	35 (0.5)	75 (1.2)	88 (2.3)
Neoplasm with metastasis	205 (0.4)	8 (0.2)	20 (0.4)	17 (0.3)	31 (0.4)	33 (0.4)	31 (0.5)	36 (0.6)	29 (0.8)
Lymphoma	82 (0.2)	3 (0.1)	6 (0.1)	9 (0.2)	12 (0.2)	13 (0.2)	14 (0.2)	15 (0.2)	10 (0.3)
Long-term steroid medication	143 (0.3)	10 (0.2)	10 (0.2)	11 (0.2)	23 (0.3)	22 (0.3)	18 (0.3)	25 (0.4)	24 (0.6)
TBI category—no. (%)									
Subdural haemorrhage	16,859 (37.2)	1240 (30.7)	1772 (35.6)	1960 (36.7)	2606 (38.0)	2788 (38.0)	2490 (37.3)	2486 (39.6)	1517 (39.7)
Diffuse brain injury	10,170 (22.4)	1377 (34.1)	1446 (29)	1308 (24.5)	1471 (21.4)	1439 (19.6)	1198 (18.0)	1165 (18.5)	766 (20.0)
Extradural haemorrhage	3783 (8.3)	406 (10.1)	425 (8.5)	480 (9.0)	622 (9.1)	610 (8.3)	561 (8.4)	450 (7.2)	229 (6.0)
Other trauma brain injuries^d^	14,515 (32.0)	1010 (25.0)	1335 (26.8)	1586 (29.7)	2167 (31.6)	2500 (34.1)	2420 (34.1)	2184 (34.7)	1313 (34.3)
Pupillary reactivity^f^—no. (%)									
Both reacting	34,330 (80.4)	2068 (80.8)	3738 (80.9)	4115 (80.0)	5535 (82.2)	5703 (79.8)	5261 (80.2)	4906 (79.3)	3004 (79.4)
One reactive	1653 (3.9)	108 (4.2)	196 (4.2)	223 (4.3)	227 (3.4)	299 (4.2)	242 (3.7)	220 (3.6)	138 (3.6)
Both unreactive	6742 (15.8)	383 (15.0)	688 (14.9)	807 (15.7)	968 (14.4)	1144 (16)	1054 (16.1)	1057 (17.1)	641 (16.9)
GCS category^g^—no. (%)									
13–15	9558 (41.2)	771 (38.1)	1050 (41.0)	1184 (41.4)	1610 (44.4)	1633 (41.4)	1409 (40.3)	1171 (40.8)	730 (40.6)
9–12	4852 (20.9)	419 (20.7)	498 (19.5)	613 (21.4)	771 (21.2)	824 (20.9)	757 (21.7)	593 (20.6)	377 (21.0)
3–8	8770 (37.8)	832 (41.1)	1011 (39.5)	1066 (37.2)	1249 (34.4)	1483 (37.6)	1330 (38.0)	1109 (38.6)	690 (38.4)
GCS motor component^g^—no. (%)									
Obey command or localise pain	15,418 (34.5)	1223 (31.0)	1689 (34.7)	1892 (36.1)	2523 (37.3)	2644 (36.5)	2341 (35.5)	1918 (30.9)	1188 (31.3)
Withdrawal from pain	1522 (3.4)	180 (4.6)	187 (3.8)	215 (4.1)	251 (3.7)	228 (3.2)	219 (3.3)	153 (2.5)	89 (2.3)
Flexion to pain	593 (1.3)	69 (1.8)	86 (1.8)	75 (1.4)	89 (1.3)	91 (1.3)	70 (1.1)	62 (1.0)	51 (1.3)
Extension to pain	659 (1.5)	73 (1.9)	81 (1.7)	98 (1.9)	77 (1.1)	109 (1.5)	100 (1.5)	64 (1.0)	57 (1.5)
No movement	4988 (11.2)	477 (12.1)	516 (10.6)	583 (11.1)	690 (10.2)	868 (12.0)	766 (11.6)	676 (10.9)	412 (10.9)
GCS, Untestable^g^—no. (%)	21,452 (48.1)	1918 (48.7)	2311 (47.5)	2378 (45.4)	3129 (46.3)	3294 (45.5)	3094 (46.9)	3328 (53.7)	2000 (52.7)
Core IMPACT score^e^									
Predicted mortality, %—mean (SD)	34.8 (20.8)	32.1 (20.1)	32.4 (20.3)	34.6 (20.6)	33.9 (20.5)	35.4 (21.1)	35.7 (21.0)	35.9 (20.7)	36.8 (20.8)
Predicted probability of poor neurological outcome, %—mean (SD)	52.0 (22.6)	48.8 (22.5)	49.2 (22.8)	51.6 (22.7)	50.9 (22.6)	52.5 (22.7)	52.9 (22.6)	53.6 (22.4)	54.6 (22.2)
Surgical procedure—no. (%)									
No	34,565 (75.7)	3137 (77.0)	3828 (76.4)	3997 (74.2)	5213 (75.3)	5578 (75.5)	5125 (76.2)	4754 (75.2)	2933 (76.0)
Yes	11,119 (24.3)	935 (23.0)	1183 (23.6)	1389 (25.8)	1706 (24.7)	1812 (24.5)	1605 (23.8)	1564 (24.8)	925 (24.0)
Emergency surgery	10,516 (94.6)	896 (95.8)	1116 (94.3)	1298 (93.4)	1598 (93.7)	1709 (94.3)	1515 (94.4)	1504 (96.2)	880 (95.1)
Mechanical ventilation^h^—no. (%)	31,174 (68.2)	2775 (68.1)	3386 (67.6)	3651 (67.8)	4717 (68.2)	5046 (68.3)	4642 (69.0)	4325 (68.5)	2632 (68.2)
**Centre-level factors**									
ICU type—no. (%)									
General ICU without neurosurgery	17,178 (37.6)	1549 (38.0)	1870 (37.3)	1685 (31.3)	2083 (30.1)	2082 (28.2)	1907 (28.3)	1772 (28.0)	1114 (28.9)
Transfer to neuroscience ICU^i^	1472 (10.5)	254 (16.4)	274 (14.7)	189 (11.2)	177 (8.5)	172 (8.3)	161 (8.4)	136 (7.7)	109 (9.8)
General ICU with neurosurgery	19,120 (41.9)	1834 (45.0)	2349 (46.9)	2979 (55.3)	3320 (48.0)	3632 (49.1)	3209 (47.7)	3064 (48.5)	1849 (47.9)
Specialist neuroscience ICU	9386 (20.5)	689 (16.9)	792 (15.8)	722 (13.4)	1516 (21.9)	1676 (22.7)	1614 (24.0)	1482 (23.5)	895 (23.2)
Type of hospital—no. (%)									
Non-university	7474 (16.4)	1184 (29.1)	1170 (23.3)	952 (17.7)	1041 (15.0)	960 (13.0)	872 (13.0)	745 (11.8)	550 (14.3)
University	29,855 (65.4)	2186 (53.7)	3034 (60.5)	3449 (64.0)	4568 (66)	5005 (67.7)	4597 (68.3)	4438 (70.2)	2578 (66.8)
University affiliated	8355 (18.3)	702 (17.2)	807 (16.1)	985 (18.3)	1310 (18.9)	1425 (19.3)	1261 (18.7)	1135 (18.0)	730 (18.9)

*Abbreviations:* CI, Confidence Interval; GCS, Glasgow Coma Scale; ICU, Intensive Care Unit; IMPACT, International Mission for Prognosis and Analysis of Clinical Trials; IQR, Interquartile Range; SD, Standard Deviation.

a The level of dependence prior to ICU admission.

b Derived from the patient's usual residential postcode according to the Index of Multiple Deprivation 2010 for England, Welsh Index of Multiple Deprivation 2008 or Northern Ireland Multiple Deprivation Measure 2010.

c Severe cardiovascular comorbidities denote patients with symptoms at rest. End-stage renal disease corresponds to patients needed dialysis.

d It includes traumatic subarachnoid haemorrhage, traumatic focal brain injury and unspecified brain injury.

e Core IMPACT score was calculated from the original prognostic model from Steyerberg et al. including three parameters: age, pupillary reactivity and GCS motor component.^21^ This prognostic model calculates the predicted probability (in percentage) of mortality and poor neurological outcomes (mortality and severe neurological disability) at 6 months.

f The most abnormal pupil reactivity, for left and right eye, assessed and recorded as a pair in the first 24 h in the ICU.

g The motor component of the lowest GCS assessed during the first 24 h in ICU. For sedated patients, GCS was not rated and was considered ‘untestable’.

h Denote number/proportion of patients under mechanical ventilation during the first 24 h following ICU admission.

i Number and percentage of patients initially admitted to a general ICU and subsequently transferred to a specialist neuroscience centre.

### Primary outcome

**Fig. 2:**
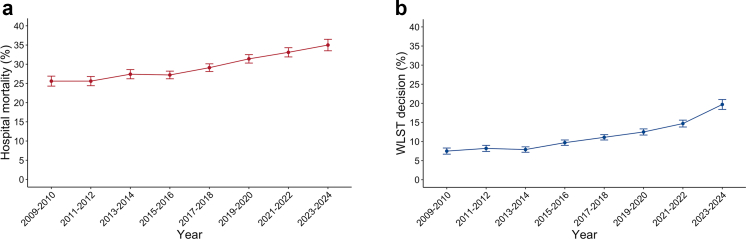
Percentage and 95% confidence interval for hospital mortality and WLST decision for TBI from April 2009 to March 2024. **a:** Hospital mortality. **b:** WLST decision.

**Fig. 3:**
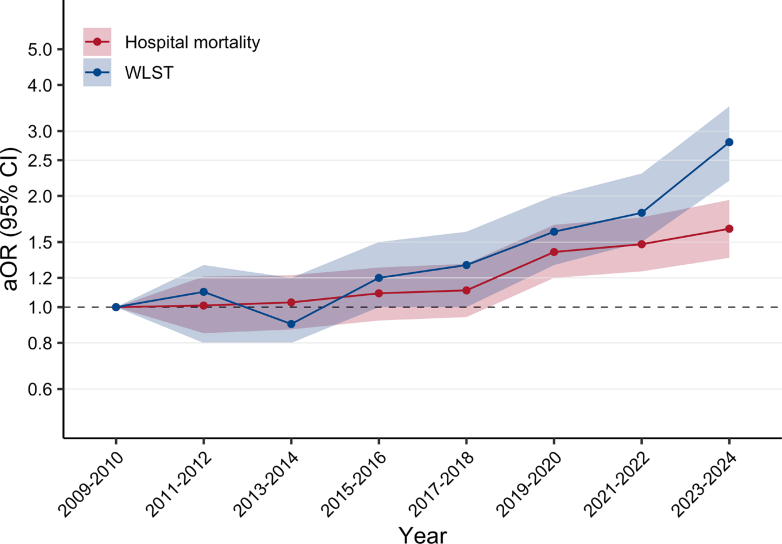
Adjusted Odds Ratio (aOR) for hospital mortality and WLST decision by year of admission for TBI patients. Dots represent the aOR by year, and shading indicates 95% CI. Adjusted OR and 95% CI are represented on a logarithmic scale. The reference period for both models is the first study period (April 2009–2010). For hospital mortality (red dots), aOR are adjusted for age, sex, deprivation index, type of ICU, surgery, pupillary reactivity, GCS motor component, lowest systolic arterial pressure, lowest PaO_2_, PaCO_2_, lowest glucose, highest glucose, lowest haemoglobin and admission hospital (random effect). Model performance is detailed in Fig. S2 and full results for all covariates are reported in Table S7 and Fig. S1. For WLST (blue dots), aOR are adjusted for age, dependence at home, type of ICU, surgery, pupil reactivity, GCS motor component, deprivation index, lowest systolic arterial pressure, lowest PaO_2_, lowest glucose level, highest glucose level, lowest haemoglobin and admission hospital (random effect). Model performance is detailed in Fig. S10 and full results for all covariates are reported in Table S9 and Fig. S9.

**Fig. 4:**
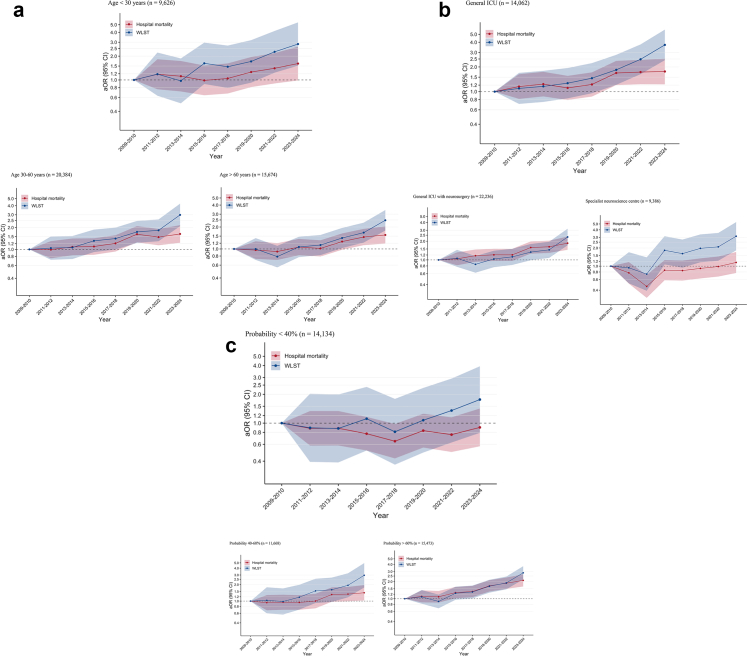
Subgroup analysis. **a:** Age. **b:** Type of ICU. **c:** Probability of poor neurological outcome at 6 months according to core IMPACT score. In each subgroup analysis, dots represent the aOR by year, and shading indicates 95% CI. Adjusted OR and 95% CI are represented on a logarithmic scale. The reference period for both models is the first study period (April 2009–2010). For hospital mortality (red dots), aOR are adjusted for age, sex, deprivation index, type of ICU (except for subgroup analysis corresponding to the type of ICU), surgery, pupillary reactivity, GCS motor component, lowest systolic arterial pressure, lowest PaO_2_, PaCO_2_, lowest glucose, highest glucose, lowest haemoglobin and admission hospital (random effect). For WLST (blue dots), aOR are adjusted for age, dependence at home, type of ICU (except for subgroup analysis corresponding to the type of ICU), surgery, pupil reactivity, GCS motor component, deprivation index, lowest systolic arterial pressure, lowest PaO_2_, lowest glucose level, highest glucose level, lowest haemoglobin and admission hospital (random effect).

Among TBI patients, hospital mortality progressively increased over time, from 25.6% (1021/3988) in 2009–2010 to 35.0% (1306/3727) in 2023–2024 (Table 2 and Fig. 2). In the multivariable analysis, the risk of hospital mortality increased steadily over time, independently of other covariates (Fig. 3 and Appendix p 17). Patients admitted to general ICU with neurosurgery (aOR 0.81; 95% CI, 0.69–0.95) and to specialist neuroscience ICU (aOR 0.80; 95% CI, 0.70–0.94) had a lower risk of hospital mortality compared with those admitted to general ICU without neurosurgery (Appendix p 17). Older age, abnormal neurological examination (pupillary reactivity and GCS motor) during the first 24 h of ICU admission, hypoxaemia, hypotension, hypocapnia and hyperglycaemia were associated with a higher risk of hospital mortality (Appendix pp 17 and 18). Model performance is reported in the Supplementary Appendix (p 19). Sensitivity analyses using an imputed dataset reinforced the findings of the primary outcome analysis (Appendix p 20). The trend for the risk of hospital mortality was consistent across all TBI subgroups, except for patients with a lower predicted probability of poor neurological outcome and for patients admitted to specialist neuroscience centres (Fig. 4). The trend in hospital mortality remained unchanged in a post-hoc sensitivity analysis restricted to ICUs that participated throughout the entire study period (Appendix p 24).

**Table 2:** Primary and secondary outcome measures over the study period.

Variables	Overall	April 2009–2010	2011–2012	2013–2014	2015–2016	2017–2018	2019–2020	2021–2022	2023–March 2024
	N = 45,684	N = 4072	N = 5011	N = 5386	N = 6919	N = 7390	N = 6730	N = 6318	N = 3858
**Primary outcome**									
Hospital mortality									
Number of patients	13,184	1021	1260	1461	1865	2130	2084	2057	1306
Percentage	29.3	25.6	25.6	27.4	27.2	29.1	31.4	33.1	35.0
95% CI	28.9–29.7	24.3–26.9	24.4–26.8	26.2–28.6	26.2–28.2	28.1–30.1	30.3–32.5	31.9–34.3	33.5–36.5
Day-30 mortality	12,460 (27.7)	963 (24.2)	1191 (24.2)	1365 (25.7)	1753 (25.6)	2018 (27.6)	1991 (30.0)	1941 (31.2)	1238 (33.2)
Day-90 mortality	13,000 (28.9)	1006 (25.3)	1237 (25.2)	1429 (26.9)	1829 (26.8)	2102 (28.8)	2066 (31.2)	2033 (32.7)	1298 (34.8)
Brainstem death—no. (%)	2511 (5.5)	206 (5.1)	241 (4.8)	332 (6.2)	335 (4.8)	428 (5.8)	407 (6.0)	360 (5.7)	202 (5.2)
**Secondary outcome**									
WLST decision—no. (%)									
Number of patients	5023	301	400	411	649	792	806	905	759
Percentage	11.3	7.5	8.2	7.9	9.7	11.1	12.5	14.7	19.7
95% CI	11.0–11.5	6.7–8.3	7.4–9.0	7.2–8.6	9.0–10.4	10.4–11.8	11.7–13.3	13.8–15.6	18.4–21.0
Time from ICU admission to WLST, days									
Mean (SD)	4.8 (8.0)	3.4 (4.3)	3.4 (3.9)	4.3 (5.6)	4.5 (6.9)	5 (13.1)	4.7 (5.9)	5.8 (7.7)	5.6 (7.4)
Median (IQR)	2 (1–6)	2 (1–5)	2 (1–5)	2 (1–6)	2 (1–6)	2 (1–6)	2 (1–7)	3 (1–8)	3 (1–8)
Before day 3	2624 (52.3)	171 (56.8)	233 (58.2)	219 (53.3)	365 (56.2)	430 (54.3)	418 (51.9)	437 (48.3)	351 (46.2)
From day 3 to day 7	1021 (20.3)	91 (30.2)	111 (27.8)	124 (30.2)	166 (25.6)	200 (25.3)	227 (28.2)	240 (26.5)	218 (28.7)
After day 7	1377 (27.4)	39 (13.0)	56 (14.0)	68 (16.5)	118 (18.2)	162 (20.5)	161 (20.0)	227 (25.1)	190 (25.0)
Death after a WLST decision—no. (% of deaths in hospital)	4960 (41.6)	299 (30.7)	398 (34.5)	405 (31.2)	643 (39.5)	777 (41.6)	794 (44.0)	892 (47.3)	752 (57.8)
**Other outcomes**									
ICU length of stay—mean (SD)	7.8 (9.7)	6.3 (8.5)	6.5 (8.6)	6.9 (8.6)	7.9 (10.0)	8.0 (10.4)	7.9 (9.6)	8.9 (10.2)	9.2 (10.4)
Hospital length of stay—mean (SD)	32.6 (38.3)	30.3 (38.5)	30.5 (40.9)	30.9 (38.5)	32.7 (38.7)	32.7 (38.7)	31.6 (33.7)	35.8 (39.1)	36.9 (38.1)

*Abbreviations:* CI, Confidence Interval; IQR, Interquartile Range; SD, Standard Deviation; WLST, Withdrawal Life-Sustaining Therapy.

Among trauma and sepsis patients, hospital mortality declined over time from 20.7% (4150/20,048) in 2009–2010 to 13.8% (1475/10,688) in 2023–2024 and from 37.2% (16,702/44,898) in 2009–2010 to 26.6% (13,804/51,894) in 2023–2024 respectively (Appendix pp 13 and 14 and p 21). Among patients with vascular brain injuries, hospital mortality initially decreased from 41.6% (2797/6724) in 2009–2010 to 34.6% (4459/12,887) in 2015–2016, followed by a gradual increase to 40.7% (3101/7619) in 2023–2024 (Appendix p 15 and p 21). Hospital mortality was relatively stable over time for other ICU patients (Appendix p 16 and p 21). Trends observed in matched cohorts were consistent with those in the full cohorts (Appendix pp 22 and 23). Except for sepsis patients, the COVID-19 pandemic did not appear to alter hospital mortality trends (Appendix p 25).

### Secondary outcome

Among TBI patients, the proportion of WLST decisions progressively increased over time, from 7.5% (301/4024) in 2009–2010 to 19.7% (759/3850) in 2023–2024 (Table 2 and Fig. 2). Half of all WLST decisions (52.3%, 2624/5017) occurred within the first three days of ICU admission (Table 2). Of all deaths, 41.6% (4960/11,923) occurred following a WLST decision (Table 2). This proportion increased from 30.7% (299/973) to 57.8% (752/1301) over time (Table 2). In the multivariable analysis, the risk of a WLST decision increased steadily over time, independently of other covariates (Fig. 3 and Appendix p 26). Older age and abnormal neurological examination at ICU admission (pupillary reactivity and GCS motor) were also associated with a higher risk of a WLST decision (Appendix pp 26 and 27). Model performance is reported in the Supplementary Appendix (p 28). Sensitivity analyses using imputed data reinforced the results of the secondary outcome analysis (Appendix p 29). The trend for the risk of a WLST decision was consistent across all TBI subgroups (Fig. 4). The trend in WLST remained unchanged in a post-hoc sensitivity analysis restricted to ICUs that participated throughout the entire study period (Appendix p 25).

In contrast, the proportion of WLST decisions slightly decreased in trauma and sepsis patients from 7.3% (1471/20,157) in 2009–2010 to 5.9% (638/10,824) in 2023–2024 and from 14.6% (6596/45,180) in 2009–2010 to 13.1% (6851/52,300) in 2023–2024, respectively (Appendix pp 13 and 14 and p 21). Among patients with vascular brain injury, the proportion of WLST decisions increased from 15.1% (1024/6781) in 2009–2010 to 19.8% (1553/7845) in 2023–2024 (Appendix p 15 and p 21). In other ICU patients, WLST decision rates remained stable over time (Appendix p 16 and p 21). We observed the same trends in the matched cohorts (Appendix pp 22 and 23). Following the COVID-19 pandemic, an increase in WLST decisions was observed among both TBI and vascular brain injury patients (Appendix p 25).

### Exploratory outcomes

**Fig. 5:**
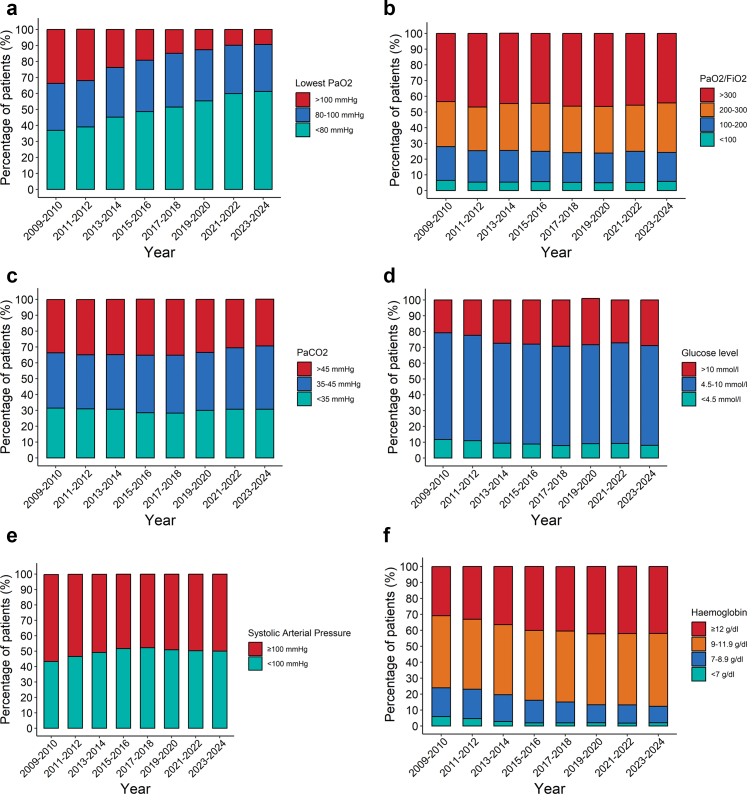
Bar charts representing percentages of early secondary brain insults recorded during the first 24 h following ICU admission. **a:** Lowest partial pressure of oxygen (PaO_2_). This bar chart represents the lowest PaO_2_ measured (in mmHg) on arterial blood gas. Overall, 41,265 measures were available (4419 were lacking). **b:** PaO_2_/FiO_2_ ratio. This bar chart represents the ratio between the lowest PaO_2_ in the first 24 h in ICU and the fraction of inspired oxygen (FiO_2_) recorded at the same time. For spontaneous breathing patients, FiO_2_ was reported according to predefined rules in the CMP data collection manual. Overall, 41,247 measures were available (4437 were lacking). **c:** Partial pressure of carbon dioxide (PaCO_2_). This bar chart represents PaCO_2_ level recorded, on arterial blood gas (in mmHg), either concomitantly with the lowest PaO_2_ or with the lowest pH. 41,262 measures were available for at least one parameter (4422 were lacking for both PaCO_2_ levels). 2088 patients (5.1%) had both hypocapnia (PaCO_2_ <35 mmHg) and hypercapnia (PaCO_2_ >45 mmHg), they are counted in both categories. **d:** Glucose level. This bar chart represents lowest and highest serum glucose levels recorded (in mmol/l). 41,505 measures were available for at least one parameter (4179 were lacking for both glucose levels). 807 patients (1.9%) had both hypoglycaemia (lowest glucose <4.5 mmol/l) and hyperglycaemia (highest glucose >10 mmol/l), they are counted in both categories. **e:** Systolic Arterial blood Pressure (SAP). This bar chart represents lowest and highest SAP recorded, irrespective of the measurement method used. 45,298 measures were available for at least one parameter (386 were lacking for both). **f:** Lowest haemoglobin level. This bar chart represents the lowest haemoglobin level measured (in g/dl). 43,343 measures were available (2341 were lacking).

Within the first 24 h, a substantial proportion of TBI patients were exposed to early secondary brain insults. These included hypoxaemia (50.3%, 20,747/41,265), hypotension (49.8%, 22,559/45,298), hypocapnia (29.9%, 12,356/41,262), hypercapnia (33.6%, 13,869/41,262) and hyperglycaemia (29.2%, 12,127/41,505) (Fig. 5 and Appendix pp 30 and 31). A smaller proportion experienced hypoglycaemia (7.0%, 2915/41,642) or anaemia (2.7%, 1153/42,703) (Fig. 5 and Appendix pp 30 and 31). Exposure to other early secondary brain insults exposure ranged from 5.3% (2292/43,245) for thrombocytopaenia to 45.9% (17,946/39,098) for acidosis and are described in the Supplementary Appendix (pp 30 and 31). The proportion of patients exposed to hypoxaemia increased over time, from 36.9% (1359/3684) in 2009–2010 to 61.2% (2186/3572) in 2023–2024 (Fig. 5, panel A). PaO_2_/FiO_2_ ratio remained relatively stable across the study period (Fig. 5, panel B). For other early secondary brain insults, no meaningful variation in exposure was observed over time (Fig. 5 and Appendix pp 30 and 31).

## Discussion

In this nationwide cohort study, we observed a consistent increase in hospital mortality among adult critically ill TBI patients, rising from 25.6% to 35.0% over the 15-year study period, independent of measured prognostic factors. Over the same period, the proportion of WLST decisions also increased, from 7.5% to 19.7%, with this trend similarly found to be independent. These trends sharply contrasted with those seen in other critically ill populations. Regarding early secondary brain insults, half of patients were exposed to hypoxaemia and hypotension, and one-third to hypocapnia, hypercapnia and hyperglycaemia during the first 24 h following ICU admission. While the incidence of most secondary brain insults was stable, the incidence of hypoxaemia notably rose from 36.9% to 61.2% over time.

We suggest the increase in hospital mortality for TBI patients can be attributed to several factors. First, our results raise important questions about the WLST decision-making process in critically ill TBI patients, especially how decisions were reached over time. At the bedside, WLST decisions are made based on predicted poor outcomes.^23^^,^^24^ Existing prognostic models have limited performance and do not provide sufficient accuracy to support high-confidence prognostication.^21^^,^^25^ Consequently, WLST decisions remain complex and are influenced not only by clinical factors, but also by patient and family preferences. Guidelines from the Neurocritical Care Society recommend maintaining life-sustaining therapy for at least 72 h before making prognostic determinations.^23^ These guidelines are reinforced by recent findings from the TRACK-TBI studies, which have shown that neurological recovery can be slow and prolonged, sometimes extending beyond two weeks, even in patients with initially unfavourable neurological assessments.^26^ In the TRACK-TBI cohort, more than one-third of patients who did not receive WLST, despite having similar baseline characteristics to those who did, achieved partial independence at six months.^24^ In our cohort, the mean time to WLST was 4.8 days, with more than 50% of decisions made before day 3. This result is consistent with other recent European and North American studies reporting similarly early WLST decision timing.^13^^,^^16^ We also observed an increase in the proportion of WLST decisions in patients with vascular brain injury, for whom similar prognostic challenges exist.^13^ Altogether, the rising hospital mortality rate for TBI patients could partly be a self-fulfilling prophecy driven by early WLST decisions. It is possible that the methods for determining individual neurological prognosis have changed over time, resulting in more withdrawals of care due to a potential overestimation of poor neurological outcomes. An alternative hypothesis is that more TBI patients with an expectedly poor prognosis were admitted to the ICU to allow for a formal prognostication period before making decisions about withdrawing care. Second, our findings suggest that a clinical trend toward lower oxygenation targets may be causing more acute cerebral injuries secondary to hypoxaemia, which in turn could contribute to an increase in hospital mortality. This may reflect a shift in ICU practice related to concerns over hyperoxia, as suggested by a simultaneous reduction in hyperoxia rates in our population. In recent clinical trials comparing conservative and liberal oxygen administration, brain injury patient subgroups included a small proportion of TBI patients.^27^, ^28^, ^29^ Their imprecise and inconsistent findings in this specific population preclude any definitive conclusion for TBI patients.^27^, ^28^, ^29^ Alternatively, the observed trend of increasing hypoxaemia could also reflect an increased incidence of acute lung injury in our population, perhaps due to thoracic trauma, though this is unlikely given the stable PaO_2_/FiO_2_ ratios observed. Regardless of the cause, hypoxaemia was associated with an increased risk of mortality in our multivariable analysis, reinforcing its potential impact on outcomes in TBI patients. Third, we cannot exclude the possibility that the severity of TBI increased over the study period. Although GCS scores upon ICU admission remained stable over time, our dataset lacks other key indicators needed for a comprehensive assessment of baseline severity. Specifically, granular pre-hospital data and CT scan findings were unavailable, preventing us from definitively refuting the hypothesis that injury severity increased. For instance, the reorganisation of UK trauma systems during this period could have led to changes in pre-hospital care, such as transport times, which may have influenced mortality.

Finally, our multivariate analysis confirms that hypoxaemia, hypotension, hypocapnia, and hyperglycaemia are significantly associated with hospital mortality. These findings corroborate a large body of observational evidence on the impact of individual secondary brain insults in TBI patients.^30^, ^31^, ^32^, ^33^, ^34^, ^35^, ^36^, ^37^ However, a key and unexpected finding was the persistently high proportion of patients exposed to early secondary brain insults, despite the widespread dissemination of international management guidelines.^38^, ^39^, ^40^ This underscores the profound difficulty of maintaining physiological stability in this population and highlights a crucial knowledge-to-practice gap. The challenge lies not just in the existence of guidelines, but in their consistent, real-time application within a complex ICU environment. This reality has significant implications for clinical experts, guideline committees, and healthcare policy makers. It signals that future efforts must extend beyond the development of evidence-based recommendations to also address the systemic challenges of implementation, such as investment in advanced monitoring technologies, decision-support tools, and continuous quality improvement programmes. More specifically, our analysis revealed a concerning trend: the proportion of patients experiencing hypoxaemia increased significantly over the study period, while exposure to other secondary brain insults remained substantial but stable. We do not believe this necessarily reflects a deterioration in the quality of care, but hypothesise several non-mutually exclusive explanations for this isolated increase. First, a shifting patient case-mix may be a key factor. Improvements in pre-hospital and immediate resuscitation may now enable the survival of patients with more severe multi-organ injuries, leading to a higher prevalence of respiratory failure in the ICU population we studied. Second, this trend could be influenced by a surveillance bias; the increased adoption of advanced tools, such as continuous brain tissue oxygen monitoring, may simply be getting better at identifying episodes of cerebral hypoxia that previously went undetected. Third, evolving clinical practice, such as the adoption of lung-protective ventilation strategies that tolerate lower oxygenation targets, may also contribute to this finding as mentioned above. Regardless of the precise cause, this specific rise in hypoxaemia is a critical observation, suggesting that respiratory management in the TBI population requires renewed attention and investigation.

This study has several strengths. First, it represents the largest published cohort of adult critically ill TBI patients to date, offering a comprehensive view of mortality trends in this high-risk population. Second, the CMP database is a rigorously curated, high-quality dataset with over 30 years of continuous data collection using standardised definitions. The study period was restricted to April 2009 onwards as a substantial proportion of neurocritical care units began participating in the CMP from this date. Third, the CMP also enabled comparison between outcomes of TBI patients and those of other ICU patients admitted during the same period, which is rarely done. Fourth, we concurrently analysed trends in both hospital mortality and WLST decisions, two intimately linked outcomes that have rarely been studied together, despite evidence showing that a large proportion of deaths in TBI patients follow a WLST decision.^14^^,^^15^ Fifth, we adjusted our analyses for multiple key variables known to influence TBI prognosis, including components of both the IMPACT and CRASH models (age, pupillary reactivity, and GCS motor response), as well as additional predictors such as PaO_2_, PaCO_2_, glucose and haemoglobin levels.^21^^,^^25^ We also accounted for socioeconomic and system-level factors.^14^ The robustness of our multivariable models reinforces the validity of our findings by minimising potential bias from patient- and system-level confounders.

This study also has some limitations. First, our findings are limited to England, Wales and Northern Ireland. While this reduces variability from different healthcare systems, it may limit generalisability. Second, ICU coverage within the CMP expanded over the study period, which may have influenced the observed mortality trends. Notably, the number of specialist neuroscience centres participating in the CMP increased over the study period, which may have possibly influenced practice patterns and outcomes. However, a sensitivity analysis restricted to patients from ICUs that participated throughout the entire study period confirmed the findings of our primary trend analysis. Third, the CMP dataset was not specifically designed for TBI research and therefore lacks key variables. We did not have data on pre-hospital care (such as the mechanism of trauma or initial neurological evaluation), detailed comorbidities, CT scan findings, or neurosurgical interventions. The absence of this information precluded the use of standard TBI severity metrics, such as the Injury Severity Score or Marshall classification. Furthermore, the dataset lacked advanced neurological parameters (e.g., intracranial pressure, brain tissue oxygenation or biomarkers) and data on functional outcomes (e.g., the Extended Glasgow Outcome Scale), which would have greatly enriched our analysis. Fourth, the data were insufficient to determine how decisions regarding WLST were made or how neurological prognosis was assessed, or to evaluate how clinical practices evolved over the study period. Specifically, information on patient wishes (e.g., do not attempt cardiopulmonary resuscitation decisions) or baseline therapeutic goals at ICU admission was not recorded, preventing an analysis of their influence on clinical decisions. Fifth, trauma care networks were reorganised in the UK during the study period. Although this reorganisation likely impacted care delivery, its effects were difficult to account for due to regional variability in implementation.^41^ Finally, the definition of early secondary brain insults—based on extreme physiological values within the first 24 h—could not provide insight into the duration of a patient's exposure to each brain insult. In addition, exposure to early secondary brain insults would have been underestimated for TBI patients subjected to very early WLST decision. More fundamentally, the observational nature of this study means that we cannot establish a causal inference between any single brain insult and hospital mortality.

For critically ill TBI patients, hospital mortality and WLST decisions have increased over the past 15 years in England, Wales and Northern Ireland. These trends appear to be independent of measured confounders. Our findings highlight the urgent need of further studies to better understand the decision-making process surrounding WLST. Specifically, future studies should aim to identify the prognostic factors, clinical tools, and underlying reasons that drive these critical decisions in daily practice. A notable proportion of patients were also exposed to early secondary brain insults, particularly hypoxaemia which has become increasingly common over time. This finding underscores the importance of preventing early secondary brain injury, which should be considered a key priority by healthcare policymakers and clinicians. It also raises important research questions about how specific types of secondary brain insult may influence patient prognosis.

## Contributors

XC, OH, DKM and DAH conceived and designed the study. XC, OH, DKM and DAH contributed to the methods of the study. XC and DAH performed statistical analysis, had full access to the original dataset and verified all the data. XC, OH, DKM and DAH interpreted the data. XC wrote the first draft of the manuscript. All authors contributed to the writing of the final version of the manuscript. All authors agreed with the results and conclusions of this manuscript. XC, OH, DKM and DAH had final responsibility for the decision to submit the paper. The corresponding author attests that all listed authors meet authorship criteria and that no others meeting the criteria have been omitted.

## Data sharing statement

Data are available upon reasonable request submitted via the TBI-REPORTER platform or directly through the ICNARC data request procedure (https://www.icnarc.org/data-services/access-our-data/) and subject to appropriate approvals.

## Declaration of interests

DKM reports research support from United Kingdom Research and Innovation (UKRI) and National Institute for Health and Care Research (NIHR) for the UK TBI-Repository and Data Portal Enabling Discovery (TBI-REPORTER) platform (grant ref: MR/Y008502/1), has received research support by GlaxoSmithKline, PressuraNeuro, Integra Neuroscience and Lantmannen AB, has received consulting fees from NeurotraumaSciences, Lantmannen AB, PressuraNeuro, Dompe and Invex Ltd and has received speaker fees from Integra Neuroscience. Other authors report no competing interest related to the submitted article.
