# Supplementary material for: Hospital mortality, withdrawal of life-sustaining therapy decisions and early secondary brain insults for critically ill traumatic brain injury patients in England, Wales and Northern Ireland (2009–2024): an observational cohort study
Source: Lancet Reg Health Eur. 2025 Nov 20;61:101538. doi: 10.1016/j.lanepe.2025.101538 (PMC12670143; doi:10.1016/j.lanepe.2025.101538)
Supplement: Supplementary Appendix [file mmc1.docx]

**Supplemental Appendix for:** Trends in hospital mortality, withdrawal of life-sustaining therapy decisions and early secondary brain insults for critically ill TBI patients over the past 15 years in England, Wales and Northern Ireland: An observational cohort study

**Authors:** Xavier Chapalain, Olivier Huet, Kathryn M Rowan, Paul R Mouncey, Olivier Langeron, David K Menon, David A Harrison

**Table of contents**

[Supplementary material 3](#_Toc210915445)

[Data source 3](#_Toc210915446)

[Patients’ selection 3](#_Toc210915447)

[Derived variables 4](#_Toc210915448)

[Primary and secondary outcomes definitions 4](#_Toc210915449)

[Exploratory analysis 4](#_Toc210915450)

[Covariates 5](#_Toc210915451)

[Rational for selection 5](#_Toc210915452)

[Definitions 7](#_Toc210915453)

[Covariates included in the multivariable analyses 8](#_Toc210915454)

[Statistical analyses 9](#_Toc210915455)

[Descriptive analysis 9](#_Toc210915456)

[Matching 9](#_Toc210915457)

[Collinearity 9](#_Toc210915458)

[Primary and secondary outcome analyses 9](#_Toc210915459)

[Sensitivity analysis 9](#_Toc210915460)

[Post-hoc analysis 10](#_Toc210915461)

[Software 10](#_Toc210915462)

[Supplemental results 11](#_Toc210915463)

[Table S1: Characteristics of the ICUs participating to the Case Mix Programme (CMP) over the study period. 11](#_Toc210915464)

[Table S2: Other patient-level characteristics for TBI patients 12](#_Toc210915465)

[Table S3: Patient- and centre-level characteristics for trauma patients (without TBI). 13](#_Toc210915466)

[Table S4: Patient- and centre-level characteristics for sepsis patients. 14](#_Toc210915467)

[Table S5: Patient- and centre-level characteristics for vascular brain injury patients. 15](#_Toc210915468)

[Table S6: Patient- and centre-level characteristics for other ICU patients. 16](#_Toc210915469)

[Table S7: Univariable and multivariable analysis representing association between main variables and hospital mortality 17](#_Toc210915470)

[Figure S1: Adjusted Odds Ratio (aOR) and 95% confidence intervals for continuous variables from the regression model analysing hospital mortality. 18](#_Toc210915471)

[Figure S2: Final model performances for hospital mortality. 19](#_Toc210915472)

[Table S8: Sensitivity analysis for hospital mortality on imputed dataset 20](#_Toc210915473)

[Figure S3: Percentage of hospital mortality and WLST decision for TBI patients and comparator cohorts 21](#_Toc210915474)

[Figure S4: Trends in hospital mortality (panel A, B, C) and WLST decision (panel D, E, F) in matched comparator cohorts. 22](#_Toc210915475)

[Figure S5: Trends in hospital mortality and WLST decision for TBI patients and other ICU patients (except those included in trauma, sepsis and vascular brain injury comparator cohorts) 23](#_Toc210915476)

[Table S9: Post-hoc sensitivity analysis for primary and secondary outcome restricted to centres recruited TBI patients all over the study period 24](#_Toc210915477)

[Figure S6: Trends in hospital mortality (panel A) and WLST decision (panel B) each year, before (2019), during (2020-2021) and after (2022-2024) COVID-19 pandemic 25](#_Toc210915478)

[Table S10: Univariable and multivariable analysis representing association between main variables and WLST 26](#_Toc210915479)

[Figure S7: Adjusted Odds Ratio (aOR) and 95% confidence intervals for continuous variables from the regression model analysing WLST decision. 27](#_Toc210915480)

[Figure S8: Final model performances for WLST decision 28](#_Toc210915481)

[Table S11: Sensitivity analysis for WLST decision 29](#_Toc210915482)

[Table S12: Main physiological parameters recorded during the first 24 hours following ICU admission. 30](#_Toc210915483)

[References 32](#_Toc210915484)

[Original Statistical Analysis Plan (SAP) 34](#_Toc210915485)

# Supplementary material

## Data source

All data presented in this cohort study were obtained from the Intensive Care National Audit & Research Centre (ICNARC) Case Mix Programme (CMP). The CMP is the national clinical audit for adult critical care in England, Wales, and Northern Ireland. Established 30 years ago, it has resulted in a high-quality, comprehensive clinical database. During the study period (1 April 2009 to 31 March 2024), the CMP achieved near-complete coverage of adult general critical care units delivering level 3 or combined level 2–3 care. In participating centres, all consecutive patients were included, and clinical data were prospectively collected. Trained data collectors abstracted information from medical records using predefined rules and standardised definitions. Throughout the study period, the definitions of outcomes and key covariates remained consistent. The data collected included sociodemographic characteristics, severe comorbidities, ICU admission routes, a range of physiological variables, and outcomes through to hospital discharge. Data were continuously verified locally using dedicated software systems, then centrally collated and subjected to a final validation process at ICNARC. Further details on data collection and validation have been published previously.^1^ The CMP database has also been independently evaluated by the Directory of Clinical Databases (DoCDat) against 10 domains assessing data coverage and accuracy.^1^

## Patients’ selection

All patients admitted to an adult general or neurosciences critical care unit participating in the CMP between 1 April 2009 and 31 March 2024 were included in the study. Patients included in this study were characterised according to the ICNARC coding method.^2^ This validated hierarchical coding method comprises five tiers, each corresponded to a specific clinical descriptor:

- *Type of admission*: surgical or medical
- *System*: body system involved
- *Site*: anatomical site involved
- *Process*: physiological or pathological process involved
- *Condition*: specific medical condition

Based on this classification system, patients were initially grouped into four mutually exclusive categories: ‘traumatic brain injury (TBI) patients’, ‘trauma patients (without TBI)’, ‘sepsis patients’ and ‘other ICU patients’.

TBI patients

TBI patients were identified in the database by any of the following conditions recorded as the primary or secondary reason for admission to the critical care unit: ‘primary brain injury’, ‘traumatic subdural haemorrhage’, ‘traumatic subarachnoid haemorrhage’, ‘extradural haemorrhage’, ‘focal brain injury’, ‘non accidental injury to brain’, ‘intracranial injury, unspecified’. During the course of data analysis, we introduced an additional comparator group: ‘vascular brain injury’ (e.g., stroke, intracranial haemorrhage and sub-arachnoid haemorrhage). This refinement was made to illustrate our findings within another neurological population likely to be subject to a similar decision-making process regarding withdrawal of life-sustaining therapy (WLST).

Trauma patients

Trauma patients were identified by any condition within the ‘Trauma’ process of the ICNARC coding method recorded as the primary or secondary reason for admission to the critical care unit, excluding those identified as TBI patients.

Sepsis patients

Sepsis patients were identified using a derived approximation of the Sepsis-2 and Sepsis-3 definitions, based on the recorded reason for ICU admission and physiological parameters during the first 24 hours, excluding any patients identified as TBI or trauma.^3 4^

Vascular brain injury patients

Vascular brain injury patients were identified by any of the following conditions recorded as the primary reason for admission to the critical care unit: ‘Non-traumatic subarachnoid haemorrhage’, ‘Non-traumatic subdural haemorrhage’, ‘Non-traumatic extradural haemorrhage’, ‘Intracerebral haemorrhage (supratentorial)’, ‘Intracerebral haemorrhage (posterior fossa)’, ‘Thrombo-occlusive disease of brain’, ‘Berry or other intracranial aneurysm’, ’Intracranial arterio-venous malformation’, ‘Venous air embolus’, ‘Vasculitis of cerebral circulation’ and ‘Embolic brain lesions’.

Other ICU patients

This group included all remaining patients who were not classified as having TBI, trauma, sepsis, or vascular brain injury according to the definitions above.

## Derived variables

Two prognostic scores were used to characterise the study population: the APACHE II (Acute Physiology and Chronic Health Evaluation) score and the core IMPACT (International Mission for Prognosis and Analysis of Clinical Trials) score. The APACHE II score was routinely calculated within the CMP using age, comorbidities and physiological data recorded during the first 24 hours.^5^ The IMPACT score was calculated using the core model developed by Steyerberg et al.^6^ In accordance with the original prognostic model, the core IMPACT score was based on three components: age, pupillary reactivity, and the motor component of the Glasgow Coma Scale (GCS).^6^

## Primary and secondary outcomes definitions

The primary outcome was hospital mortality, prospectively recorded in the CMP. This was defined as the ultimate vital status (‘died’ or ‘alive’) at hospital discharge, with patients transferred to another acute hospital followed up to final discharge from acute hospital.

The secondary outcome was the proportion of withdrawal of life-sustaining therapy (WLST) decisions. WLST was defined as the withdrawal of all clinically indicated treatments—except for comfort measures—on the grounds of a lack of expected benefit to the patient. This measure excluded decisions to withhold treatment, as documentation of therapeutic withholding was not consistent across the study period. Only documented decisions involving therapeutic withdrawal were considered.

## Exploratory analysis

The following predefined brain insults, based on physiological data recorded during the first 24 hours in ICU, were described: hypotension (defined by a lowest or a highest systolic arterial blood pressure < 100 mmHg), hypoxaemia (defined by a lowest PaO_2_ < 80 mmHg), hyperoxia (defined by a lowest PaO_2_ > 100 mmHg), hyponatremia (defined by a lowest or a highest sodium level < 135 mmol/l), hypernatremia (defined by a lowest or a highest sodium level > 145 mmol/l), hypothermia (defined by a lowest or a highest temperature < 36°C), hyperthermia (defined by a lowest or a highest temperature > 38°C), hypoglycaemia (defined by a lowest or a highest glucose level < 4.5 mmol/l), hyperglycaemia (defined by a lowest or a highest glucose level > 10 mmol/l), hypocapnia (defined by a PaCO_2_ < 35 mmHg), hypercapnia (defined by a PaCO_2_ > 45 mmHg), alkalosis (defined by a pH level, associated with lowest PaO_2_ > 7.45), acidosis (defined by a lowest pH level < 7.35), anaemia (defined by a lowest haemoglobin level < 7 g/dl), thrombopenia (defined by a lowest platelet count < 75 G/l). These thresholds were chosen based on international guidelines from the 2024 American College of Surgeon from their Trauma Quality Improvement Program.^7^ These guidelines delineate early management principles for TBI patients and are broadly recognised as a gold standard.

## Covariates

### Rational for selection

Covariates were selected based on prior results that reported association with mortality and poor neurological outcomes for TBI patients.^6 8^ Several of these variables have been incorporated into established prognostic models: the IMPACT model and the CRASH (Corticoid Randomization after Significant Head Injury) model.^9-11^ Both models were cited as key references in the recent *Lancet Neurology* commission on ‘Traumatic Brain Injury: progress and challenges in prevention, clinical care, and research’.^12^ Their predictive performances has been extensively described and reported.^9-11 13^ Details of the covariates included in each model are provided on the table below:

| **IMPACT models ^6^** | **Description of variables** |
| --- | --- |
| Core model | - Age: numeric age on admission - GCS Motor Score: Categorical variable (ranged from 1 to 6), resulted from neurological examination assessed on admission. If a patient is sedated or unable to be assessed, this evaluation is considered to be ‘not testable’, and this modality is considered by the prognostic model. - Pupillary reactivity: Categorical variable (Both reactive, one reactive or none reactive), resulted from neurological examination assessed on admission. |
| Extended model | *All variables from Core model, plus the additional features below:*   - Hypoxia: binary variable (Yes or No) - Hypotension: binary variable (Yes or No) - Marshall CT classification: Categorical variable (ranged from 1 to 6), scored on the admission brain CT scan according to the Marshall classification. - Traumatic subarachnoid haemorrhage: binary variable (Yes or No) - Epidural hematoma: binary variable (Yes or No) |
| Lab model | *All variables from Extended model, plus the additional features below:*   - Glucose level: glucose level measured on admission. - Haemoglobin level: haemoglobin level on admission |
| **CRASH models ^8^** | **Description of variables** |
| Basic model | - Age: numeric age on admission - GCS Total Score: Categorical variable (Ranged from 3 to 15), resulted from neurological examination measured on admission. - Pupillary reactivity: Categorical variable (Both reactive, one reactive or none reactive), resulted from neurological examination assessed on admission. - Major extracranial injury: Binary variable (Yes or No). Yes if score of ≥3 on at least one of the extracranial domains of the Abbreviated Injury Scale. |
| CT model | *All variables from Basic model, plus the additional features below:*   - Petechial haemorrhage: binary variable (Yes or No); Yes if evidence of shear trauma on CT - Obliteration of third ventricle or basal cisterns: binary variable (Yes or No) - Traumatic subarachnoid haemorrhage: binary variable (Yes or No) - Midline shift ≥ 5mm: binary variable (Yes or No) - Non-evacuated hematoma: binary variable (Yes or No) |

A priori, we selected covariates from the CMP dataset to reflect patient prognosis, drawing on established prognostic models such as IMPACT and CRASH.^6 8^ The following variables were included: Age, GCS motor component, pupil reactivity, lowest systolic arterial blood pressure (SAP), lowest partial pressure of oxygen (PaO_2_), lowest haemoglobin (Hb) level, lowest and highest glucose levels. Together, these seven covariates captured patient age, neurological status at ICU admission and key physiological parameters associated with outcomes.^6 8^ Although not included in previous prognostic models, we also considered the partial pressure of carbon dioxide (PaCO_2_), given its critical role in cerebral physiology. Arterial PaCO₂ influences cerebral blood flow through pH-mediated vasoreactivity: hypercapnia leads to cerebral vasodilation, which may increase intracranial pressure, whereas hypocapnia causes vasoconstriction, potentially leading to cerebral ischaemia.^14^ Partial pressure of CO_2_ (PaCO_2_) was only included in the model analysing the primary outcome (hospital mortality). Most of these physiological parameters (PaO_2_, SAP, Hb, PaCO_2_, glucose) are also recognised as therapeutic targets in international TBI management guidelines.^15 16^

In addition to these core variables, we included the following covariates in our models:

- Sex (male vs. female): Included in the hospital mortality model, based on evidence suggesting potential sex-based differences in TBI outcomes.^17-19^
- Deprivation index: This variable accounts for socioeconomic status, reflecting the level of deprivation in the patient's residential area prior to hospital admission. Its inclusion is supported by studies linking socioeconomic disadvantage to poorer TBI outcomes.^20-22^
- The level of dependence prior to ICU admission: Categorised as ‘no assistance’, ‘minor assistance’ and ‘total assistance’. We hypothesized that this variable is more a driver for WLST decision making process than mortality. So, we decided to only include this variable in the multivariable model analysing WLST.
- Surgery: This variable distinguishes between patients who underwent surgical management (either cranial or extracranial) and those who did not. It accounts for differing clinical trajectories (surgery: yes vs. no) and reflects the known association between extracranial injuries requiring surgery and worse neurological outcomes.^23^
- Type of ICU: Categorised as general ICU without neurosurgery, general ICU with neurosurgery, and specialist neuroscience ICU. This variable adjusts for differences in neurosurgical resources and neurocritical care expertise across centres.^24 25^
- The size of ICU: Initially considered as a proxy for unit workload and patient throughput, under the hypothesis that larger units might face capacity-related challenges affecting care quality. However, this variable was excluded from the final models due to multicollinearity with the ‘type of ICU’ variable.
- Admission hospital: To account for between-centre variability in clinical practice, a random effect for hospital site was included in the models.^25^

### Definitions

Covariates were extracted directly from the CMP database. Their exact definitions—based on the data collection manual used by trained data collectors—as well as their characteristics and coding modalities (with reference categories specified for categorical variables), are detailed in the table below:

| **Name** | **Definition** | **Type and modalities** | **Reference*** |
| --- | --- | --- | --- |
| Year of admission | Year of the date of ICU admission. | Categorical (fixed effect):  ‘2009-2010’, ‘2011-2012’,  ‘2013-2014’, ‘2015-2016’,  ‘2017-2018’, ‘2019-2020’,  ‘2021-2022’, ‘2023-2024’ | ‘2009-2010’ |
| Age | Age on ICU admission, calculated in regards to the known date of birth. If date of birth is unobtainable, clinical judgement was used to estimate age of admission. | Continuous | 50 years |
| Sex | Genotypical sex assigned at birth. | Categorical (fixed effect):  ‘male’, ‘female’ | ‘male’ |
| Level of dependence | The level of dependence in the two weeks prior to admission to acute hospital and prior to the onset of the acute illness. Assessed according the amount of assistance required with daily activities (e.g. bathing, dressing, going to the toilet, moving in and out of bed/chair, continence and eating). | Categorical (fixed effect):  ‘no assistance’, ‘some assistance’ and ‘total assistance’ | ‘no assistance’ |
| Deprivation index | This covariate is routinely derived in the CMP using the patient’s usual residential postcode at the time of ICU admission. Postcodes are mapped to regional deprivation scores using the Index of Multiple Deprivation 2010 (England), Welsh Index of Multiple Deprivation 2008, or Northern Ireland Multiple Deprivation Measure 2010. | Categorical (fixed effect): from 1 (least deprived) to 5 (most deprived) | 1 (least deprived) |
| Surgery | In-hospital surgery/procedure conducted within first 24 hours prior to ICU admission. | Categorical (fixed effect):  ‘Yes’, ‘No’ | ‘No’ |
| GCS motor component | This variable was derived from two original fields in the CMP: the recorded motor component of the Glasgow Coma Scale (GCS) and the presence or absence of sedation. The motor score was defined as the motor component from the lowest recorded total GCS during the first 24 hours in ICU. For patients who were sedated for the entire of the first 24 hours in ICU, the GCS was marked as ‘Untestable’. | Categorical (fixed effect): ‘Localises or obeys [5-6]’, ‘Normal flexion [4]’, ‘Abnormal flexion [3]’, ‘Extension [2]’, ‘None [1]’ and ‘Untestable’ | ‘Localises or obeys’ |
| Pupillary reactivity | The most abnormal pupil reactivity, for left and right eyes, assessed and recorded as a pair in the first 24 hours in ICU. | Categorical (fixed effect):  ‘Both reacting’,  ‘One reacting’,  ‘Both unreactive’ | ‘Both reacting’ |
| Lowest SAP | The lowest systolic arterial blood pressure (SAP) value recorded in the first 24 hours in ICU, irrespective of the measurement method. | Continuous | 100 mmHg |
| Lowest PaO_2_ | The lowest partial pressure of oxygen (PaO_2_) measured and recorded, on arterial blood gas, in the first 24 hours in ICU. | Continuous | 90 mmHg |
| PaCO_2_ | The partial pressure of carbon dioxide (PaCO_2_) from the arterial blood gas with the lowest PaO_2_ recorded during the first 24 hours in ICU. | Continuous | 40 mmHg |
| Lowest haemoglobin | The lowest haemoglobin value measured and recorded in the first 24 hours in ICU. | Continuous | 12 g/dl |
| Lowest glucose | The lowest serum glucose value measured and recorded in the first 24 hours in ICU. | Continuous | 6 mmol/l |
| Highest glucose | The highest serum glucose value measured and recorded in the first 24 hours in ICU. | Continuous | 9 mmol/l |
| Admission hospital | The hospital where the patient was admitted. | Categorical (random effect) | n/a |
| Type of ICU | This derived variable categorises ICUs based on their access to neurosurgical services. In hospitals with both a specialist neuroscience ICU and a general ICU, the general ICU was classified as ‘general ICU with neurosurgery’ for modelling purposes. | Categorical (fixed effect): ‘general ICU’, ‘general ICU with neurosurgery’, and ‘specialist neuroscience ICU’ | ‘general ICU’ |

n/a: not applicable

^*^For continuous variables, reference denotes mean values chosen to represent adjusted odds ratio for multivariable analysis on figure S1 and figure S9

### Covariates included in the multivariable analyses

For the primary outcome analysis (hospital mortality), we originally examined the effect of the following covariates: year of ICU admission (main independent variable), age, sex, deprivation index, surgery, GCS motor component, pupillary reactivity, lowest systolic arterial pressure (SAP), lowest partial pressure of oxygen (PaO₂), partial pressure of carbon dioxide (PaCO₂), lowest haemoglobin level, mean glucose level, type of ICU, and admitting hospital (as a random effect). During the course of the analysis, a post hoc decision was made to replace the mean glucose level with both the lowest and highest glucose levels, in order to better capture the occurrence and impact of hypoglycaemia and hyperglycaemia.

For the secondary outcome analysis (WLST), the initial model included the following covariates: year of ICU admission, age, level of dependence, surgery, GCS motor component, pupillary reactivity, type of ICU, and admitting hospital. After preliminary multivariable modelling, the initial specification was found to underperform, particularly in patients with a high probability of WLST. Consequently, a refined model was developed post hoc to enhance predictive performance. This final model included all original covariates plus additional variables: deprivation index, lowest SAP, lowest PaO₂, lowest haemoglobin level, lowest glucose level, and highest glucose level.

## Statistical analyses

### Descriptive analysis

Continuous variables were summarised as means and standard deviations (SD) or medians and interquartile ranges (IQR) depending on their distribution and clinical relevance. Categorical variables were described as counts and percentages. For primary and secondary outcomes measures (hospital mortality and WLST), 95% confidence intervals (95% CI) for proportions were calculated according to the Wald method.

### Matching

TBI patients were matched with trauma, vascular brain injury, sepsis and other ICU patients using a combined exact and nearest-neighbour matching approach, without replacement. Exact matching method was applied to two categorical variables: type of ICU and year of ICU admission. Nearest-neighbour matching was applied to two continuous variables: age and APACHE II score (after removing age points). Each TBI patient was initially intended to be matched with two trauma patients, two vascular brain injury patients, three sepsis patients, and five other ICU patients, according to the number of available potential matches. The matching procedure was implemented using the ‘MatchIt’ R package.

### Collinearity

Potential multicollinearity among candidate covariates was evaluated using correlation plots and variance inflation factors (VIFs). Collinearity was considered absent when pairwise correlation coefficients were < 0.8 and VIF values were < 2. ICU size was found to be collinear with ICU type and was therefore excluded from the final models. No other covariates exhibited collinearity.

### Primary and secondary outcome analyses

A generalised linear mixed model (multilevel logistic regression) was performed to analyse the relationship between hospital mortality and prespecified independent variables (year of admission, age, sex, deprivation index, surgery, GCS motor component, pupillary reactivity, lowest SAP, lowest PaO_2_, PaCO_2_, lowest Hb, lowest glucose, highest glucose, type of ICU, and admission hospital). The main independent variable was the year of ICU admission, considering the first study period (April 2009–2010) as reference. Univariable analysis was performed for each covariate and results were displayed with unadjusted odds ratio (OR) and 95% CI. For continuous variables (age, lowest SAP, mean PaO_2_, PaCO_2_, lowest Hb, lowest glucose, highest glucose), a restricted cubic spline method was performed to test non-linearity of their relationship with hospital mortality. Three, four and five knots were applied. The Akaike Information Criterion (AIC) and Bayesian Information Criterion (BIC) were compared, taking three-knots approach as reference. The best relationship was decided regarding model fitting, plausibility and previous knowledge. Multivariable analysis was performed including all covariates irrespective of their association with outcome in univariable analysis. In the final model, continuous variables were analysed with restricted cubic splines. Additionally, a random effect for the admission hospital was included in the final model. Results of multivariable analysis were displayed with adjusted ORs and their 95% CIs. For continuous variables, ORs and 95% CIs were plotted taking mean values for each covariate as reference. This analysis was performed on complete cases without imputation.

The secondary outcome of WLST was analysed using the same process of univariable and multivariable generalised linear mixed models.

For primary and secondary outcome analyses, discriminative performance of the final models was evaluated with the area under the receiver operating characteristic curve (AUC). Performance was also evaluated visually by plotting predictive probability of the corresponding events (hospital mortality or WLST) in the overall dataset stratified by events’ occurrence, and with calibration plots representing the relationship between observed and predicted probabilities.

Primary and secondary outcome analyses were performed with the ‘glmer’ function from the ‘lme4’ R package.

### Sensitivity analysis

A sensitivity analysis was performed on the imputed dataset. Five repeated multivariate imputations by Gibbs sampling were performed, using the ‘MICE’ R package. Results of the sensitivity analysis were reported as ORs and their 95% CIs.

### Post-hoc analysis

- Subgroup analysis:

We plotted the risk (aOR and 95% CI) for the primary and secondary outcomes in the following subgroups: age (<30 years, 30-60 years, > 60 years), the type of ICU (general ICU, general ICU with neurosurgery, specialist neuroscience ICU), predicted probability of poor neurological prognosis evaluated by the core IMPACT score (<40%, 40-60%, >60%). The same generalised mixed model used for the primary analysis were applied.

- COVID pandemic period:

We plotted the trend of the primary and secondary outcome each year from 2019 to 2024 to evaluate visually if outcomes were impact during COVID pandemic.

- Other analysis:

We described primary and secondary outcome measures in ICU recruited TBI patients throughout the entire study period.

### Software

All statistical analyses were performed with R statistical software (version 4.4.0).

# Supplemental results

## Table S1: Characteristics of the ICUs participating to the Case Mix Programme (CMP) over the study period.

| **Variables** | **April 2009**–**2010** | **2011**–**2012** | **2013**–**2014** | **2015**–**2016** | **2017**–**2018** | **2019**–**2020** | **2021**–**2022** | **2023**–  **March 2024** |
| --- | --- | --- | --- | --- | --- | --- | --- | --- |
| All hospitals and ICUs participating in the CMP |  |  |  |  |  |  |  |  |
| Number of hospitals | 202 | 210 | 218 | 221 | 223 | 222 | 218 | 218 |
| Number of ICUs | 220 | 229 | 246 | 257 | 268 | 283 | 279 | 269 |
| Hospitals and ICUs admitting TBI patients* |  |  |  |  |  |  |  |  |
| Number of hospitals | 186 | 192 | 200 | 198 | 197 | 196 | 187 | 187 |
| Type of hospital – no. |  |  |  |  |  |  |  |  |
| Non-university | 110 | 106 | 109 | 107 | 109 | 107 | 103 | 103 |
| University | 46 | 52 | 54 | 54 | 55 | 55 | 53 | 51 |
| University affiliated | 30 | 34 | 37 | 37 | 33 | 34 | 31 | 33 |
| Region of hospital – no. |  |  |  |  |  |  |  |  |
| Regions of England |  |  |  |  |  |  |  |  |
| East Of England | 16 | 18 | 18 | 18 | 18 | 18 | 18 | 18 |
| London | 19 | 24 | 28 | 30 | 32 | 27 | 30 | 29 |
| Midlands | 29 | 27 | 26 | 27 | 26 | 28 | 25 | 26 |
| North East and Yorkshire | 30 | 33 | 34 | 33 | 31 | 30 | 30 | 28 |
| North West | 24 | 24 | 27 | 25 | 24 | 26 | 23 | 23 |
| South East | 26 | 25 | 27 | 27 | 28 | 26 | 24 | 26 |
| South West | 17 | 18 | 19 | 16 | 18 | 18 | 17 | 16 |
| Wales | 16 | 14 | 13 | 13 | 13 | 15 | 12 | 13 |
| Northern Ireland | 9 | 9 | 8 | 9 | 7 | 8 | 8 | 8 |
| Number of ICUs* | 202 | 201 | 209 | 206 | 207 | 217 | 206 | 201 |
| Total number of ICU beds | 2,104 | 2,354 | 2,665 | 2,906 | 3 068 | 3 083 | 3 127 | 3 112 |
| Type of ICU |  |  |  |  |  |  |  |  |
| General ICU without neurosurgery – no. | 162 | 167 | 174 | 172 | 171 | 171 | 161 | 161 |
| Total number of ICU beds | 1,587 | 1,773 | 1,987 | 2,124 | 2 237 | 2 286 | 2 284 | 2 269 |
| Median number of beds/unit (IQR) | 9 (7–12) | 9 (7–13) | 10 (8–14) | 11 (8–15) | 12 (9–16) | 12 (9–16) | 13 (10–20) | 13 (10–16) |
| Number of TBI patients included | 1,549 | 1,870 | 1,685 | 2,083 | 2,082 | 1,907 | 1,772 | 1,114 |
| General ICU with neurosurgery – no. | 23 | 23 | 24 | 24 | 24 | 23 | 24 | 24 |
| Total number of ICU beds | 433 | 507 | 573 | 642 | 675 | 641 | 687 | 685 |
| Median number of beds/unit (IQR) | 18 (16–24) | 22 (17–26) | 23 (20–26) | 25 (21–34) | 25 (21–35) | 26 (22–33) | 27 (22–33) | 28 (24–33) |
| Number of TBI patients included | 1,834 | 2,349 | 2,979 | 3,320 | 3,632 | 3,209 | 3,064 | 1,849 |
| Specialist neuroscience ICU – no. | 6 | 5 | 7 | 8 | 9 | 9 | 9 | 9 |
| Total number of ICU beds | 84 | 74 | 105 | 140 | 156 | 156 | 156 | 158 |
| Median number of beds/unit (IQR) | 13 (12–15) | 14 (13–15) | 14 (13–18) | 18 (14–21) | 18 (14–20) | 18 (13–20) | 15 (14–20) | 17 (14–20) |
| Number of TBI patients included | 689 | 792 | 722 | 1,516 | 1,676 | 1,614 | 1,482 | 895 |

*Abbreviations:* CMP=Case Mix Programme; ICU=Intensive Care Unit; TBI=Traumatic Brain Injury

*Table legend:* *Number of hospitals and ICUs that recruited at least one TBI patient during the study period.

## Table S2: Other patient-level characteristics for TBI patients

| **Variables** | **Overall** | **April 2009**–**2010** | **2011**–**2012** | **2013**–**2014** | **2015**–**2016** | **2017**–**2018** | **2019**–**2020** | **2021**–**2022** | **2023**–  **March 2024** |
| --- | --- | --- | --- | --- | --- | --- | --- | --- | --- |
|  | N = 45,684 | N = 4,072 | N = 5,011 | N = 5,386 | N = 6,919 | N = 7,390 | N = 6,730 | N = 6,318 | N = 3,858 |
| Age category – no. (%) |  |  |  |  |  |  |  |  |  |
| <30 | 9,626 (21.1) | 1,198 (29.4) | 1,307 (26.1) | 1,155 (21.4) | 1,502 (21.7) | 1,433 (19.4) | 1,209 (18) | 1,169 (18.5) | 653 (16.9) |
| 30–60 | 20,384 (44.6) | 1,864 (45.8) | 2,261 (45.1) | 2,399 (44.5) | 2,982 (43.1) | 3,309 (44.8) | 3,034 (45.1) | 2,856 (45.2) | 1,679 (43.5) |
| >60 | 15,674 (34.3) | 1,010 (24.8) | 1,443 (28.8) | 1,832 (34.0) | 2,435 (35.2) | 2,648 (35.8) | 2,487 (37) | 2,293 (36.3) | 1,526 (39.6) |
| Sex – no. (%) |  |  |  |  |  |  |  |  |  |
| Male | 33,628 (73.6) | 3,079 (75.6) | 3,813 (76.1) | 3,975 (73.8) | 5,088 (73.5) | 5,364 (72.6) | 4,880 (72.5) | 4,624 (73.2) | 2,805 (72.7) |
| Female | 12,056 (26.4) | 993 (24.4) | 1,198 (23.9) | 1,411 (26.2) | 1,831 (26.5) | 2,026 (27.4) | 1,850 (27.5) | 1,693 (26.8) | 1,053 (27.3) |
| Ethnic category – no. (%) |  |  |  |  |  |  |  |  |  |
| White | 38,041 (83.3) | 3,563 (87.5) | 4,379 (87.4) | 4,617 (85.7) | 5,940 (85.9) | 6,296 (85.2) | 5,557 (82.6) | 4,858 (76.9) | 2,831 (73.4) |
| Mixed | 123 (0.3) | 7 (0.2) | 13 (0.3) | 19 (0.4) | 22 (0.3) | 24 (0.3) | 9 (0.1) | 18 (0.3) | 11 (0.3) |
| Asian | 1,796 (3.9) | 157 (3.9) | 162 (3.2) | 194 (3.6) | 268 (3.9) | 324 (4.4) | 277 (4.1) | 250 (4.0) | 164 (4.3) |
| Black | 877 (1.9) | 68 (1.7) | 92 (1.8) | 109 (2.0) | 145 (2.1) | 133 (1.8) | 130 (1.9) | 126 (2.0) | 74 (1.9) |
| Other ethnic group | 1,856 (4.1) | 78 (1.9) | 182 (3.6) | 244 (4.5) | 232 (3.4) | 275 (3.7) | 290 (4.3) | 307 (4.9) | 248 (6.4) |
| Not stated | 2,969 (6.5) | 190 (4.7) | 170 (3.4) | 203 (3.8) | 312 (4.5) | 338 (4.6) | 467 (6.9) | 759 (12) | 530 (13.7) |
| APACHE II score – no. (%) |  |  |  |  |  |  |  |  |  |
| <10 | 13,434 (31.1) | 1,211 (33) | 1,584 (34.7) | 1,549 (30.4) | 2,075 (31.5) | 2,069 (29.2) | 1,952 (30.2) | 1,908 (31.2) | 1,086 (29.5) |
| 10–14 | 13,253 (30.6) | 1,107 (30.2) | 1,333 (29.2) | 1,550 (30.4) | 1,994 (30.2) | 2,177 (30.8) | 2,000 (30.9) | 1,966 (32.1) | 1,126 (30.5) |
| 15–20 | 9,102 (21.0) | 810 (22.1) | 899 (19.7) | 1,067 (21.0) | 1,360 (20.6) | 1,537 (21.7) | 1,351 (20.9) | 1,258 (20.6) | 820 (22.2) |
| >20 | 7,464 (17.3) | 537 (14.7) | 747 (16.4) | 926 (18.2) | 1,163 (17.6) | 1,291 (18.2) | 1,162 (18.0) | 984 (16.1) | 654 (17.7) |
| CPR before ICU admission – no. (%) |  |  |  |  |  |  |  |  |  |
| No CPR | 43,092 (94.3) | 3,938 (96.7) | 4,794 (95.7) | 5,109 (94.9) | 6,571 (95) | 6,962 (94.2) | 6,297 (93.6) | 5,864 (92.8) | 3,557 (92.2) |
| Community CPR | 2,078 (4.5) | 104 (2.6) | 147 (2.9) | 191 (3.5) | 271 (3.9) | 361 (4.9) | 351 (5.2) | 386 (6.1) | 267 (6.9) |
| In-hospital CPR | 514 (1.1) | 30 (0.7) | 70 (1.4) | 86 (1.6) | 77 (1.1) | 67 (0.9) | 82 (1.2) | 68 (1.1) | 34 (0.9) |

*Abbreviations:* APACHE= Acute Physiology and Chronic Health Evaluation; CPR=Cardiopulmonary Resuscitation; ICU=Intensive Care Unit; TBI=Traumatic Brain Injury

## Table S3: Patient- and centre-level characteristics for trauma patients (without TBI).

| **Variables** | **Overall** | **April 2009**–**2010** | **2011**–**2012** | **2013**–**2014** | **2015**–**2016** | **2017**–**2018** | **2019**–**2020** | **2021**–**2022** | **2023**–  **March 2024** |
| --- | --- | --- | --- | --- | --- | --- | --- | --- | --- |
|  | N = 166,617 | N = 20,157 | N = 26,888 | N = 29,210 | N = 23,923 | N = 21,106 | N = 18,445 | N = 16,064 | N = 10,824 |
| **Patient-level factors** |  |  |  |  |  |  |  |  |  |
| Age, years |  |  |  |  |  |  |  |  |  |
| Mean (SD) | 60.7 (20.6) | 60.7 (20.3) | 61.6 (20.1) | 62.4 (19.9) | 60.7 (21.1) | 59.7 (21.5) | 59.8 (21.3) | 58.9 (20.8) | 59.9 (20.1) |
| Median (IQR) | 65 (46–77) | 65 (47–77) | 66 (48–78) | 67 (49–78) | 65 (45–78) | 63 (43–78) | 63 (44–77) | 62 (43–76) | 63 (45–76) |
| Age category – no. (%) |  |  |  |  |  |  |  |  |  |
| <30 | 18,527 (11.1) | 2,215 (11.0) | 2,661 (9.9) | 2,687 (9.2) | 2,812 (11.8) | 2,733 (12.9) | 2,304 (12.5) | 1,991 (12.4) | 1,124 (10.4) |
| 30–60 | 53,680 (32.2) | 6,208 (30.8) | 8,255 (30.7) | 8,886 (30.4) | 7,651 (32.0) | 7,072 (33.5) | 6,202 (33.6) | 5,637 (35.1) | 3,769 (34.8) |
| >60 | 94,404 (56.7) | 11,730 (58.2) | 15,971 (59.4) | 17,637 (60.4) | 13,460 (56.3) | 11,300 (53.5) | 9,939 (53.9) | 8,436 (52.5) | 5,931 (54.8) |
| Sex – no. (%) |  |  |  |  |  |  |  |  |  |
| Male | 97,327 (58.4) | 11,249 (55.8) | 14,929 (55.5) | 16,199 (55.5) | 13,942 (58.3) | 12,775 (60.5) | 11,333 (61.4) | 10,161 (63.3) | 6,739 (62.3) |
| Female | 69,290 (41.6) | 8,908 (44.2) | 11,959 (44.5) | 13,011 (44.5) | 9,981 (41.7) | 8,331 (39.5) | 7,112 (38.6) | 5,903 (36.7) | 4,085 (37.7) |
| APACHE II score – no. (%) |  |  |  |  |  |  |  |  |  |
| <10 | 42,095 (26.0) | 4,283 (22.1) | 5,830 (22.4) | 6,533 (23.1) | 6,291 (27.1) | 6,049 (29.3) | 5,377 (29.9) | 4,697 (29.9) | 3,035 (28.7) |
| 10–14 | 52,151 (32.2) | 6,017 (31.1) | 8,117 (31.2) | 9,006 (31.9) | 7,649 (32.9) | 6,794 (33) | 5,815 (32.3) | 5,240 (33.3) | 3,513 (33.2) |
| 15–20 | 44,855 (27.7) | 5,724 (29.6) | 7,731 (29.8) | 8,267 (29.2) | 6,306 (27.1) | 5,338 (25.9) | 4,735 (26.3) | 3,985 (25.3) | 2,769 (26.2) |
| >20 | 22,654 (14.0) | 3,322 (17.2) | 4,303 (16.6) | 4,469 (15.8) | 2,991 (12.9) | 2,432 (11.8) | 2,081 (11.6) | 1,799 (11.4) | 1,257 (11.9) |
| Dependence category – no. (%) |  |  |  |  |  |  |  |  |  |
| Without assistance | 126,443 (76.6) | 15,714 (78.7) | 20,701 (77.8) | 22,149 (76.5) | 17,928 (75.5) | 15,977 (76.2) | 14,082 (76.8) | 12,261 (77.0) | 7,631 (71.5) |
| Some assistance | 36,825 (22.3) | 4,129 (20.7) | 5,733 (21.5) | 6,614 (22.8) | 5,607 (23.6) | 4,859 (23.2) | 4,087 (22.3) | 3,314 (20.8) | 2,482 (23.3) |
| Total assistance | 1,894 (1.1) | 118 (0.6) | 187 (0.7) | 194 (0.7) | 201 (0.8) | 137 (0.7) | 164 (0.9) | 340 (2.1) | 553 (5.2) |
| Deprivation index***** – no. (%) |  |  |  |  |  |  |  |  |  |
| 1 (least deprived) | 26,651 (16.3) | 3,286 (16.5) | 4,442 (16.7) | 4,773 (16.6) | 3,792 (16.2) | 3,317 (16.1) | 2,875 (15.9) | 2,498 (15.9) | 1,668 (15.8) |
| 2 | 30,692 (18.8) | 3,738 (18.8) | 5,033 (19.0) | 5,407 (18.8) | 4,436 (18.9) | 3,806 (18.4) | 3,390 (18.8) | 2,894 (18.5) | 1,988 (18.9) |
| 3 | 33,598 (20.5) | 4,032 (20.3) | 5,450 (20.5) | 5,902 (20.5) | 4,806 (20.5) | 4,224 (20.5) | 3,799 (21.1) | 3,290 (21.0) | 2,095 (19.9) |
| 4 | 34,717 (21.2) | 4,252 (21.4) | 5,519 (20.8) | 5,955 (20.7) | 5,007 (21.4) | 4,523 (21.9) | 3,832 (21.3) | 3,369 (21.5) | 2,260 (21.5) |
| 5 (most deprived) | 37,946 (23.2) | 4,603 (23.1) | 6,111 (23.0) | 6,797 (23.6) | 5,401 (23.0) | 4,762 (23.1) | 4,136 (22.9) | 3,618 (23.1) | 2,518 (23.9) |
| **Centre-level factors** |  |  |  |  |  |  |  |  |  |
| Type of hospital – no. (%) |  |  |  |  |  |  |  |  |  |
| Non-university | 68,037 (40.8) | 10,582 (52.5) | 12,529 (46.6) | 12,724 (43.6) | 9,193 (38.4) | 7,511 (35.6) | 6,441 (34.9) | 5,127 (31.9) | 3,930 (36.3) |
| University | 71,431 (42.9) | 6,063 (30.1) | 9,603 (35.7) | 11,258 (38.5) | 10,955 (45.8) | 10,336 (49.0) | 9,256 (50.2) | 8,674 (54.0) | 5,286 (48.8) |
| University affiliated | 27,149 (16.3) | 3,512 (17.4) | 4,756 (17.7) | 5,228 (17.9) | 3,775 (15.8) | 3,259 (15.4) | 2,748 (14.9) | 2,263 (14.1) | 1,608 (14.9) |
| **Outcomes – no. (%)** |  |  |  |  |  |  |  |  |  |
| In-hospital mortality | 26,579 (16.0) | 4,150 (20.7) | 4,999 (18.7) | 5,116 (17.6) | 3,438 (14.4) | 2 619 (12.5) | 2,521 (13.7) | 2,261 (14.2) | 1,475 (13.8) |
| WLST decision | 9,976 (6.0) | 1,471 (7.3) | 1,884 (7.0) | 1,801 (6.2) | 1,218 (5.1) | 1,004 (4.8) | 992 (5.4) | 964 (6.0) | 638 (5.9) |

*Abbreviations:* APACHE= Acute Physiology and Chronic Health Evaluation; IQR=Interquartile Range; SD=Standard Deviation; TBI=Traumatic Brain Injury; WLST=Withdrawal of Life-Sustaining Therapy. *Table legend:* *****Derived from the patient’s usual residential postcode according to the Index of Multiple Deprivation 2010 for England, Welsh Index of Multiple Deprivation 2008 or Northern Ireland Multiple Deprivation Measure 2010.

## Table S4: Patient- and centre-level characteristics for sepsis patients.

| **Variables** | **Overall** | **April 2009**–**2010** | **2011**–**2012** | **2013**–**2014** | **2015**–**2016** | **2017**–**2018** | **2019**–**2020** | **2021**–**2022** | **2023**–  **March 2024** |
| --- | --- | --- | --- | --- | --- | --- | --- | --- | --- |
|  | N = 601,740 | N = 45,180 | N = 61,159 | N = 70,637 | N = 87,520 | N = 90,766 | N = 97,933 | N = 96,245 | N = 52,300 |
| **Patient-level factors** |  |  |  |  |  |  |  |  |  |
| Age, years |  |  |  |  |  |  |  |  |  |
| Mean (SD) | 62.1 (16.6) | 61.7 (17.6) | 62.8 (17.3) | 63.3 (17.0) | 63 (16.8) | 62.8 (16.6) | 61.7 (15.7) | 60.1 (15.9) | 61.6 (16.2) |
| Median (IQR) | 65 (52–75) | 65 (47–77) | 66 (48–78) | 67 (49–78) | 65 (45–78) | 63 (43–78) | 63 (44–77) | 62 (43–76) | 63 (45–76) |
| Age category – no. (%) |  |  |  |  |  |  |  |  |  |
| <30 | 29,129 (4.8) | 2,797 (6.2) | 3,368 (5.5) | 3,524 (5.0) | 4,239 (4.8) | 4,305 (4.7) | 3,966 (4.0) | 4,471 (4.6) | 2,459 (4.7) |
| 30–60 | 212,790 (35.4) | 15,202 (33.7) | 19,406 (31.7) | 22,232 (31.5) | 28,676 (32.8) | 30,498 (33.6) | 37,506 (38.3) | 39,867 (41.4) | 19,403 (37.1) |
| >60 | 359,810 (59.8) | 27,176 (60.2) | 38,382 (62.8) | 44,881 (63.5) | 54,602 (62.4) | 55,963 (61.7) | 56,461 (57.7) | 51,907 (53.9) | 30,438 (58.2) |
| Sex – no. (%) |  |  |  |  |  |  |  |  |  |
| Male | 263,204 (43.7) | 24,843 (55.0) | 33,578 (54.9) | 38,803 (54.9) | 48,144 (55) | 50,116 (55.2) | 57,180 (58.4) | 56,359 (58.6) | 29,512 (56.4) |
| Female | 338,535 (56.3) | 20,337 (45.0) | 27,581 (45.1) | 31,834 (45.1) | 39,376 (45) | 40,650 (44.8) | 40,753 (41.6) | 39,885 (41.4) | 22,788 (43.6) |
| APACHE II score – no. (%) |  |  |  |  |  |  |  |  |  |
| <10 | 48,719 (8.3) | 2,815 (6.5) | 3,652 (6.2) | 4,621 (6.8) | 6,577 (7.8) | 7,228 (8.2) | 9,191 (9.6) | 10,064 (10.7) | 4,571 (9) |
| 10–14 | 143,473 (24.6) | 8,785 (20.4) | 11,987 (20.5) | 14,366 (21.1) | 19,314 (22.8) | 21,106 (23.9) | 26,947 (28.2) | 28,231 (30) | 12,737 (25) |
| 15–20 | 204,977 (35.1) | 14,801 (34.3) | 20,502 (35.0) | 23,755 (34.9) | 29,892 (35.2) | 31,372 (35.6) | 34,159 (35.7) | 32,310 (34.3) | 18,186 (35.6) |
| >20 | 186,436 (31.9) | 16,695 (38.7) | 22,467 (38.3) | 25,308 (37.2) | 29,043 (34.2) | 28,480 (32.3) | 25,373 (26.5) | 23,541 (25.0) | 15,529 (30.4) |
| Dependence category – no. (%) |  |  |  |  |  |  |  |  |  |
| Without assistance | 414,784 (69.2) | 30,745 (68.4) | 41,161 (67.6) | 46,358 (65.9) | 58,602 (67.2) | 61,330 (67.8) | 71,304 (73.1) | 71,681 (75) | 33,603 (64.7) |
| Some assistance | 170,025 (28.4) | 13,389 (29.8) | 18,527 (30.4) | 22,601 (32.1) | 26,808 (30.7) | 27,430 (30.3) | 24,711 (25.3) | 21,534 (22.5) | 15,025 (28.9) |
| Total assistance | 14,169 (2.4) | 791 (1.8) | 1,172 (1.9) | 1,377 (2.0) | 1,808 (2.1) | 1,763 (1.9) | 1,558 (1.6) | 2,404 (2.5) | 3,296 (6.3) |
| Deprivation index***** – no. (%) |  |  |  |  |  |  |  |  |  |
| 1 (least deprived) | 88,987 (15.0) | 6,759 (15.1) | 9,267 (15.2) | 10,662 (15.2) | 13,359 (15.4) | 13,693 (15.3) | 14,058 (14.5) | 13,484 (14.2) | 7,705 (15.1) |
| 2 | 102,784 (17.3) | 7,719 (17.2) | 10,450 (17.2) | 12,119 (17.3) | 15,170 (17.5) | 16,018 (17.9) | 16,552 (17.1) | 15,822 (16.7) | 8,934 (17.5) |
| 3 | 115,886 (19.5) | 8,646 (19.3) | 11,765 (19.4) | 13,412 (19.1) | 17,064 (19.7) | 17,531 (19.5) | 18,905 (19.6) | 18,460 (19.5) | 10,103 (19.8) |
| 4 | 131,712 (22.2) | 9,587 (21.4) | 13,412 (22.1) | 15,271 (21.8) | 18,874 (21.8) | 19,731 (22.0) | 21,655 (22.4) | 21,611 (22.8) | 11,571 (22.7) |
| 5 (most deprived) | 155,032 (26.1) | 12,169 (27.1) | 15,877 (26.1) | 18,622 (26.6) | 22,160 (25.6) | 22,734 (25.3) | 25,504 (26.4) | 25,286 (26.7) | 12,680 (24.9) |
| **Centre-level factors** |  |  |  |  |  |  |  |  |  |
| Type of hospital – no. (%) |  |  |  |  |  |  |  |  |  |
| Non-university | 294,841 (49.0) | 23,218 (51.4) | 30,176 (49.3) | 33,861 (47.9) | 42,490 (48.5) | 45,413 (50.0) | 47,796 (48.8) | 46,013 (47.8) | 25,874 (49.5) |
| University | 203,699 (33.9) | 13,841 (30.6) | 19,829 (32.4) | 23,246 (32.9) | 29,810 (34.1) | 30,274 (33.4) | 33,876 (34.6) | 34,807 (36.2) | 18,016 (34.4) |
| University affiliated | 103,200 (17.2) | 8,121 (18.0) | 11,154 (18.2) | 13,530 (19.2) | 15,220 (17.4) | 15,079 (16.6) | 16,261 (16.6) | 15,425 (16.0) | 8,410 (16.1) |
| **Outcome – no. (%)** |  |  |  |  |  |  |  |  |  |
| In-hospital mortality | 185,720 (31.0) | 16,702 (37.2) | 21,040 (34.5) | 22,767 (32.3) | 26,383 (30.2) | 25,500 (28.2) | 29,613 (30.4) | 29,911 (31.3) | 13,804 (26.6) |
| WLST decision | 82,053 (13.6) | 6,596 (14.6) | 8,901 (14.6) | 9,464 (13.4) | 11,272 (12.9) | 11,465 (12.6) | 13,596 (13.9) | 13,883 (14.4) | 6,851 (13.1) |

*Abbreviations:* APACHE= Acute Physiology and Chronic Health Evaluation; IQR=Interquartile Range; SD=Standard Deviation; TBI=Traumatic Brain Injury; WLST=Withdrawal of Life-Sustaining Therapy. *Table legend:* *****Derived from the patient’s usual residential postcode according to the Index of Multiple Deprivation 2010 for England, Welsh Index of Multiple Deprivation 2008 or Northern Ireland Multiple Deprivation Measure 2010.

## Table S5: Patient- and centre-level characteristics for vascular brain injury patients.

| **Variables** | **Overall** | **April 2009**–**2010** | **2011**–**2012** | **2013**–**2014** | **2015**–**2016** | **2017**–**2018** | **2019**–**2020** | **2021**–**2022** | **2023**–  **March 2024** |
| --- | --- | --- | --- | --- | --- | --- | --- | --- | --- |
|  | N = 86,663 | N = 6,802 | N = 9,239 | N = 10,741 | N = 12,955 | N = 13,432 | N = 13,164 | N = 12,485 | N = 7,845 |
| **Patient-level factors** |  |  |  |  |  |  |  |  |  |
| Age, years |  |  |  |  |  |  |  |  |  |
| Mean (SD) | 59 (14.8) | 57.8 (15.3) | 58.5 (14.9) | 58.9 (15.1) | 59.2 (14.7) | 59.6 (14.9) | 59.2 (14.5) | 58.7 (14.4) | 59.1 (14.5) |
| Median (IQR) | 60 (49–70) | 59 (47–69) | 59 (48–70) | 60 (49–70) | 60 (49–70) | 60 (50–71) | 60 (50–70) | 59 (49–69) | 60 (50–70) |
| Age category – no. (%) |  |  |  |  |  |  |  |  |  |
| <30 | 3,012 (3.5) | 301 (4.4) | 338 (3.7) | 385 (3.6) | 431 (3.3) | 448 (3.3) | 437 (3.3) | 412 (3.3) | 260 (3.3) |
| 30–60 | 42,080 (48.6) | 3,404 (50.0) | 4,523 (49.0) | 5,185 (48.3) | 6,252 (48.3) | 6,361 (47.4) | 6,422 (48.8) | 6,189 (49.6) | 3,744 (47.7) |
| >60 | 41,571 (48.0) | 3,097 (45.5) | 4,378 (47.4) | 5,171 (48.1) | 6,272 (48.4) | 6,623 (49.3) | 6,305 (47.9) | 5,884 (47.1) | 3,841 (49.0) |
| Sex – no. (%) |  |  |  |  |  |  |  |  |  |
| Male | 41,934 (48.4) | 3,360 (49.4) | 4,439 (48) | 5,135 (47.8) | 6,199 (47.9) | 6,472 (48.2) | 6,455 (49.0) | 6,070 (48.6) | 3,804 (48.5) |
| Female | 44,729 (51.6) | 3,442 (50.6) | 4,800 (52) | 5,606 (52.2) | 6,756 (52.1) | 6,960 (51.8) | 6,709 (51.0) | 6,415 (51.4) | 4,041 (51.5) |
| APACHE II score – no. (%) |  |  |  |  |  |  |  |  |  |
| <10 | 19,045 (23.2) | 1,263 (20.5) | 1,910 (22.4) | 2,401 (23.8) | 2,977 (24.1) | 2,942 (23) | 2,945 (23.4) | 2,887 (24) | 1,720 (23.0) |
| 10–14 | 26,116 (31.9) | 1,777 (28.9) | 2,551 (30.0) | 3,062 (30.4) | 3,877 (31.4) | 4,069 (31.8) | 4,158 (33.0) | 4,174 (34.8) | 2,448 (32.7) |
| 15–20 | 18,648 (22.7) | 1,518 (24.7) | 1,973 (23.2) | 2,281 (22.7) | 2,804 (22.7) | 2,904 (22.7) | 2,792 (22.1) | 2,651 (22.1) | 1,725 (23.1) |
| >20 | 18,178 (22.2) | 1,589 (25.9) | 2,076 (24.4) | 2,324 (23.1) | 2,707 (21.9) | 2,886 (22.5) | 2,714 (21.5) | 2,294 (19.1) | 1,588 (21.2) |
| Dependence category – no. (%) |  |  |  |  |  |  |  |  |  |
| Without assistance | 76,168 (89.0) | 5,892 (88.5) | 8,053 (88.7) | 9,411 (88.7) | 11,394 (88.7) | 11,944 (89.5) | 11,788 (89.9) | 11,044 (90.0) | 6,642 (87.1) |
| Some assistance | 8,936 (10.4) | 743 (11.2) | 984 (10.8) | 1,174 (11.1) | 1,409 (11.0) | 1,355 (10.2) | 1,283 (9.8) | 1,146 (9.3) | 842 (11.0) |
| Total assistance | 436 (0.5) | 22 (0.3) | 37 (0.4) | 25 (0.2) | 45 (0.4) | 41 (0.3) | 39 (0.3) | 87 (0.7) | 140 (1.8) |
| Deprivation index***** – no. (%) |  |  |  |  |  |  |  |  |  |
| 1 (least deprived) | 14,213 (16.7) | 1,159 (17.3) | 1,499 (16.5) | 1,742 (16.5) | 2,084 (16.4) | 2,255 (17.1) | 2,130 (16.5) | 2,070 (17.0) | 1,274 (16.7) |
| 2 | 15,888 (18.7) | 1,236 (18.4) | 1,739 (19.1) | 1,900 (18.0) | 2,319 (18.2) | 2,525 (19.1) | 2,438 (18.9) | 2,272 (18.7) | 1,459 (19.1) |
| 3 | 16,855 (19.8) | 1,336 (19.9) | 1,828 (20.1) | 2,101 (19.9) | 2,638 (20.7) | 2,559 (19.4) | 2,570 (19.9) | 2,408 (19.8) | 1,415 (18.5) |
| 4 | 18,083 (21.3) | 1,376 (20.5) | 1,978 (21.7) | 2,268 (21.4) | 2,682 (21.1) | 2,801 (21.2) | 2,758 (21.4) | 2,588 (21.3) | 1,632 (21.4) |
| 5 (most deprived) | 19,934 (23.5) | 1,594 (23.8) | 2,055 (22.6) | 2,568 (24.3) | 3,007 (23.6) | 3,048 (23.1) | 2,996 (23.2) | 2,812 (23.1) | 1,854 (24.3) |
| **Center-level factors** |  |  |  |  |  |  |  |  |  |
| Type of hospital – no. (%) |  |  |  |  |  |  |  |  |  |
| Non-university | 20,128 (23.2) | 2,112 (31.0) | 2,289 (24.8) | 2,604 (24.2) | 3,003 (23.2) | 2,961 (22.0) | 2,795 (21.2) | 2,598 (20.8) | 1,766 (22.5) |
| University | 51,186 (59.1) | 3,587 (52.7) | 5,588 (60.5) | 6,244 (58.1) | 7,597 (58.6) | 8,001 (59.6) | 7,898 (60.0) | 7,681 (61.5) | 4,590 (58.5) |
| University affiliated | 15,349 (17.7) | 1,103 (16.2) | 1,362 (14.7) | 1,893 (17.6) | 2,355 (18.2) | 2,470 (18.4) | 2,471 (18.8) | 2,206 (17.7) | 1,489 (19.0) |
| **Outcome – no. (%)** |  |  |  |  |  |  |  |  |  |
| In-hospital mortality | 32,023 (37.4) | 2,797 (41.6) | 3,544 (38.9) | 3,921 (36.8) | 4,459 (34.6) | 4,733 (35.5) | 4,800 (36.8) | 4,668 (38.0) | 3,101 (40.7) |
| WLST decision | 13,982 (16.1) | 1,024 (15.1) | 1,434 (15.5) | 1,656 (15.4) | 1,914 (14.8) | 2,126 (15.8) | 2,150 (16.3) | 2,124 (17.0) | 1,553 (19.8) |

*Abbreviations:* APACHE= Acute Physiology and Chronic Health Evaluation; IQR=Interquartile Range; SD=Standard Deviation; TBI=Traumatic Brain Injury; WLST=Withdrawal of Life-Sustaining Therapy. *Table legend:* *****Derived from the patient’s usual residential postcode according to the Index of Multiple Deprivation 2010 for England, Welsh Index of Multiple Deprivation 2008 or Northern Ireland Multiple Deprivation Measure 2010.

## Table S6: Patient- and centre-level characteristics for other ICU patients.

| **Variables** | **Overall** | **April 2009**–  **2010** | **2011**–**2012** | **2013**–**2014** | **2015**–**2016** | **2017**–**2018** | **2019**–**2020** | **2021**–**2022** | **2023**–  **March 2024** |
| --- | --- | --- | --- | --- | --- | --- | --- | --- | --- |
|  | N = 1,245,079 | N = 102,386 | N = 148,057 | N = 163,583 | N = 189,544 | N = 195,847 | N = 176,151 | N = 161,727 | N = 107,784 |
| **Patient-level factors** |  |  |  |  |  |  |  |  |  |
| Age, years |  |  |  |  |  |  |  |  |  |
| Mean (SD) | 60.0 (18.0) | 60.4 (18.4) | 60.4 (18.3) | 60.7 (18.2) | 60.2 (18.1) | 60 (18.1) | 59.6 (17.9) | 59.5 (17.6) | 59.5 (17.7) |
| Median (IQR) | 63 (48–74) | 64 (48–75) | 64 (48–75) | 64 (49–75) | 64 (49–74) | 63 (48–74) | 63 (48–73) | 62 (48–73) | 62 (48–74) |
| Age category – no. (%) |  |  |  |  |  |  |  |  |  |
| <30 | 96,446 (7.7) | 8,196 (8.0) | 11,953 (8.1) | 12,582 (7.7) | 14,831 (7.8) | 15,530 (7.9) | 13,597 (7.7) | 11,810 (7.3) | 7,947 (7.4) |
| 30–60 | 456,608 (36.7) | 35,179 (34.4) | 51,203 (34.6) | 56,932 (34.8) | 68,360 (36.1) | 72,372 (37.0) | 67,376 (38.2) | 63,140 (39.0) | 42,046 (39) |
| >60 | 691,992 (55.6) | 59,001 (57.6) | 84,892 (57.3) | 94,065 (57.5) | 106,347 (56.1) | 107,943 (55.1) | 95,176 (54.0) | 86,777 (53.7) | 57,791 (53.6) |
| Sex – no. (%) |  |  |  |  |  |  |  |  |  |
| Male | 681,670 (54.7) | 56,371 (55.1) | 81,493 (55.0) | 89,157 (54.5) | 103,299 (54.5) | 106,440 (54.3) | 96,576 (54.8) | 89,000 (55.0) | 59,334 (55.0) |
| Female | 563,405 (45.3) | 46,015 (44.9) | 66,564 (45.0) | 74,426 (45.5) | 86,244 (45.5) | 89,407 (45.7) | 79,575 (45.2) | 72,726 (45.0) | 48,448 (44.9) |
| APACHE II score – no. (%) |  |  |  |  |  |  |  |  |  |
| <10 | 279,595 (23.3) | 20,742 (21.4) | 31,408 (22.2) | 35,614 (22.7) | 43,729 (23.9) | 45,964 (24.2) | 41,645 (24.4) | 36,398 (23.2) | 24,095 (23.0) |
| 10–14 | 418,395 (34.8) | 32,191 (33.2) | 47,735 (33.8) | 53,702 (34.2) | 64,278 (35.1) | 67,140 (35.4) | 61,108 (35.7) | 55,655 (35.4) | 36,586 (34.9) |
| 15–20 | 315,397 (26.3) | 26,776 (27.6) | 38,459 (27.2) | 41,922 (26.7) | 46,997 (25.6) | 48,567 (25.6) | 43,639 (25.5) | 41,093 (26.1) | 27,944 (26.7) |
| >20 | 188,000 (15.6) | 17,272 (17.8) | 23,688 (16.8) | 25,859 (16.5) | 28,222 (15.4) | 28,160 (14.8) | 24,591 (14.4) | 24,077 (15.3) | 16,131 (15.4) |
| Dependence category – no. (%) |  |  |  |  |  |  |  |  |  |
| Without assistance | 977,438 (79.0) | 80,193 (79.0) | 117,155 (79.7) | 127,989 (78.7) | 149,462 (79.3) | 154,485 (79.2) | 140,772 (80.2) | 126,889 (79.1) | 80,493 (75.4) |
| Some assistance | 246,414 (19.9) | 20,576 (20.3) | 28,854 (19.6) | 33,516 (20.6) | 37,686 (20.0) | 38,974 (20.0) | 33,258 (19.0) | 31,001 (19.3) | 22,549 (21.1) |
| Total assistance | 13,614 (1.1) | 716 (0.7) | 1,031 (0.7) | 1,140 (0.7) | 1,438 (0.8) | 1,603 (0.8) | 1,451 (0.8) | 2,593 (1.6) | 3,642 (3.4) |
| Deprivation index – no. (%) |  |  |  |  |  |  |  |  |  |
| 1 (least deprived) | 204,989 (16.7) | 16,740 (16.5) | 24,726 (16.8) | 26,915 (16.6) | 31,376 (16.8) | 32,410 (16.8) | 28,970 (16.7) | 26,563 (16.8) | 17,289 (16.5) |
| 2 | 226,623 (18.5) | 18,869 (18.6) | 26,889 (18.3) | 29,610 (18.3) | 34,966 (18.7) | 35,901 (18.6) | 31,999 (18.5) | 28,947 (18.3) | 19,442 (18.6) |
| 3 | 244,996 (20.0) | 20,223 (19.9) | 29,737 (20.3) | 31,941 (19.7) | 37,351 (20.0) | 38,551 (20.0) | 34,730 (20.1) | 31,528 (20.0) | 20,935 (20.0) |
| 4 | 262,925 (21.4) | 21,559 (21.2) | 31,156 (21.2) | 34,634 (21.4) | 40,039 (21.4) | 41,442 (21.5) | 37,355 (21.6) | 34,059 (21.6) | 22,681 (21.7) |
| 5 (most deprived) | 286,673 (23.4) | 24,271 (23.9) | 34,260 (23.3) | 38,766 (23.9) | 43,476 (23.2) | 44,720 (23.2) | 40,019 (23.1) | 36,890 (23.4) | 24,271 (23.2) |
| **Centre-level factors** |  |  |  |  |  |  |  |  |  |
| Type of hospital |  |  |  |  |  |  |  |  |  |
| Non-university | 552,657 (44.4) | 52,104 (50.9) | 66,175 (44.7) | 72,916 (44.6) | 81,415 (43.0) | 86,388 (44.1) | 76,912 (43.7) | 69,188 (42.8) | 47,559 (44.1) |
| University | 503,556 (40.4) | 32,933 (32.2) | 57,188 (38.6) | 64,490 (39.4) | 79,392 (41.9) | 81,117 (41.4) | 73,004 (41.4) | 70,734 (43.7) | 44,698 (41.5) |
| University affiliated | 188,866 (15.2) | 17,349 (16.9) | 24,694 (16.7) | 26,177 (16.0) | 28,737 (15.2) | 28,342 (14.5) | 26,235 (14.9) | 21,805 (13.5) | 15,527 (14.4) |
| **Outcome – no. (%)** |  |  |  |  |  |  |  |  |  |
| In-hospital mortality | 188,844 (15.2) | 18,282 (17.9) | 23,333 (15.8) | 25,443 (15.6) | 27,510 (14.5) | 27,452 (14.0) | 25,218 (14.4) | 25,535 (15.9) | 16,071 (15.0) |
| WLST decision | 77,726 (6.2) | 6,347 (6.2) | 8,738 (5.9) | 9,750 (6.0) | 11,225 (5.9) | 11,774 (6.0) | 10,969 (6.2) | 11,393 (7.0) | 7,530 (7.0) |

*Abbreviations:* APACHE= Acute Physiology and Chronic Health Evaluation; IQR=Interquartile Range; SD=Standard Deviation; TBI=Traumatic Brain Injury; WLST=Withdrawal of Life-Sustaining Therapy. *Table legend:* *****Derived from the patient’s usual residential postcode according to the Index of Multiple Deprivation 2010 for England, Welsh Index of Multiple Deprivation 2008 or Northern Ireland Multiple Deprivation Measure 2010.

## Table S7: Univariable and multivariable analysis representing association between main variables and hospital mortality

| **Covariates** | **No. of available data** | **Unadjusted OR** | **Adjusted OR** |
| --- | --- | --- | --- |
| **Year of admission** (main independent covariate) | 45,684 |  |  |
| 2009–2010 |  | Reference | Reference |
| 2011–2012 |  | 1.00 (0.91 to 1.10) | 1.01 (0.85 to 1.21) |
| 2013–2014 |  | 1.09 (1.00 to 1.20) | 1.03 (0.87 to 1.22) |
| 2015–2016 |  | 1.09 (1.00 to 1.19) | 1.09 (0.92 to 1.28) |
| 2017–2018 |  | 1.19 (1.10 to 1.30) | 1.11 (0.94 to 1.31) |
| 2019–2020 |  | 1.33 (1.22 to 1.45) | 1.41 (1.20 to 1.67) |
| 2021–2022 |  | 1.44 (1.31 to 1.57) | 1.48 (1.25 to 1.75) |
| 2023–2024 |  | 1.57 (1.42 to 1.73) | 1.63 (1.36 to 1.95) |
| Age, years – RCS (22, 51, 77) | 45,684 |  |  |
| Spline age–1 |  | 1.01 (1.01 to 1.02) | 1.19 (1.09 to 1.3) |
| Spline age–2 |  | 1.02 (1.01 to 1.02) | 1.92 (1.74 to 2.1) |
| Sex | 45,684 |  |  |
| Male |  | Reference | Reference |
| Female |  | 1.35 (1.29 to 1.41) | 0.94 (0.88 to 1.01) |
| Deprivation index | 44,065 |  |  |
| 1 (least deprived) |  | Reference | Reference |
| 2 |  | 1.00 (0.92 to 1.07) | 1.06 (0.95 to 1.18) |
| 3 |  | 0.90 (0.84 to 0.96) | 0.95 (0.86 to 1.06) |
| 4 |  | 0.79 (0.74 to 0.85) | 0.88 (0.79 to 0.98) |
| 5 (most deprived) |  | 0.75 (0.70 to 0.80) | 0.95 (0.86 to 1.06) |
| Type of ICU | 45,684 |  |  |
| General ICU |  | Reference | Reference |
| General ICU with neurosurgery |  | 0.71 (0.68 to 0.75) | 0.81 (0.69 to 0.95) |
| Specialist neuroscience ICU |  | 0.63 (0.60 to 0.67) | 0.80 (0.70 to 0.94) |
| Surgery | 45,684 |  |  |
| No |  | Reference | Reference |
| Yes |  | 0.69 (0.66 to 0.73) | 0.76 (0.70 to 0.82) |
| Pupils reactivity | 42,725 |  |  |
| Both reactive |  | Reference | Reference |
| One reactive |  | 2.70 (2.43 to 3.00) | 2.21 (1.93 to 2.53) |
| Both unreactive |  | 22.53 (21.01 to 24.18) | 11.39 (10.39 to 12.50) |
| GCS – motor component | 44,632 |  |  |
| Localise or obeys |  | Reference | Reference |
| Normal flexion |  | 3.99 (3.51 to 4.54) | 2.53 (2.13 to 3.01) |
| Abnormal flexion |  | 8.14 (6.83 to 9.69) | 5.35 (4.16 to 6.88) |
| Extension |  | 16.92 (14.32 to 20.00) | 8.55 (6.71 to 10.90) |
| None |  | 40.17 (36.77 to 43.92) | 16.68 (14.66 to 18.97) |
| Untestable |  | 5.38 (5.04 to 5.75) | 4.55 (4.14 to 5.00) |
| Lowest SAP, mmHg – RCS (70, 94, 105, 130) | 45,298 |  |  |
| Spline SAP–1 |  | 0.94 (0.93 to 0.94) | 0.51 (0.45 to 0.56) |
| Spline SAP–2 |  | 1.07 (1.05 to 1.08) | 3.15 (2.31 to 4.30) |
| Spline SAP–3 |  | 0.91 (0.86 to 0.95) | 0.04 (0.01 to 0.20) |
| Lowest PaO_2_, mmHg – RCS (62, 80, 116) | 41,265 |  |  |
| Spline PaO_2_–1 |  | 0.98 (0.97 to 0.98) | 0.56 (0.48 to 0.64) |
| Spline PaO_2_–2 |  | 1.02 (1.01 to 1.02) | 1.68 (1.40 to 2.02) |
| PaCO_2_, mmHg – RCS (33.0, 39.4, 48.8) | 41,262 |  |  |
| Spline PaCO_2_–1 |  | 0.92 (0.91 to 0.93) | 0.44 (0.39 to 0.51) |
| Spline PaCO_2_–2 |  | 1.07 (1.06 to 1.08) | 2.03 (1.80 to 2.30) |
| Lowest glucose level, mmol/l – RCS (4.5, 6.1, 8.4) | 41,505 |  |  |
| Spline low glucose–1 |  | 1.11 (1.07 to 1.14) | 0.90 (0.81 to 0.99) |
| Spline low glucose–2 |  | 1.18 (1.13 to 1.23) | 1.28 (1.14 to 1.44) |
| Highest glucose level, mmol/l – RCS (6.3, 8.8, 13.2) | 37,125 |  |  |
| Spline high glucose–1 |  | 1.66 (1.62 to 1.7) | 2.75 (2.41 to 3.13) |
| Spline high glucose–2 |  | 0.68 (0.66 to 0.7) | 0.42 (0.36 to 0.49) |
| Lowest haemoglobin, g/dl – RCS (8.2, 11.3, 14) | 43,343 |  |  |
| Spline haemoglobin–1 |  | 0.78 (0.76 to 0.79) | 0.62 (0.58 to 0.66) |
| Spline haemoglobin–2 |  | 1.26 (1.2 to 1.3) | 1.72 (1.59 to 1.87) |

*Abbreviations:* ICU= Intensive Care Unit; RCS=Restricted Cubic Spline; SAP=Systolic Arterial Pressure

## Figure S1: Adjusted Odds Ratio (aOR) and 95% confidence intervals for continuous variables from the regression model analysing hospital mortality.


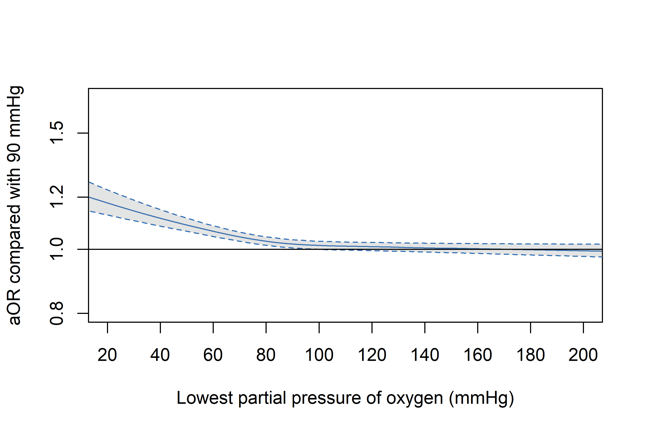

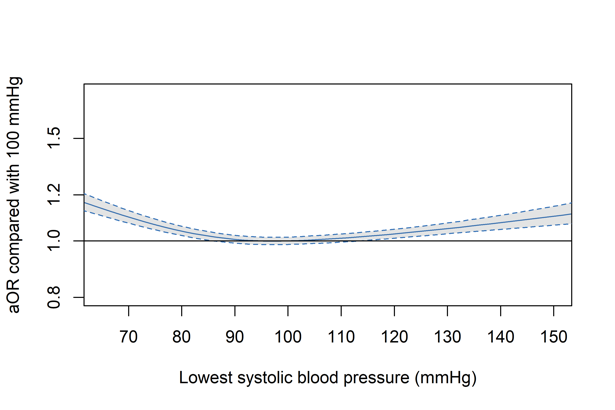

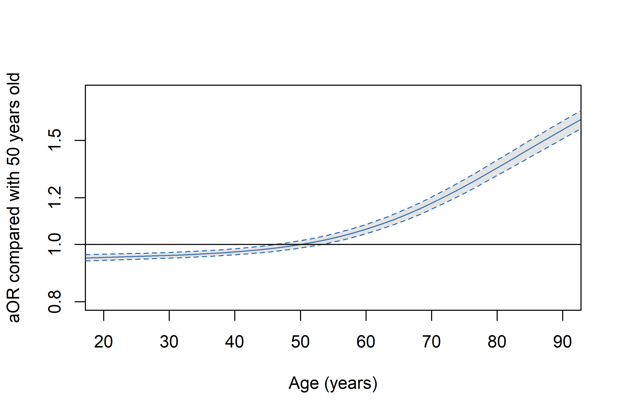
Panel A: Age Panel B: Lowest SAP Panel C: Lowest PaO_2_


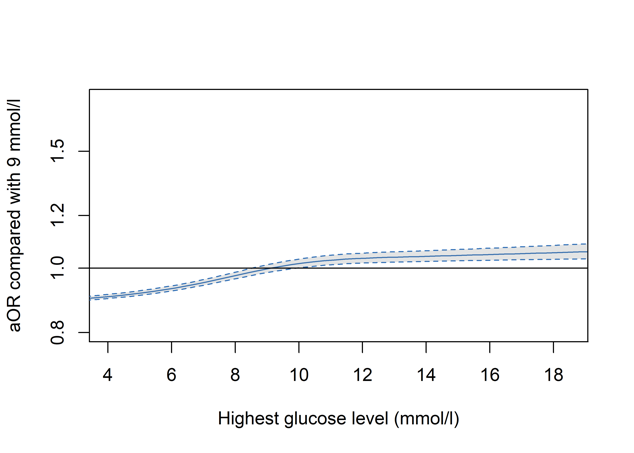

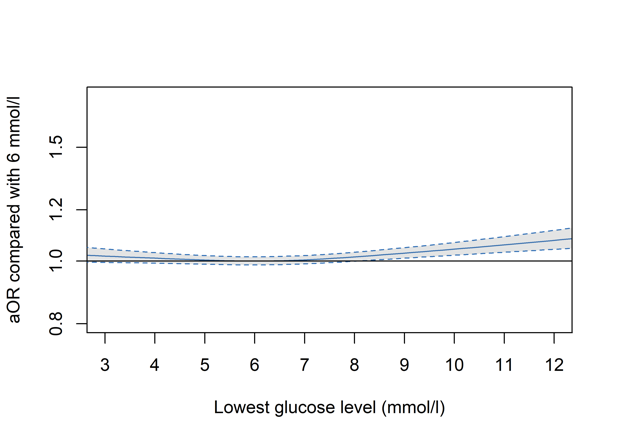

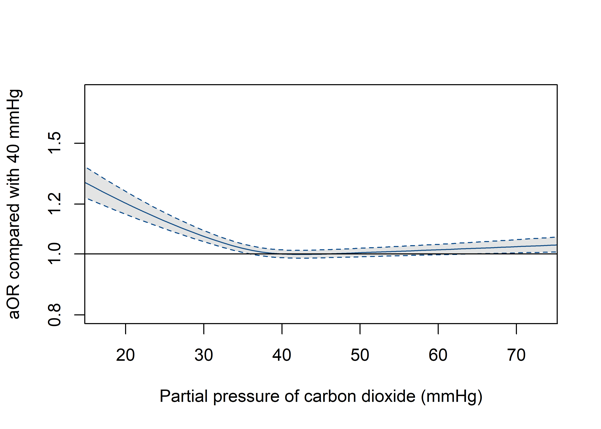
Panel D: PaCO_2_ Panel E: Lowest glucose level Panel F: Highest glucose level


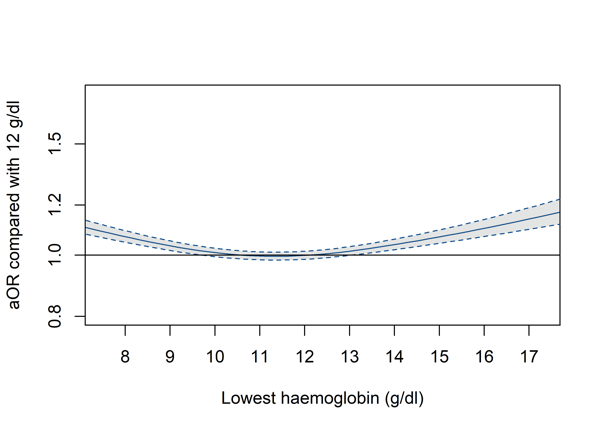
Panel G: Lowest haemoglobin

*Footnote:* Adjusted ORs are represented on a logarithmic scale.

## Figure S2: Final model performances for hospital mortality.


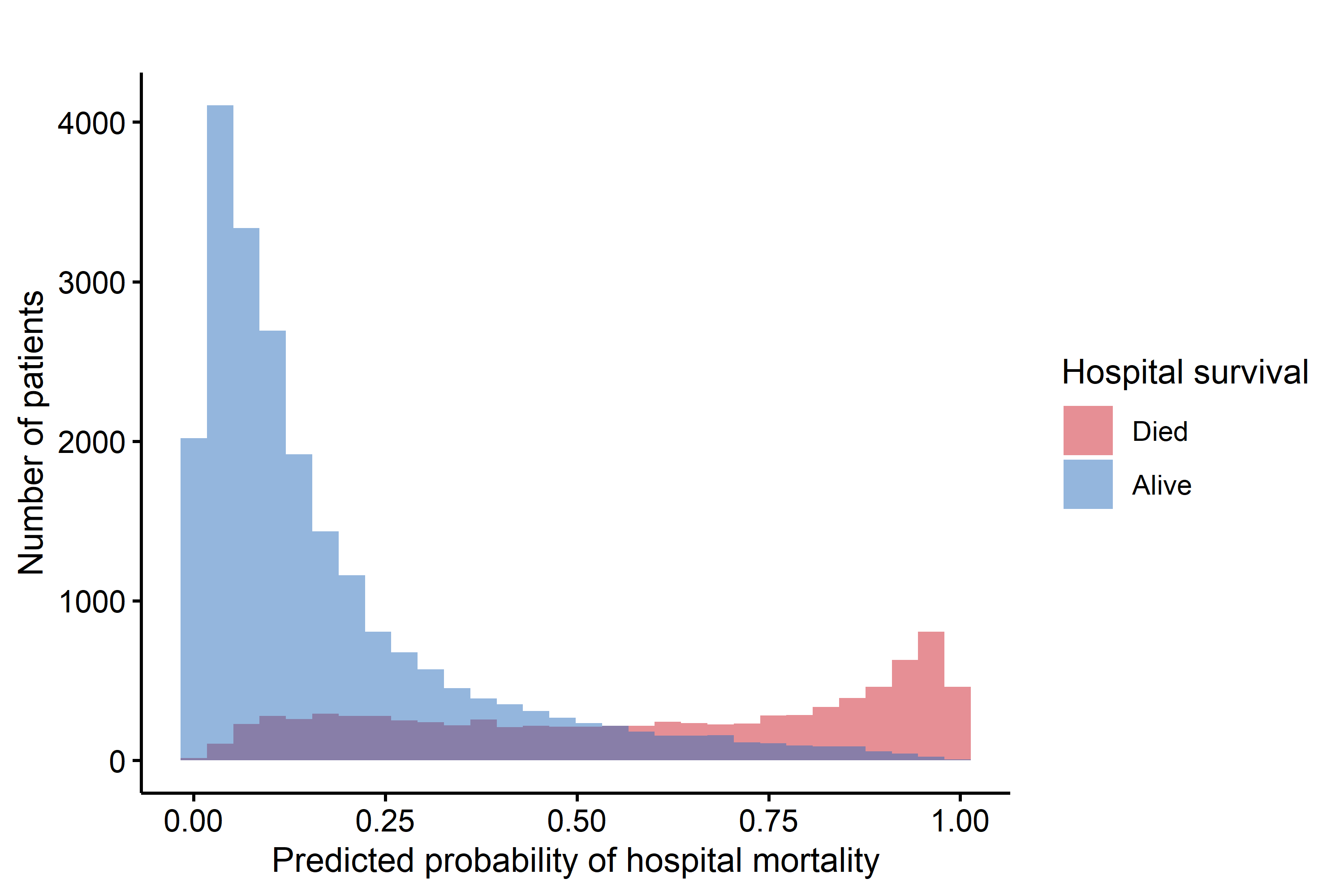
Panel A: Number of patients by hospital survival status and predicted probability of hospital mortality

Panel B: Calibration plot
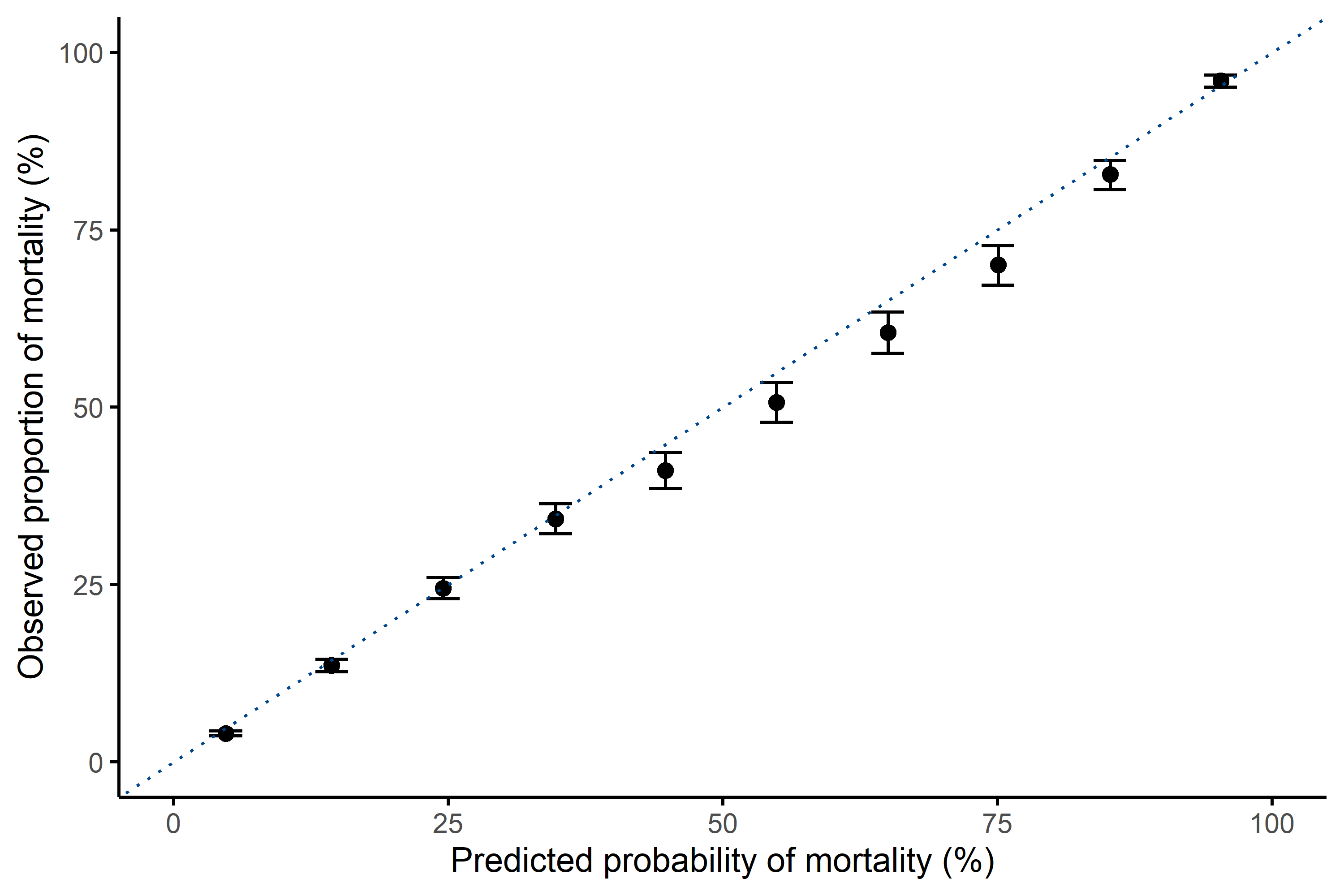


## Table S8: Sensitivity analysis for hospital mortality on imputed dataset

| **Covariates** | **Adjusted OR** |
| --- | --- |
| **Year of admission** (main independent covariate) |  |
| 2009–2010 | Reference |
| 2011–2012 | 1.03 (0.91 to 1.17) |
| 2013–2014 | 1.09 (0.96 to 1.24) |
| 2015–2016 | 1.17 (1.04 to 1.32) |
| 2017–2018 | 1.17 (1.03 to 1.32) |
| 2019–2020 | 1.38 (1.22 to 1.57) |
| 2021–2022 | 1.49 (1.32 to 1.69) |
| 2023–2024 | 1.51 (1.32 to 1.73) |
| Age, years – RCS (22, 51, 77) |  |
| Spline age–1 | 1.24 (1.15 to 1.33) |
| Spline age–2 | 1.93 (1.77 to 2.10) |
| Sex |  |
| Male | Reference |
| Female | 0.93 (0.88 to 0.99) |
| Deprivation index |  |
| 1 (least deprived) | Reference |
| 2 | 1.04 (0.95 to 1.14) |
| 3 | 0.98 (0.90 to 1.08) |
| 4 | 0.93 (0.85 to 1.02) |
| 5 (most deprived) | 0.98 (0.90 to 1.07) |
| Type of ICU |  |
| General ICU | Reference |
| General ICU with neurosurgery | 0.88 (0.76 to 1.03) |
| Specialist neuroscience ICU | 0.79 (0.69 to 0.91) |
| Surgery |  |
| No | Reference |
| Yes | 0.78 (0.73 to 0.84) |
| Pupils reactivity |  |
| Both reactive | Reference |
| One reactive | 2.22 (1.97 to 2.51) |
| Both unreactive | 9.62 (8.95 to 10.34) |
| GCS – motor component |  |
| Localise or obeys | Reference |
| Normal flexion | 2.34 (2.02 to 2.70) |
| Abnormal flexion | 4.70 (3.83 to 5.77) |
| Extension | 8.49 (6.96 to 10.34) |
| None | 16.43 (14.84 to 18.19) |
| Untestable | 4.54 (4.22 to 4.89) |
| Lowest SAP, mmHg – RCS (70, 94, 105, 130) |  |
| Spline SAP–1 | 0.57 (0.5 to 0.6) |
| Spline SAP–2 | 1.75 (1.39 to 2.20) |
| Spline SAP–3 | 0.65 (0.22 to 1.95) |
| Lowest PaO_2_, mmHg – RCS (62, 80, 116) |  |
| Spline PaO_2_–1 | 0.55 (0.48 to 0.64) |
| Spline PaO_2_–2 | 1.70 (1.47 to 1.96) |
| PaCO_2_, mmHg – RCS (33.0, 39.4, 48.8) |  |
| Spline PaCO_2_–1 | 0.49 (0.44 to 0.54) |
| Spline PaCO_2_–2 | 1.89 (1.72 to 2.07) |
| Lowest glucose level, mmol/l – RCS (4.5, 6.1, 8.4) |  |
| Spline low glucose–1 | 2.37 (1.90 to 2.96) |
| Spline low glucose–2 | 0.50 (0.42 to 0.60) |
| Highest glucose level, mmol/l – RCS (6.3, 8.8, 13.2) |  |
| Spline high glucose–1 | 1.49 (1.38 to 1.61) |
| Spline high glucose–2 | 0.90 (0.83 to 0.98) |
| Lowest haemoglobin, g/dl – RCS (8.2, 11.3, 14.0) |  |
| Spline haemoglobin–1 | 0.70 (0.66 to 0.74) |
| Spline haemoglobin–2 | 1.50 (1.40 to 1.60) |

*Abbreviations:* ICU= Intensive Care Unit; RCS=Restricted Cubic Spline; SAP=Systolic Arterial Pressure

## Figure S3: Percentage of hospital mortality and WLST decision for TBI patients and comparator cohorts

Panel A: Hospital mortality

Panel B: WLST decision

## Figure S4: Trends in hospital mortality (panel A, B, C) and WLST decision (panel D, E, F) in matched comparator cohorts.


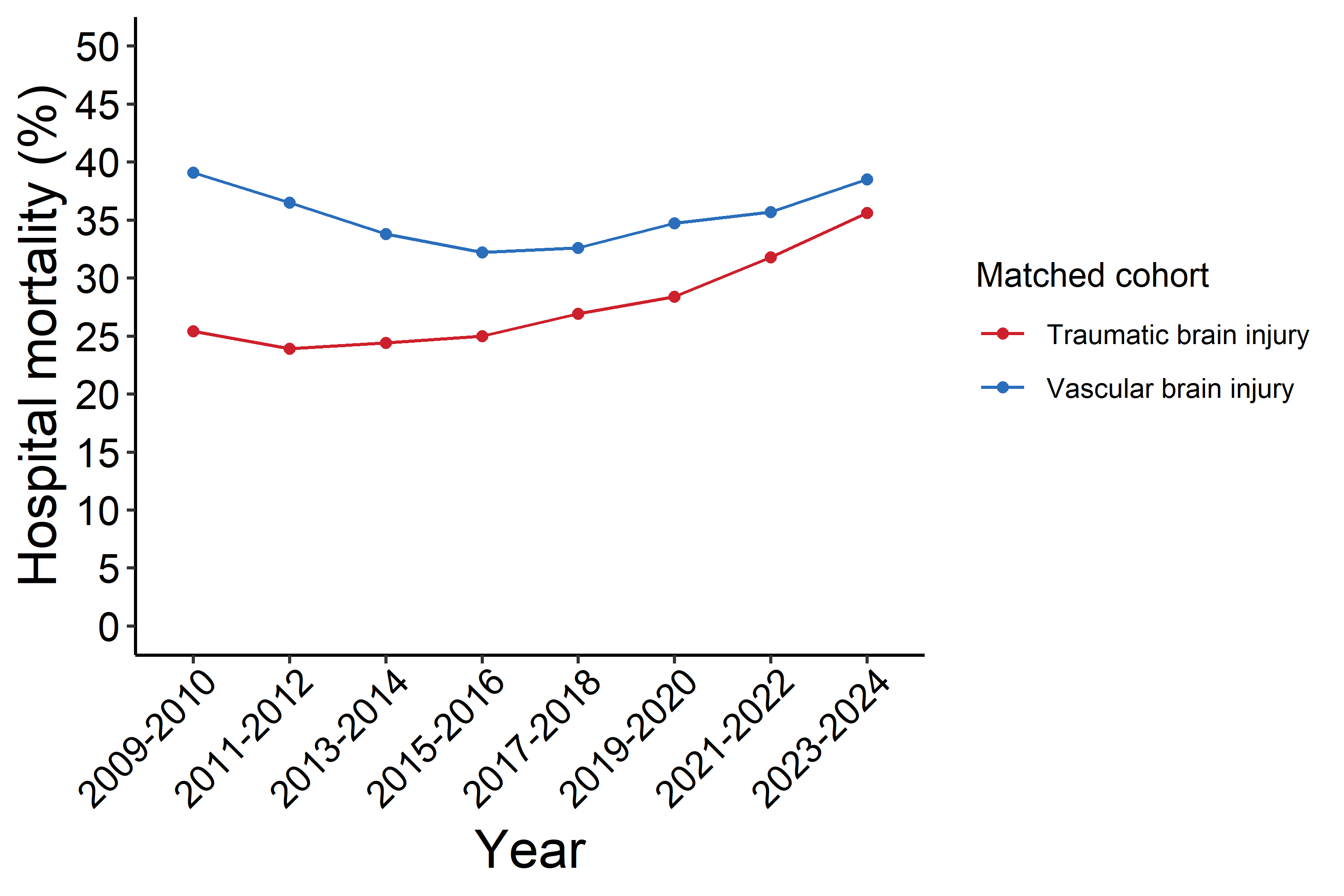

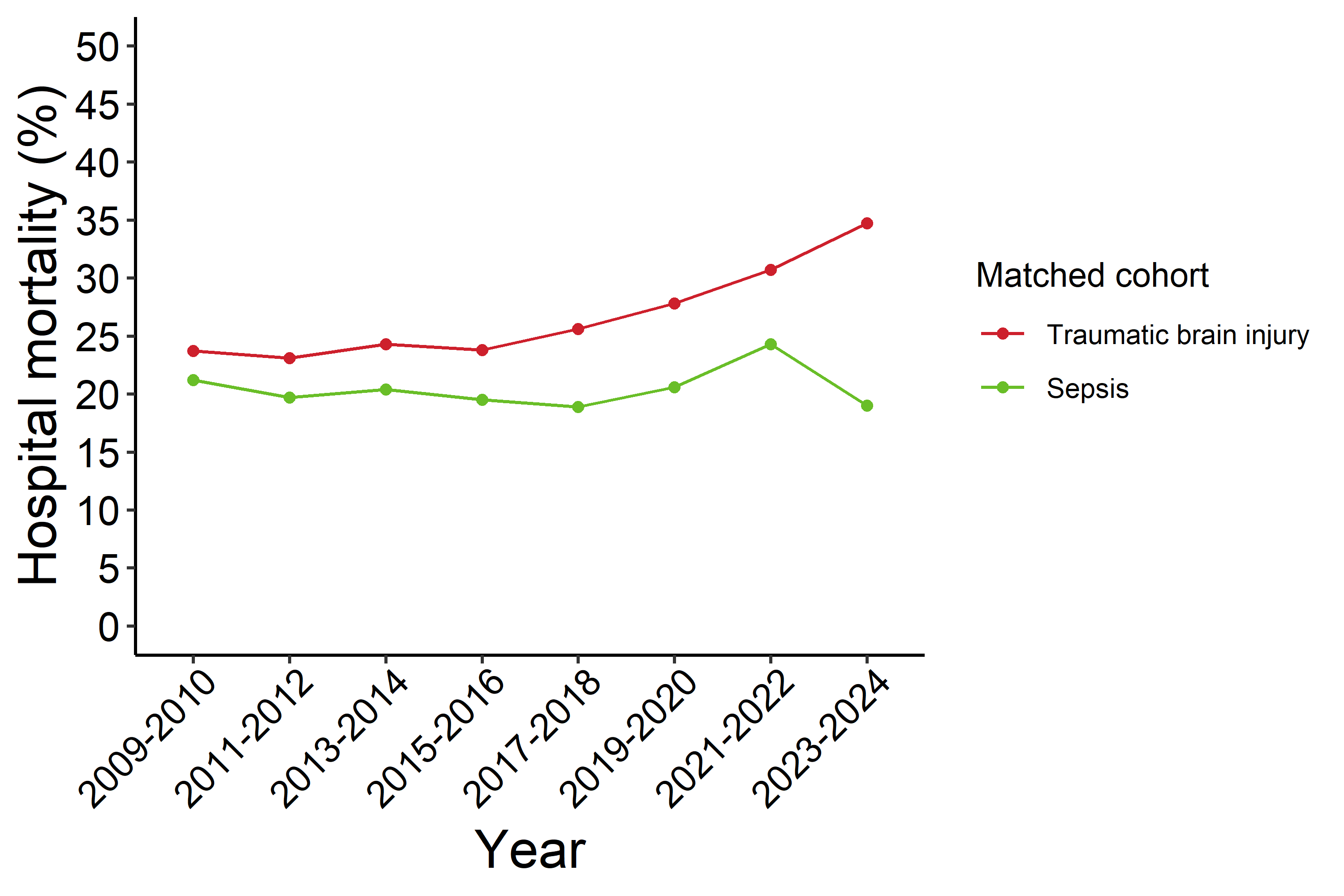

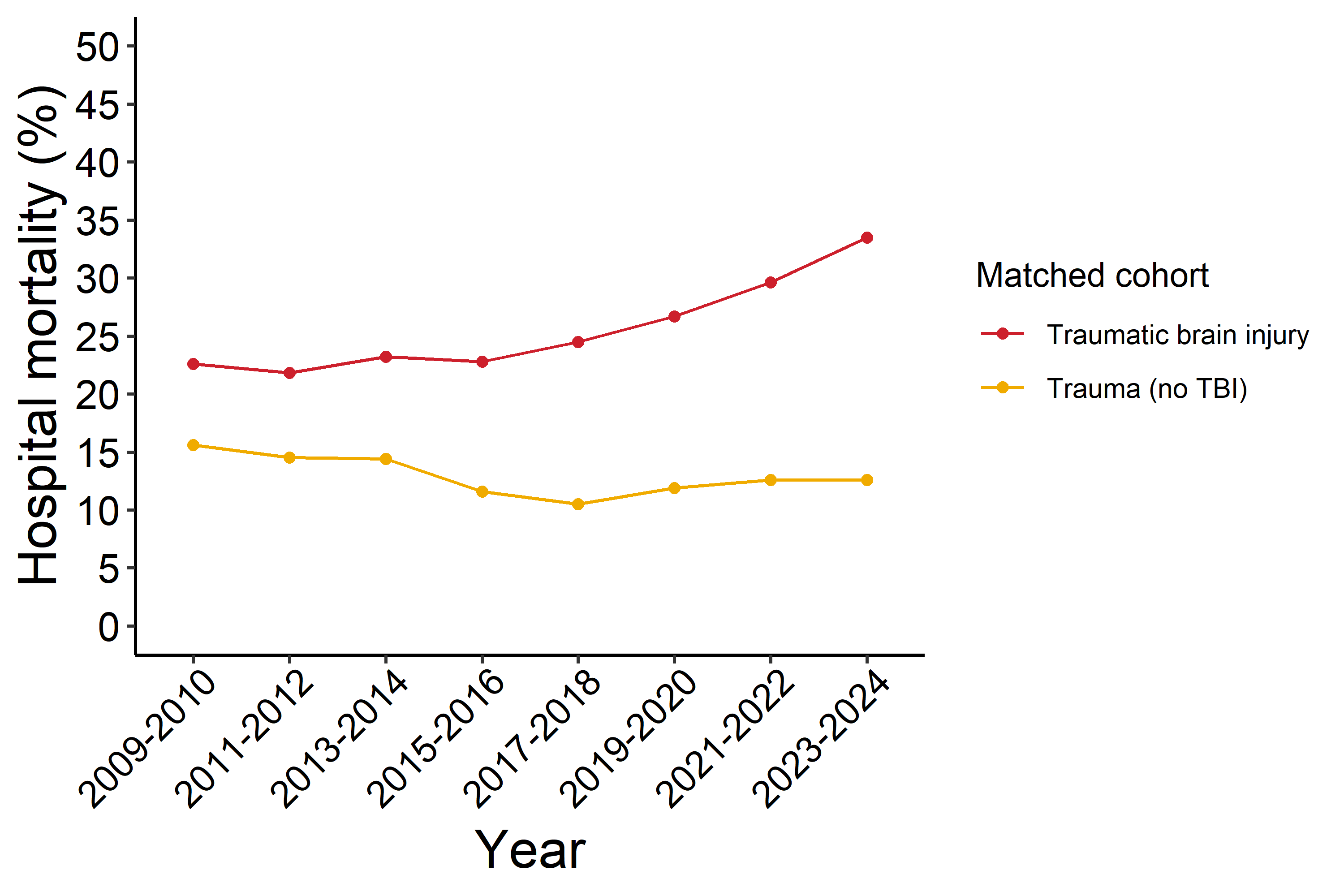
Panel A: TBI matched with trauma patients Panel B: TBI matched with sepsis patients Panel C: TBI matched with vascular brain injury patients


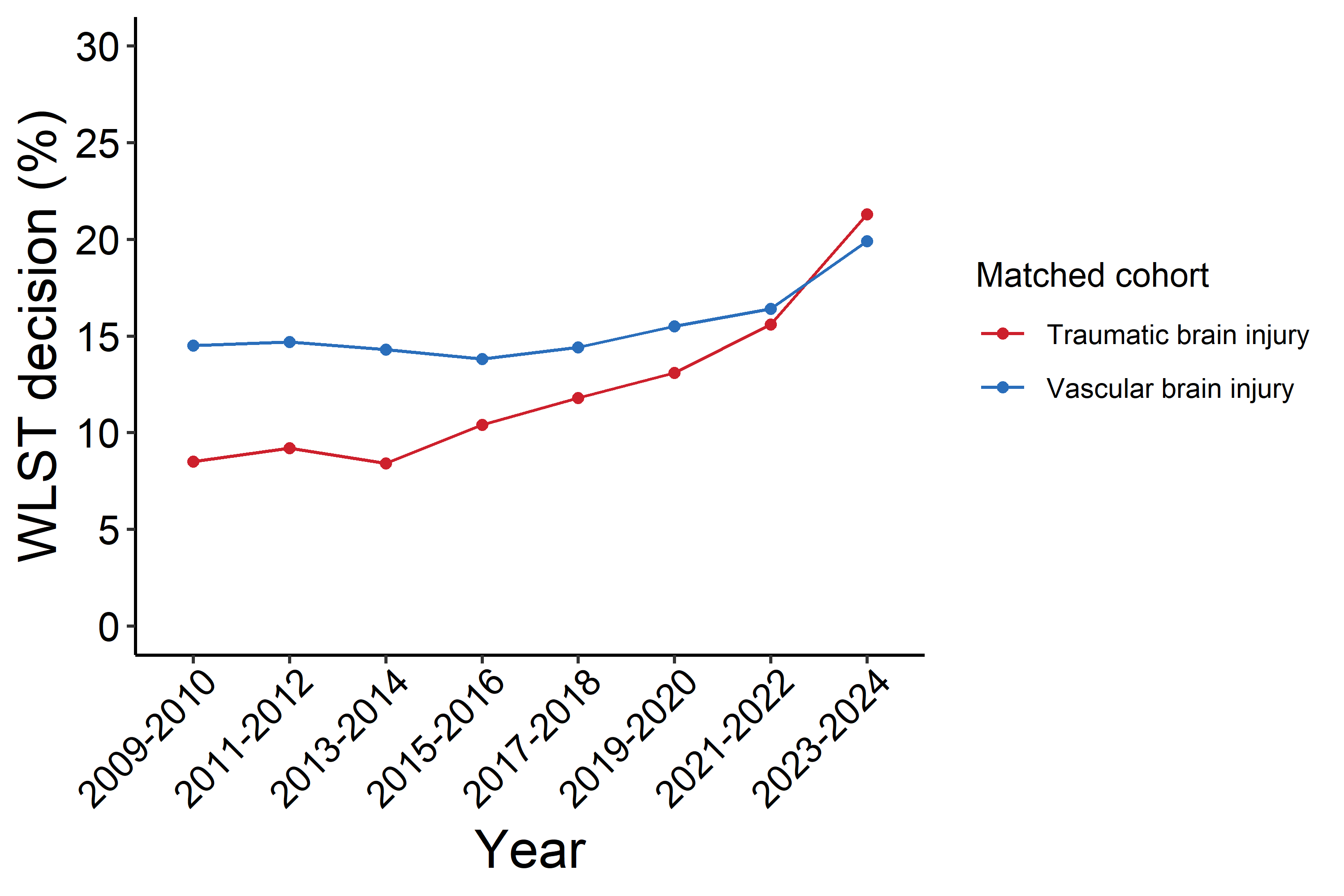

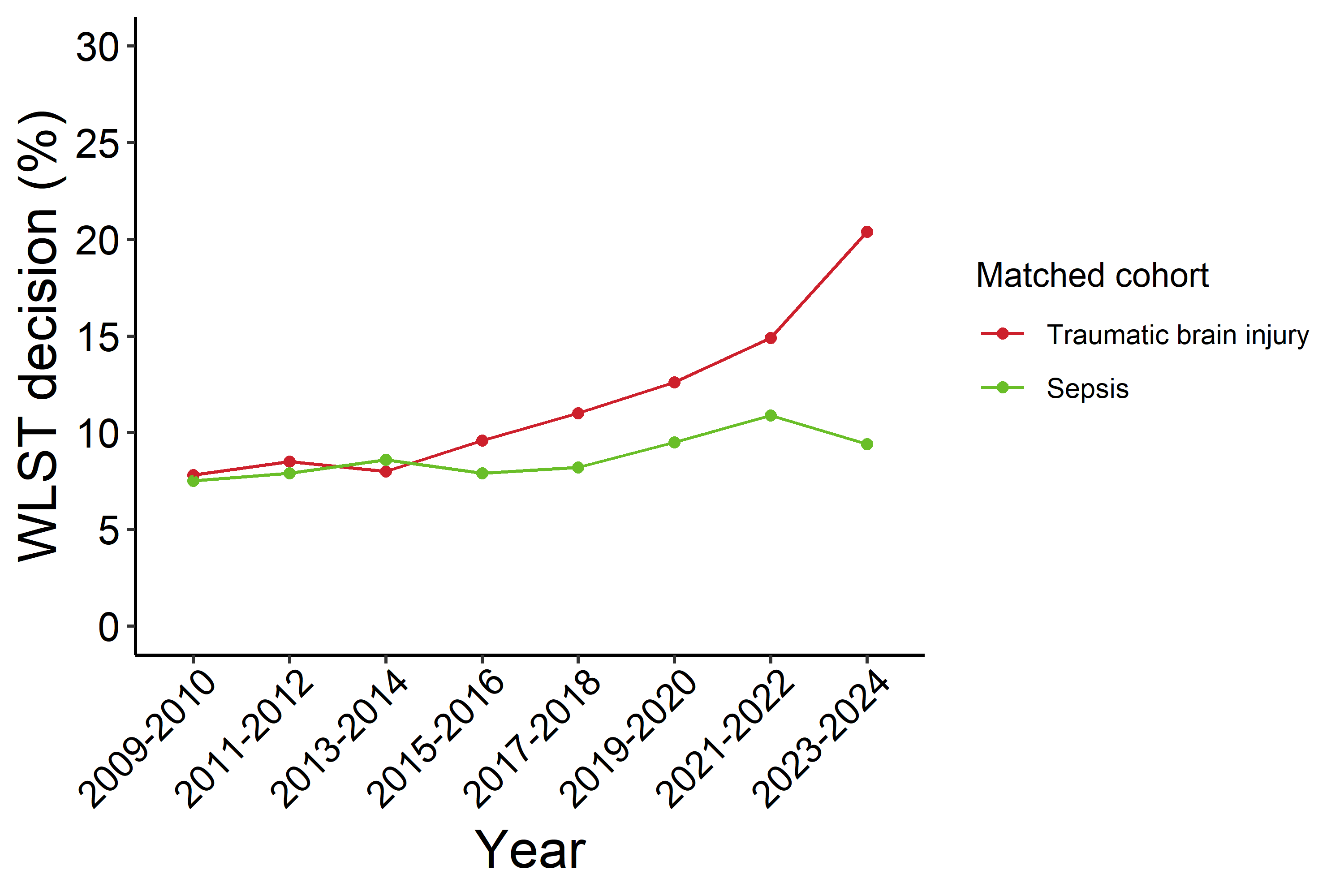

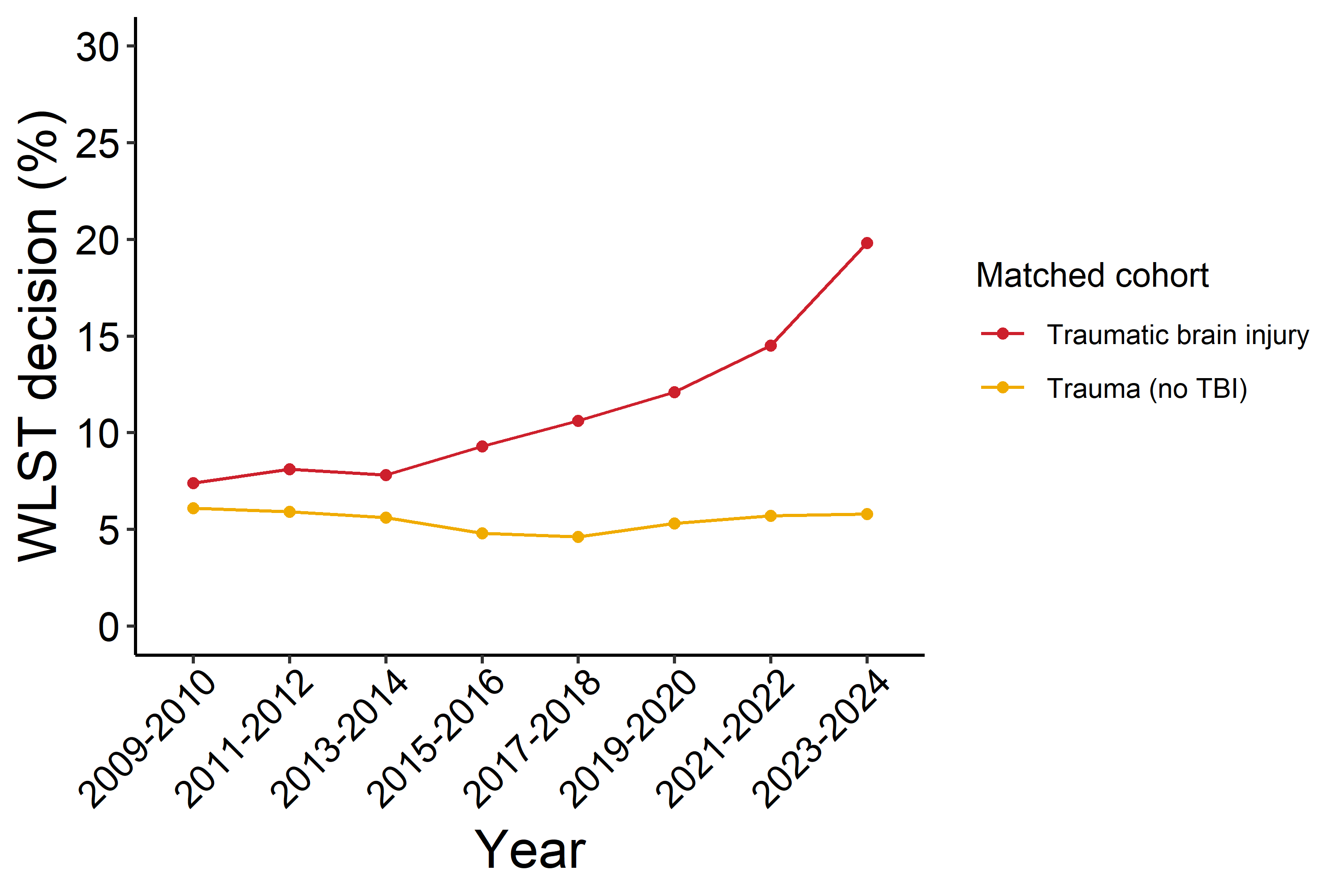
Panel D: TBI matched with trauma patients Panel E: TBI matched with sepsis patients Panel F: TBI matched with vascular brain injury patients

*Footnotes:* During the matching procedure, 41,018 TBI patients were matched with 76,634 trauma patients (panel A and D), 40,129 TBI patients were matched with 107,823 sepsis patients (panel B and E) and 35,054 TBI patients were matched with 60,701 vascular brain injury patients (panel C and F).

## Figure S5: Trends in hospital mortality and WLST decision for TBI patients and other ICU patients (except those included in trauma, sepsis and vascular brain injury comparator cohorts)


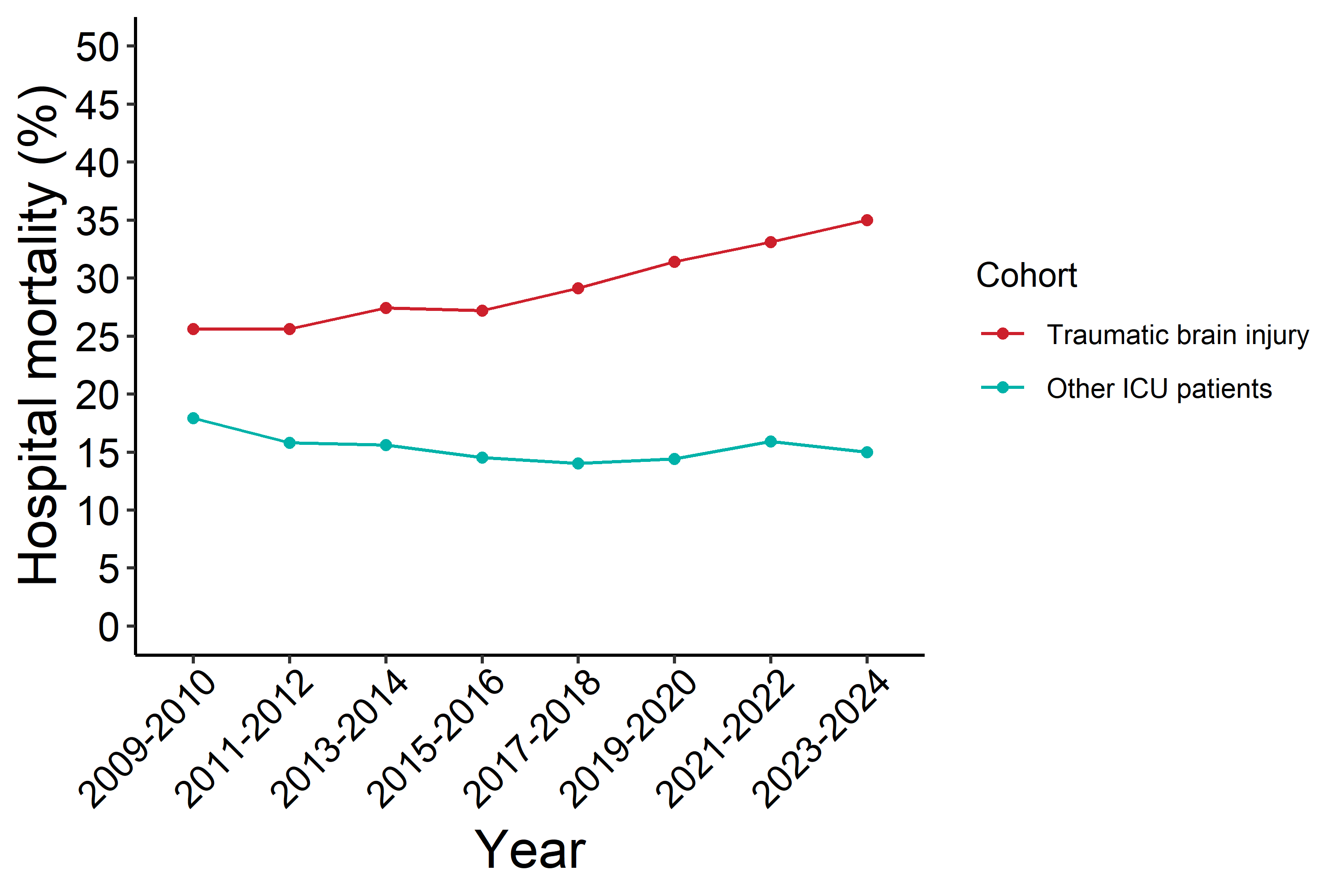
Panel A: Hospital mortality for all TBI patients and all other ICU patients Panel B: WLST decision for all TBI patients and all other ICU patients


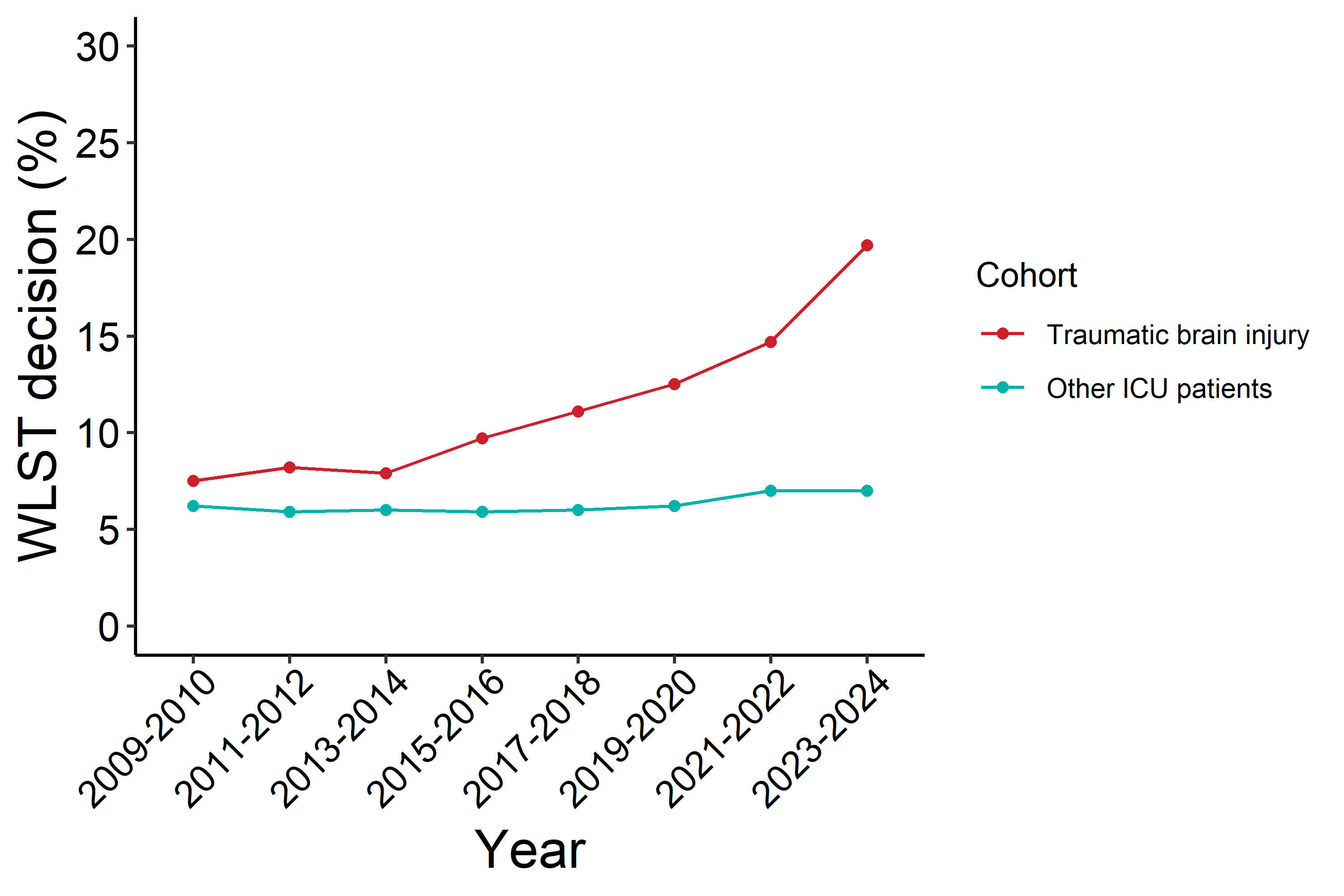


Panel C: Hospital mortality in matched cohorts Panel D: WLST decision in matched cohorts


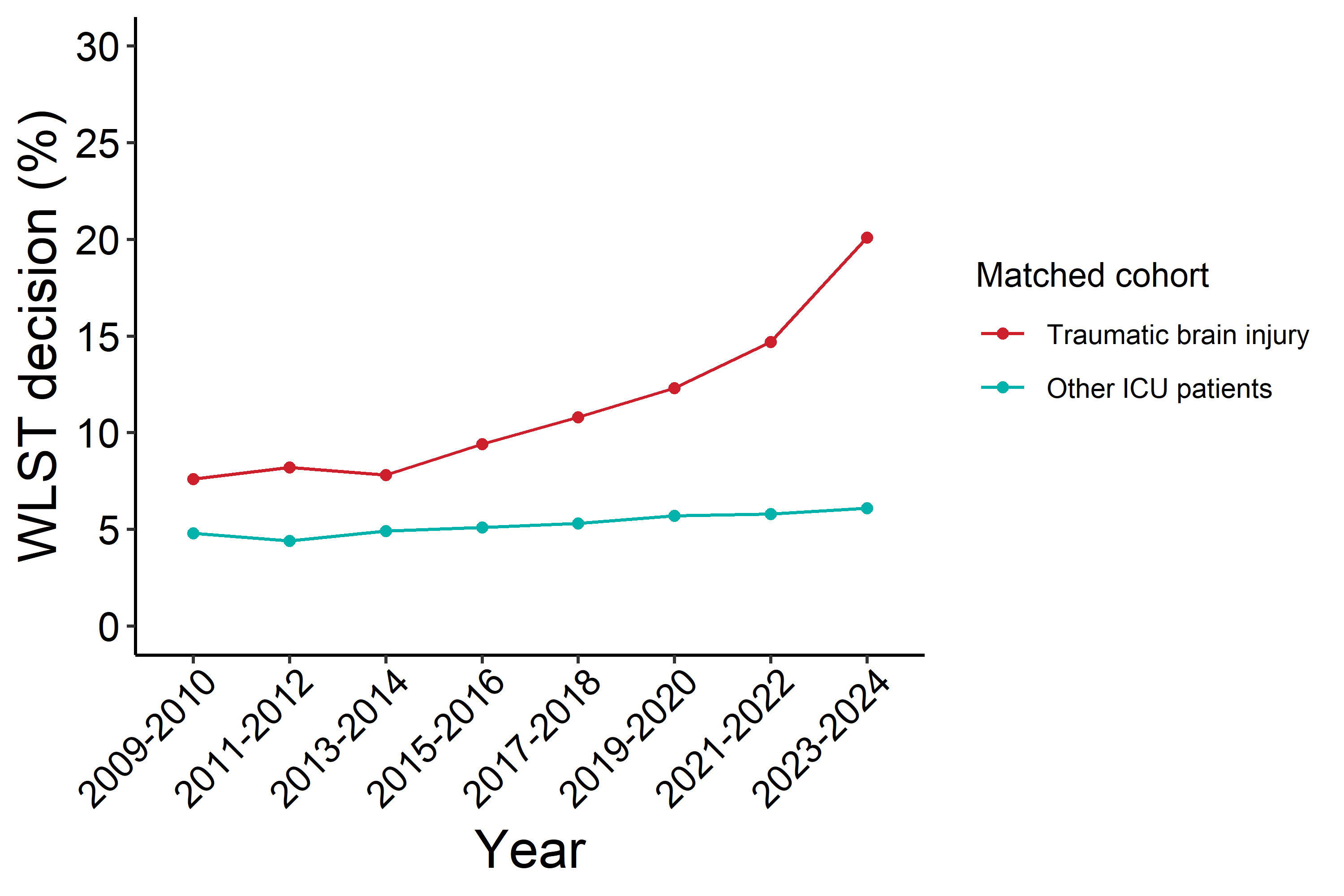

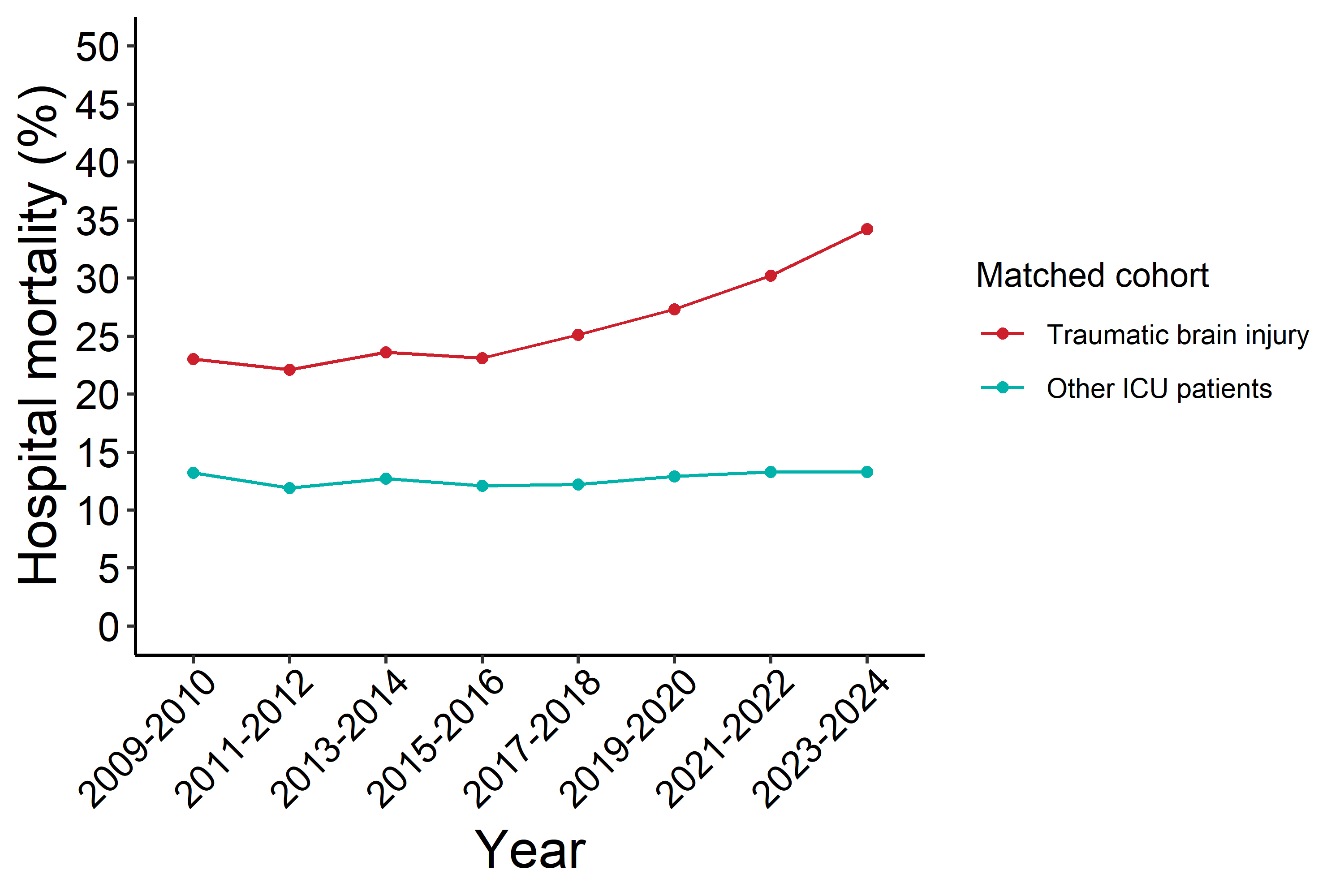


*Footnotes:* During the matching procedure, 41,419 TBI patients were matched with 205,693 other ICU patients (panel C and D)

## Table S9: Post-hoc sensitivity analysis for primary and secondary outcome restricted to centres recruited TBI patients all over the study period

| **Variables** | **April 2009**–**2010** | **2011**–**2012** | **2013**–**2014** | **2015**–**2016** | **2017**–**2018** | **2019**–**2020** | **2021**–**2022** | **2023**–  **March 2024** |
| --- | --- | --- | --- | --- | --- | --- | --- | --- |
|  | N = 3,472 | N = 4,294 | N = 4,376 | N = 5,500 | N = 5,863 | N = 5,326 | N = 5,018 | N = 3,156 |
| **Outcome – no. (%)** |  |  |  |  |  |  |  |  |
| In-hospital mortality | 862 (25·3) | 1,067 (25·2) | 1,175 (27·1) | 1,514 (27·8) | 1,700 (29·4) | 1,678 (32·0) | 1,629 (32·8) | 1,085 (35·3) |
| WLST decision | 254 (7·4) | 319 (7·6) | 357 (8·4) | 556 (10·4) | 660 (11·6) | 656 (12·8) | 685 (14·1) | 631 (20·0) |

## Figure S6: Trends in hospital mortality (panel A) and WLST decision (panel B) each year, before (2019), during (2020-2021) and after (2022-2024) COVID-19 pandemic

Panel A: Percentage of hospital mortality for TBI patients and comparator cohorts


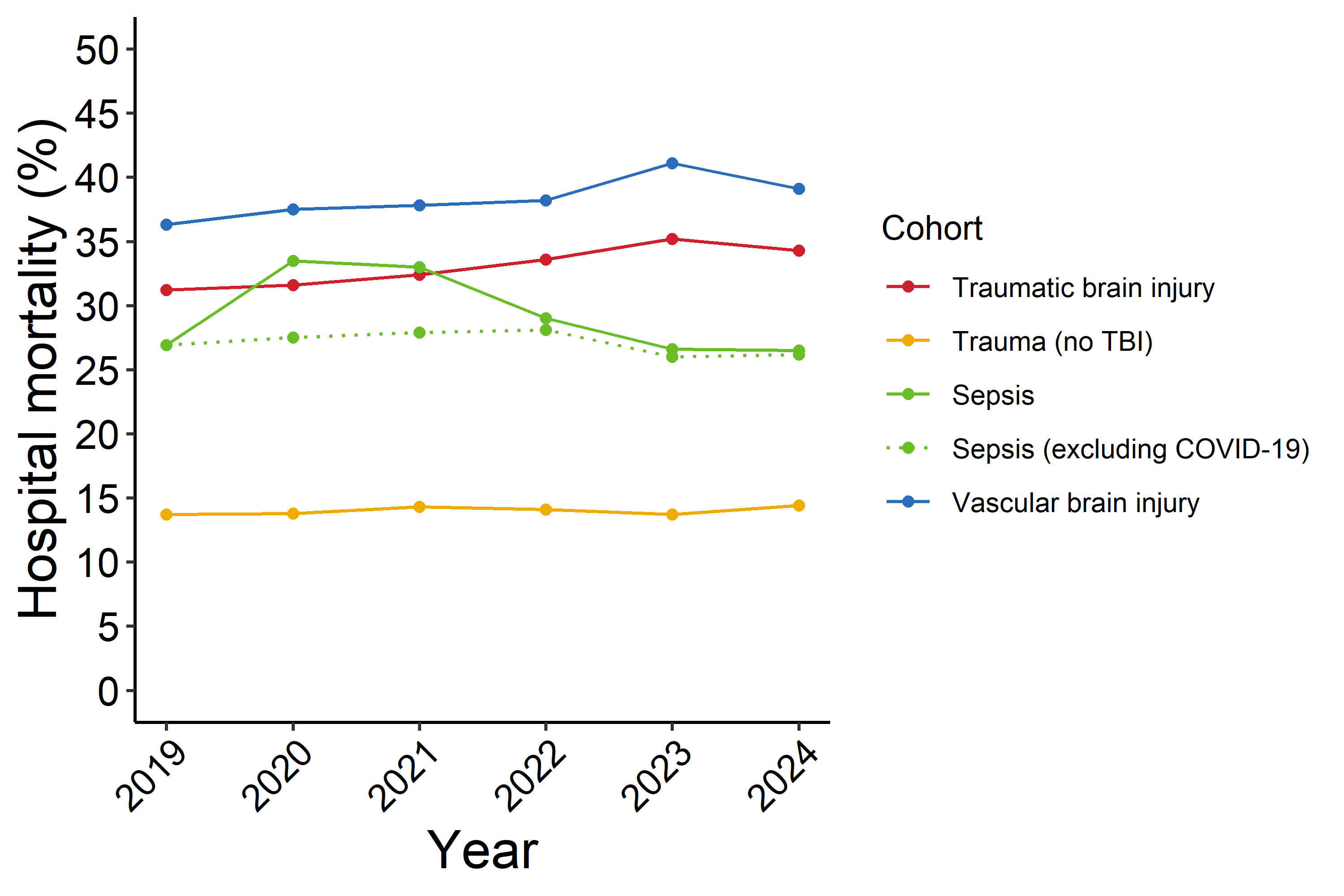


Panel B: Percentage of WLST decision for TBI patients and comparative cohorts


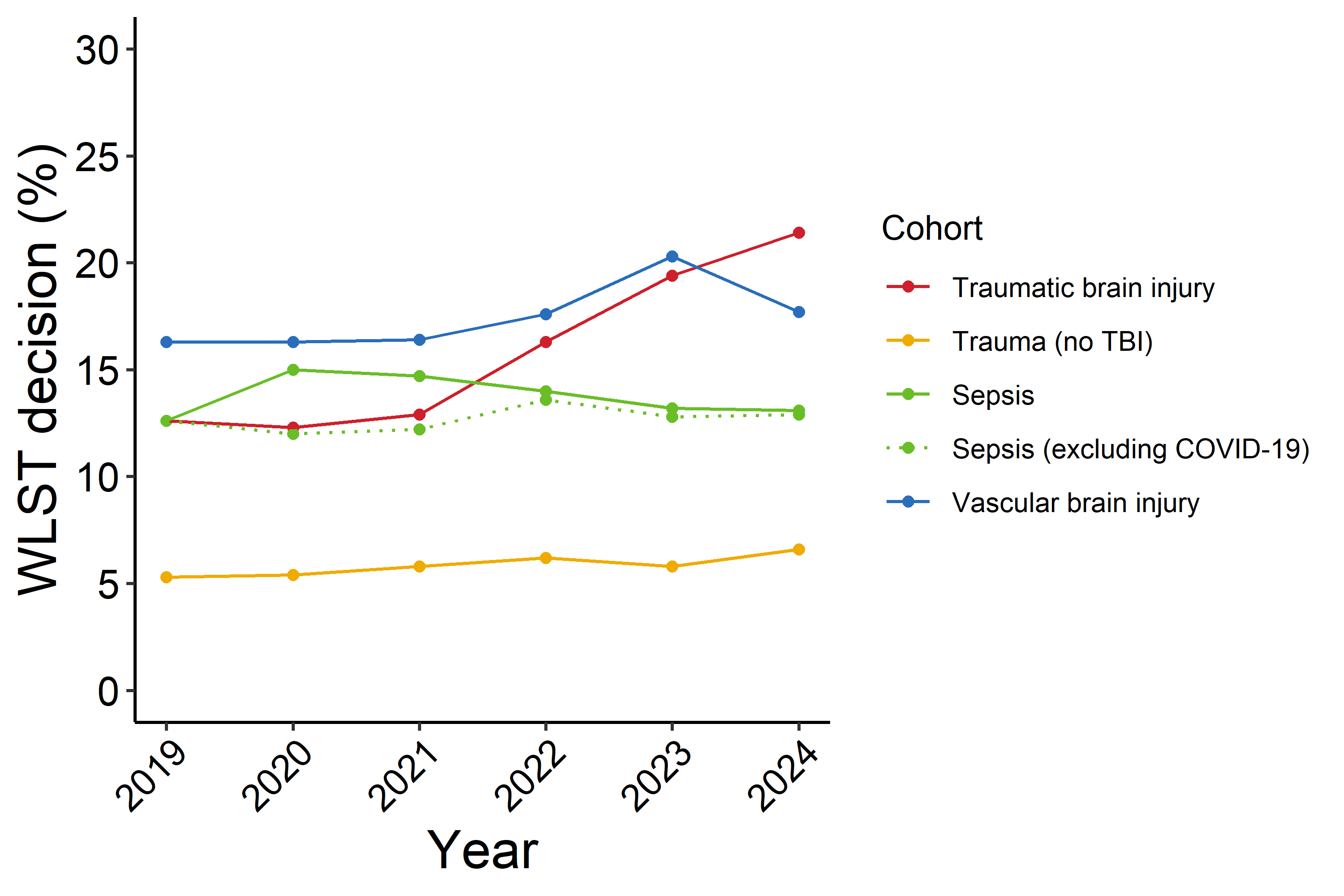


## Table S10: Univariable and multivariable analysis representing association between main variables and WLST

| **Covariates** | **No. of available data** | **Unadjusted OR** | **Adjusted OR** |
| --- | --- | --- | --- |
| **Year of admission** (main independent covariate) | 45,684 |  |  |
| 2009–2010 |  | Reference | Reference |
| 2011–2012 |  | 1.10 (0.94 to 1.28) | 1.06 (0.84 to 1.34) |
| 2013–2014 |  | 1.06 (0.91 to 1.23) | 0.95 (0.75 to 1.20) |
| 2015–2016 |  | 1.33 (1.16 to 1.54) | 1.23 (0.99 to 1.54) |
| 2017–2018 |  | 1.55 (1.35 to 1.78) | 1.30 (1.04 to 1.62) |
| 2019–2020 |  | 1.77 (1.54 to 2.03) | 1.59 (1.28 to 1.98) |
| 2021–2022 |  | 2.14 (1.87 to 2.46) | 1.83 (1.47 to 2.28) |
| 2023–2024 |  | 3.04 (2.64 to 3.50) | 2.80 (2.24 to 3.51) |
| Age, years – RCS (22, 51, 77) | 45,684 |  |  |
| Spline age–1 |  | 1.02 (1.01 to 1.03) | 1.49 (1.32 to 1.68) |
| Spline age–2 |  | 1.01 (1.00 to 1.01) | 1.33 (1.17 to 1.51) |
| Dependence at home | 44,641 |  |  |
| Living without assistance |  | Reference | Reference |
| Some assistance |  | 1.72 (1.56 to 1.88) | 1.20 (1.05 to 1.37) |
| Total assistance |  | 2.00 (1.42 to 2.76) | 1.40 (0.82 to 2.41) |
| Deprivation index | 44,065 |  |  |
| 1 (least deprived) |  | Reference | Reference |
| 2 |  | 1.01 (0.91 to 1.11) | 1.13 (0.99 to 1.29) |
| 3 |  | 0.90 (0.81 to 0.99) | 0.99 (0.87 to 1.13) |
| 4 |  | 0.79 (0.72 to 0.87) | 0.93 (0.81 to 1.06) |
| 5 (most deprived) |  | 0.74 (0.67 to 0.82) | 0.95 (0.84 to 1.09) |
| Type of ICU | 45,684 |  |  |
| ICU without neurosurgery |  | Reference | Reference |
| ICU with neurosurgery |  | 0.82 (0.77 to 0.88) | 1.03 (0.83 to 1.29) |
| Neuroscience centre |  | 0.91 (0.84 to 0.98) | 0.93 (0.76 to 1.14) |
| Surgery | 45,684 |  |  |
| No |  | Reference | Reference |
| Yes |  | 0.82 (0.76 to 0.88) | 0.94 (0.86 to 1.04) |
| Pupils reactivity | 42,725 |  |  |
| Both reactive |  | Reference | Reference |
| One reactive |  | 3.12 (2.73 to 3.56) | 2.39 (2.03 to 2.81) |
| Both unreactive |  | 7.25 (6.78 to 7.75) | 3.72 (3.38 to 4.09) |
| GCS – motor component | 44,632 |  |  |
| Localise or obeys |  | Reference | Reference |
| Normal flexion |  | 4.74 (3.78 to 5.91) | 3.28 (2.53 to 4.25) |
| Abnormal flexion |  | 10.93 (8.47 to 14.01) | 6.15 (4.44 to 8.52) |
| Extension |  | 21.36 (17.22 to 26.44) | 9.69 (7.33 to 12.81) |
| None |  | 23.22 (20.32 to 26.62) | 8.68 (7.28 to 10.36) |
| Untestable |  | 8.65 (7.64 to 9.82) | 6.95 (5.95 to 8.11) |
| Lowest SAP, mmHg – RCS (70, 94, 105, 130) | 45,298 |  |  |
| Spline SAP–1 |  | 0.96 (0.95 to 0.97) | 0.78 (0.71 to 0.86) |
| Spline SAP–2 |  | 1.02 (1.01 to 1.04) | 1.83 (1.32 to 2.54) |
| Spline SAP–3 |  | 1.03 (0.97 to 1.10) | 0.17 (0.03 to 0.97) |
| Lowest PaO_2_, mmHg – RCS (62, 80, 116) | 41,265 |  |  |
| Spline PaO_2_–1 |  | 0.99 (0.98 to 1.00) | 0.71 (0.60 to 0.84) |
| Spline PaO_2_–2 |  | 1.01 (1.01 to 1.02) | 1.50 (1.23 to 1.84) |
| Lowest glucose level, mmol/l – RCS (5.7, 7.5, 10.4) | 41,505 |  |  |
| Spline lowest glucose–1 |  | 1.31 (1.24 to 1.38) | 1.01 (0.89 to 1.14) |
| Spline lowest glucose–2 |  | 0.92 (0.87 to 0.96) | 1.11 (0.98 to 1.26) |
| Highest glucose level, mmol/l – RCS (6.3, 8.8, 13.2) | 37,125 |  |  |
| Spline highest glucose–1 |  | 1.62 (1.56 to 1.68) | 2.00 (1.70 to 2.37) |
| Spline highest glucose–2 |  | 0.66 (0.63 to 0.69) | 0.51 (0.43 to 0.61) |
| Lowest haemoglobin, g/dl – RCS (8.2, 11.3, 14.0) | 43,343 |  |  |
| Spline haemoglobin–1 |  | 0.84 (0.82 to 0.86) | 0.75 (0.69 to 0.81) |
| Spline haemoglobin–2 |  | 1.16 (1.12 to 1.20) | 1.34 (1.22 to 1.48) |

*Abbreviations:* ICU= Intensive Care Unit; RCS=Restricted Cubic Spline; SAP=Systolic Arterial Pressure

## Figure S7: Adjusted Odds Ratio (aOR) and 95% confidence intervals for continuous variables from the regression model analysing WLST decision.


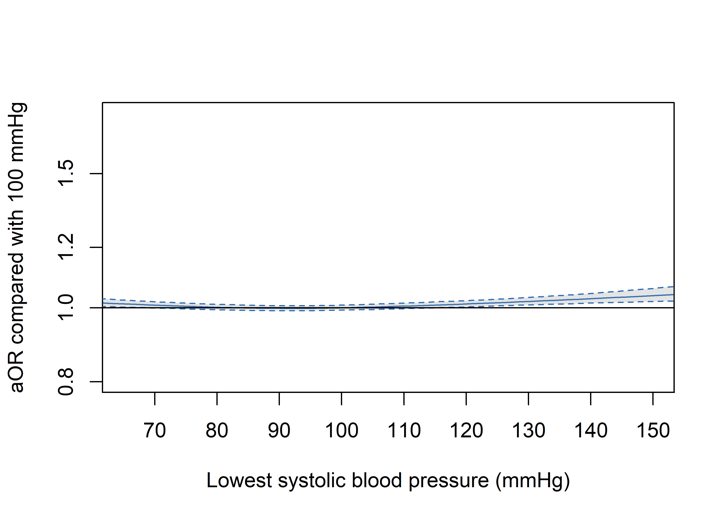

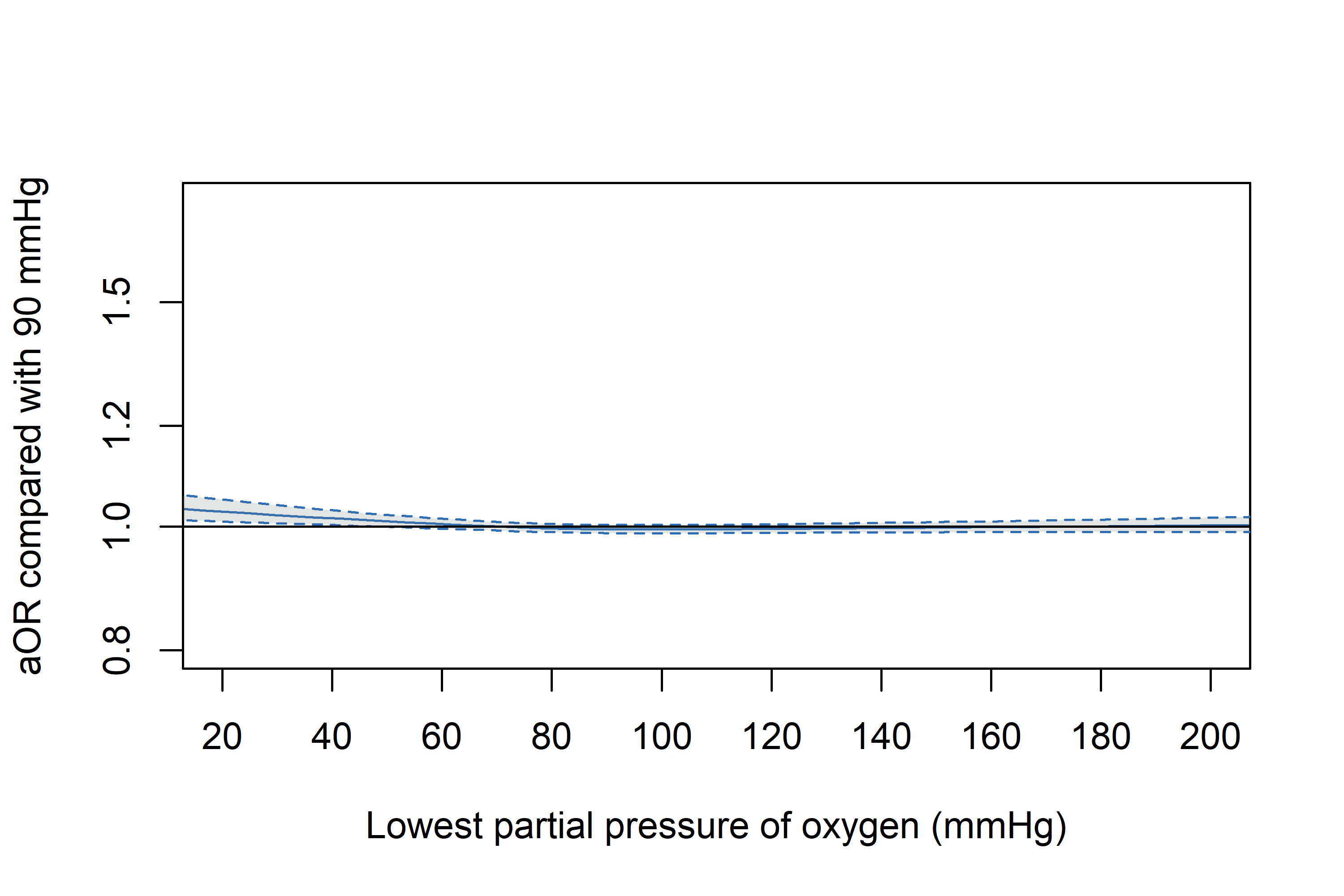

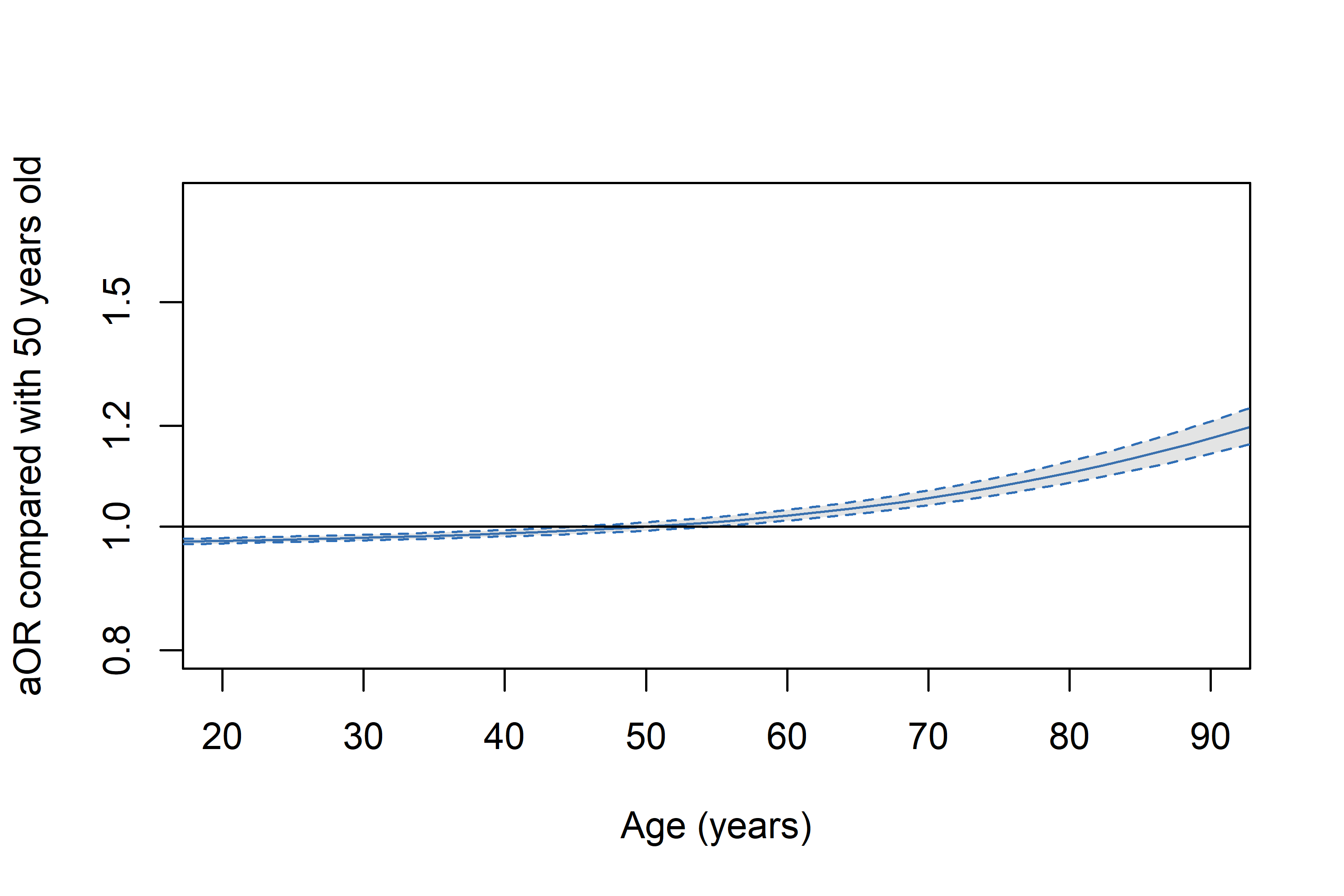
Panel A: Age Panel B: Lowest SAP Panel C: Lowest PaO_2_


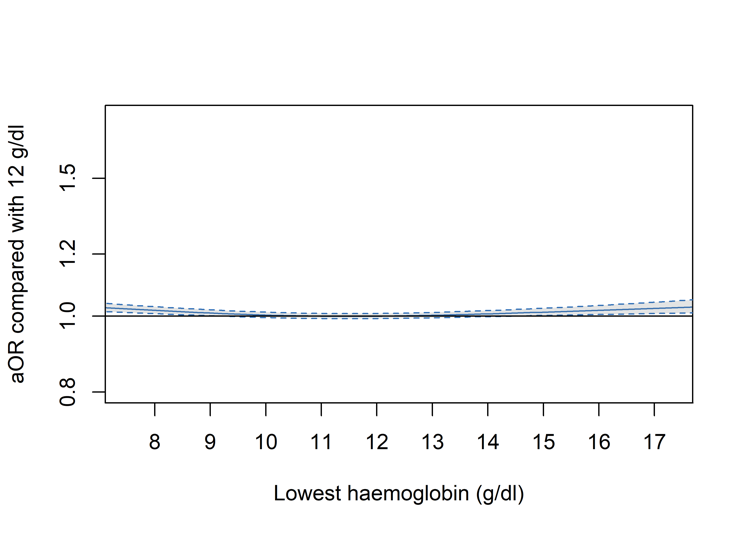

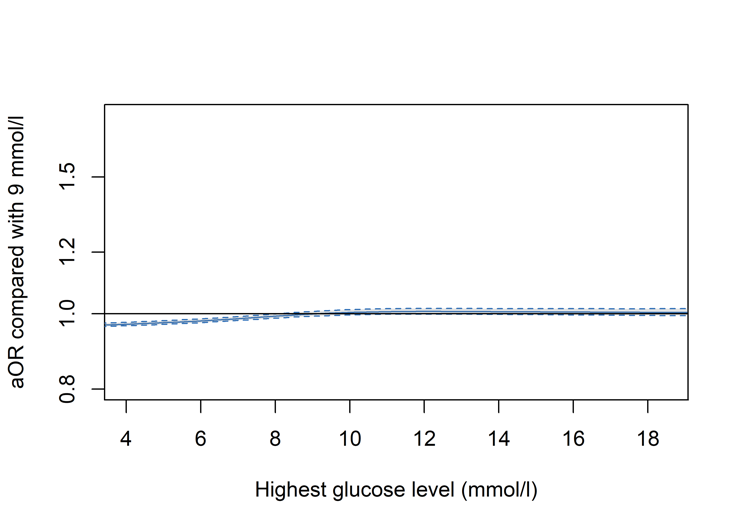

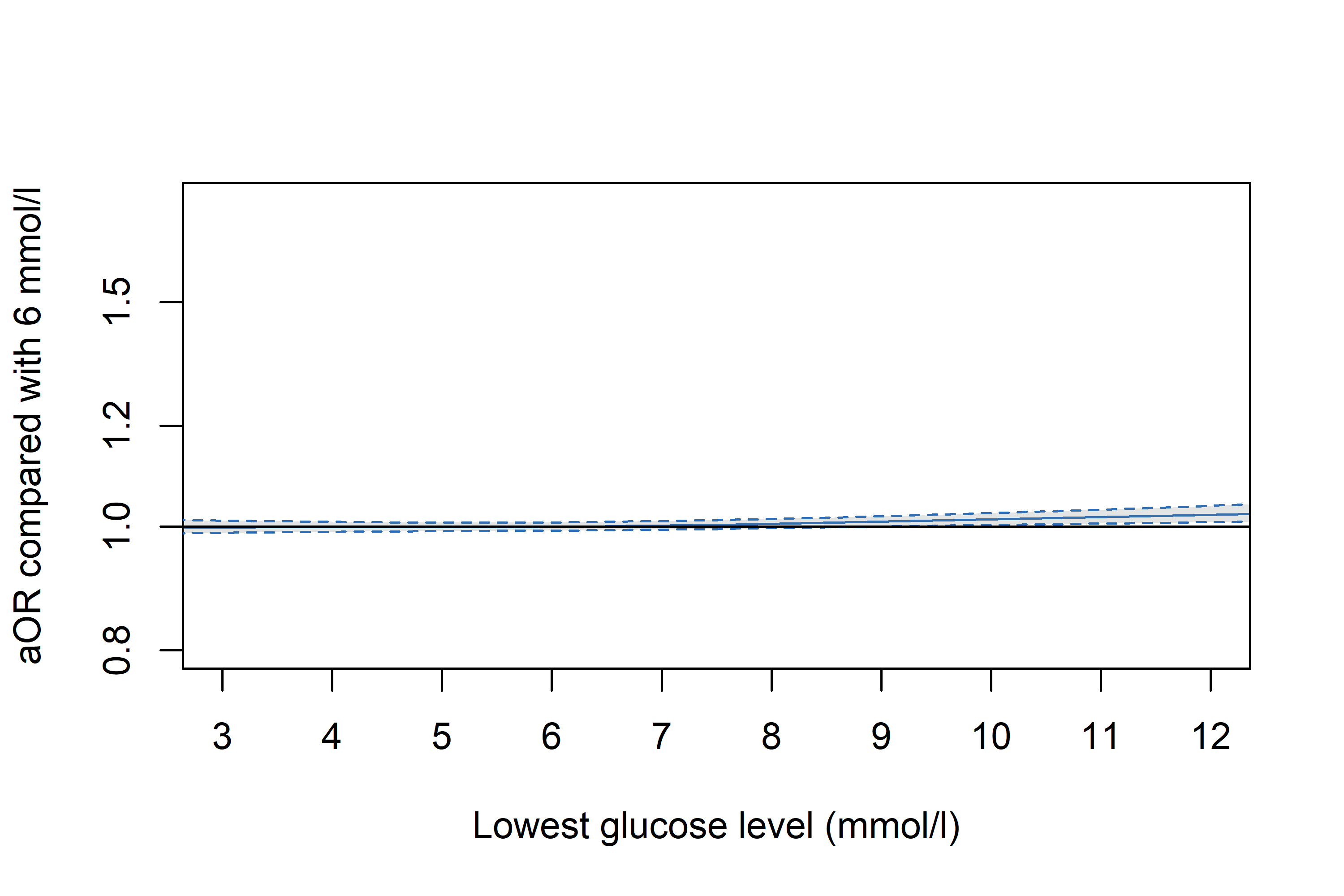
Panel D: Lowest glucose level Panel E: Highest glucose level Panel F: Lowest haemoglobin

*Footnote:* Adjusted ORs are represented on logarithmic scale.

## Figure S8: Final model performances for WLST decision


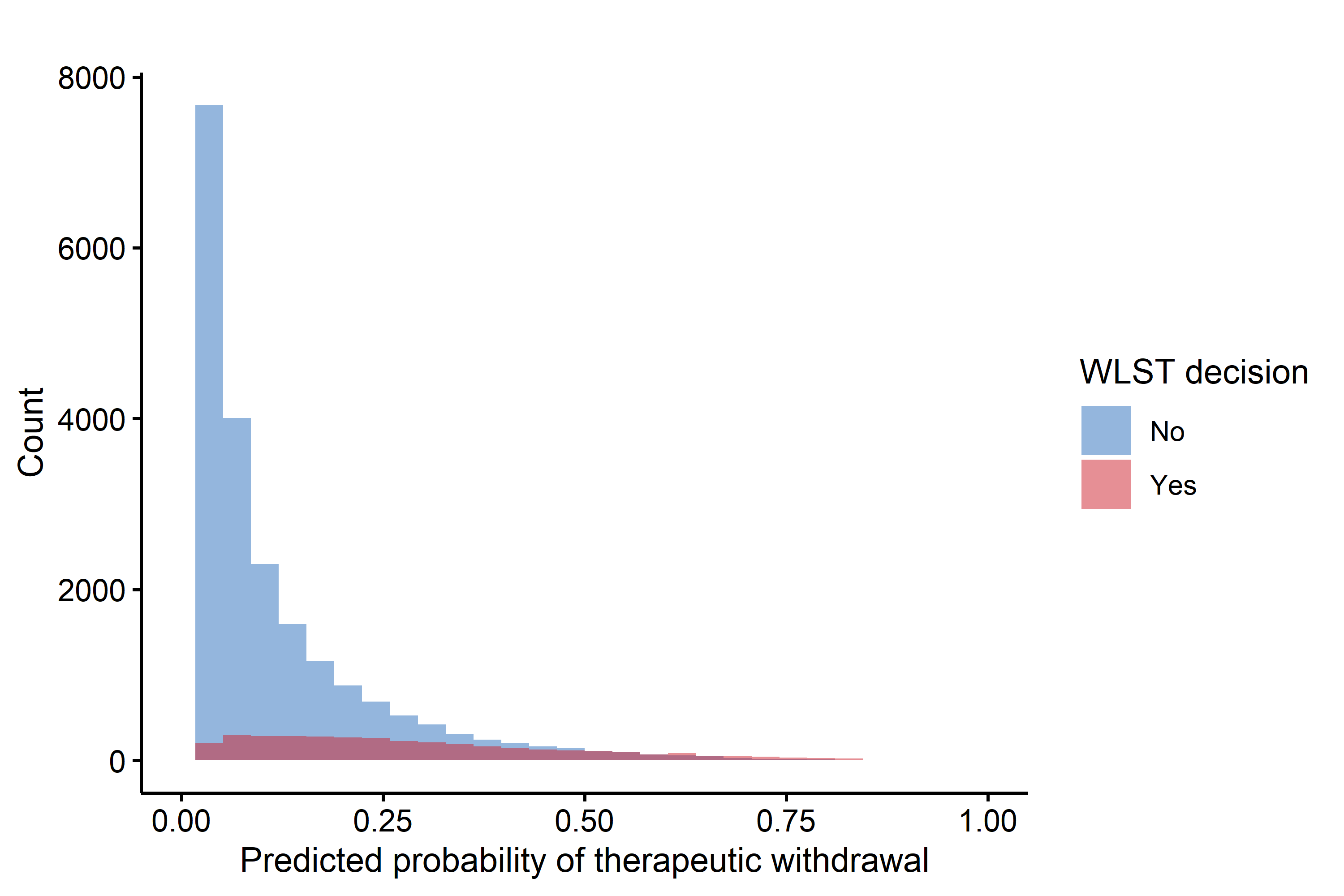
Panel A: Number of patients by WLST decision and predicted probability of WLST decision


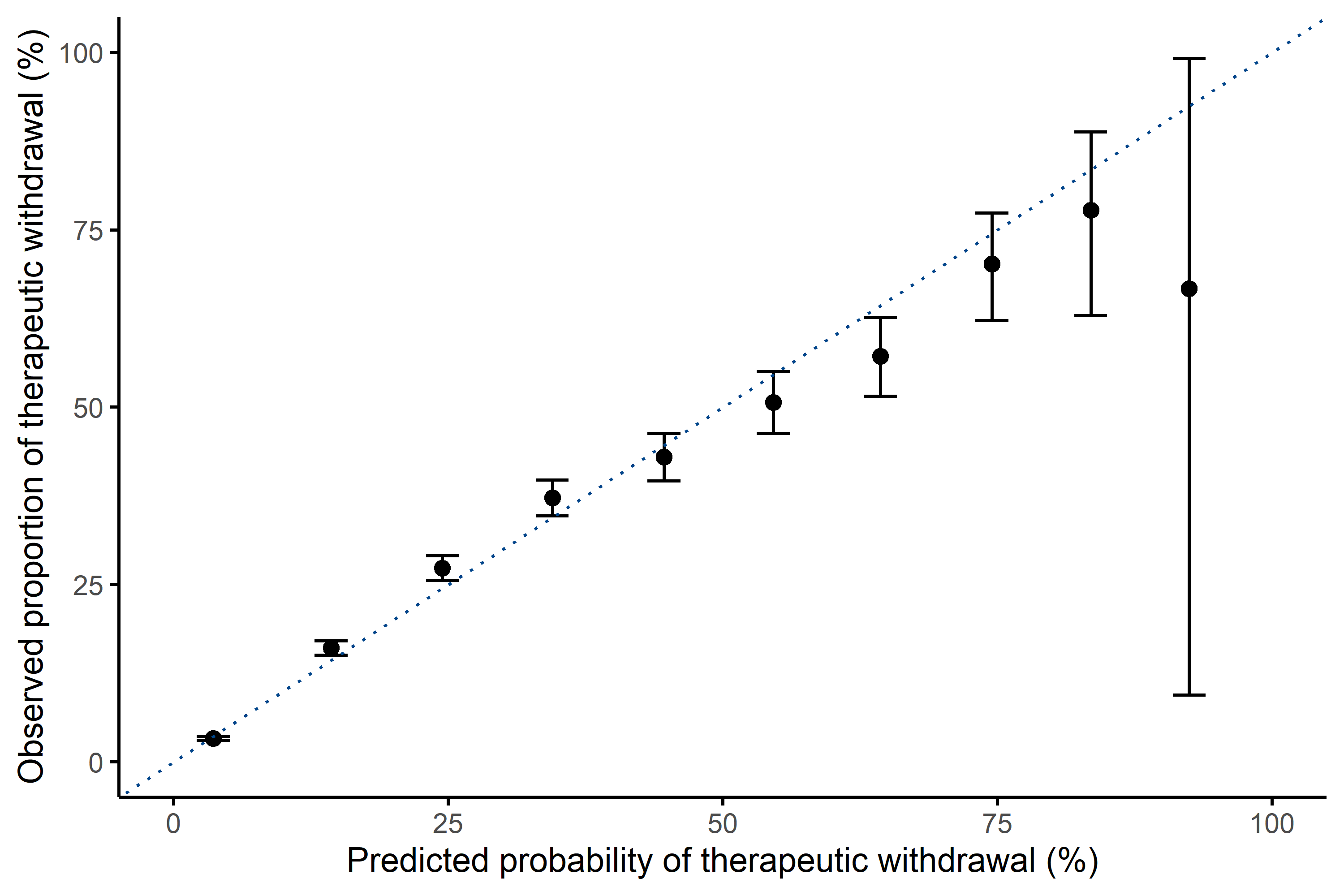
Panel B: Calibration plot

## Table S11: Sensitivity analysis for WLST decision

| **Covariates** | **Adjusted OR** |
| --- | --- |
| **Year of admission** (main independent covariate) |  |
| 2009–2010 | Reference |
| 2011–2012 | 1.16 (0.98 to 1.37) |
| 2013–2014 | 1.07 (0.91 to 1.27) |
| 2015–2016 | 1.45 (1.24 to 1.70) |
| 2017–2018 | 1.56 (1.33 to 1.81) |
| 2019–2020 | 1.81 (1.55 to 2.11) |
| 2021–2022 | 2.00 (1.72 to 2.34) |
| 2023–2024 | 2.63 (2.24 to 3.09) |
| Age, years – RCS (22, 51, 77) |  |
| Spline age–1 | 1.40 (1.28 to 1.55) |
| Spline age–2 | 1.52 (1.37 to 1.68) |
| Dependence at home |  |
| Living without assistance | Reference |
| Some assistance | 1.15 (1.04 to 1.27) |
| Total assistance | 1.02 (0.75 to 1.39) |
| Deprivation index |  |
| 1 (least deprived) | Reference |
| 2 | 1.06 (0.96 to 1.18) |
| 3 | 0.99 (0.89 to 1.10) |
| 4 | 0.96 (0.87 to 1.07) |
| 5 (most deprived) | 0.96 (0.86 to 1.06) |
| Type of ICU |  |
| ICU without neurosurgery | Reference |
| ICU with neurosurgery | 1.17 (0.98 to 1.40) |
| Neuroscience centre | 0.93 (0.79 to 1.10) |
| Surgery |  |
| No | Reference |
| Yes | 0.95 (0.88 to 1.03) |
| Pupils reactivity |  |
| Both reactive | Reference |
| One reactive | 2.44 (2.13 to 2.80) |
| Both unreactive | 3.67 (3.41 to 3.95) |
| GCS – motor component |  |
| Localise or obeys | Reference |
| Normal flexion | 2.55 (2.08 to 3.12) |
| Abnormal flexion | 5.16 (4.04 to 6.60) |
| Extension | 8.44 (6.84 to 10.40) |
| None | 7.82 (6.93 to 8.84) |
| Untestable | 5.75 (5.17 to 6.39) |
| Lowest SAP, mmHg – RCS (70, 94, 105, 130) |  |
| Spline SAP–1 | 0.80 (0.75 to 0.85) |
| Spline SAP–2 | 1.36 (1.07 to 1.73) |
| Spline SAP–3 | 0.54 (0.16 to 1.79) |
| Lowest PaO_2_, mmHg – RCS (62, 80, 116) |  |
| Spline PaO_2_–1 | 0.74 (0.63 to 0.87) |
| Spline PaO_2_–2 | 1.33 (1.13 to 1.56) |
| Lowest glucose level, mmol/l – RCS (5.7, 7.5, 10.4) |  |
| Spline lowest glucose–1 | 2.54 (1.95 to 3.32) |
| Spline lowest glucose–2 | 0.48 (0.38 to 0.60) |
| Highest glucose level, mmol/l – RCS (6.3, 8.8, 13.2) |  |
| Spline highest glucose–1 | 1.21 (1.10 to 1.32) |
| Spline highest glucose–2 | 0.93 (0.86 to 1.02) |
| Lowest haemoglobin, g/dl – RCS (8.2, 11.3, 14.0) |  |
| Spline haemoglobin–1 | 0.74 (0.70 to 0.79) |
| Spline haemoglobin–2 | 1.34 (1.24 to 1.44) |

*Abbreviations:* ICU= Intensive Care Unit; RCS=Restricted Cubic Spline; SAP=Systolic Arterial Pressure

## Table S12: Main physiological parameters recorded during the first 24 hours following ICU admission.

| **Variables** | **Overall** | **April 2009**–**2010** | **2011**–**2012** | **2013**–**2014** | **2015**–**2016** | **2017**–**2018** | **2019**–**2020** | **2021**–**2022** | **2023**–  **March 2024** |
| --- | --- | --- | --- | --- | --- | --- | --- | --- | --- |
|  | N = 45,684 | N = 4,072 | N = 5,011 | N = 5,386 | N = 6,919 | N = 7,390 | N = 6,730 | N = 6,318 | N = 3,858 |
| Lowest PaO_2_, mmHg – mean (SD) | 87 (42) | 106 (85) | 99 (51) | 91 (43) | 87 (35) | 84 (29) | 82 (27) | 80 (22) | 79 (24) |
| Missing – no. (%) | 4,419 (9.7) | 388 (9.5) | 583 (11.6) | 522 (9.7) | 803 (11.6) | 788 (10.7) | 620 (9.2) | 429 (6.8) | 286 (7.4) |
| PaO_2_ ranges – no. (%) |  |  |  |  |  |  |  |  |  |
| 80–100 | 12,852 (31.1) | 1,084 (29.4) | 1,282 (29.0) | 1,509 (31.0) | 1,972 (32.2) | 2,221 (33.6) | 1,957 (32.0) | 1,776 (30.2) | 1,051 (29.4) |
| <80 | 20,747 (50.3) | 1,359 (36.9) | 1,725 (39.0) | 2,197 (45.2) | 2,971 (48.6) | 3,401 (51.5) | 3,380 (55.3) | 3,528 (59.9) | 2,186 (61.2) |
| >100 | 7,666 (18.6) | 1,241 (33.7) | 1,421 (32.1) | 1,158 (23.8) | 1,173 (19.2) | 980 (14.8) | 773 (12.7) | 585 (9.9) | 335 (9.4) |
| PaCO_2_, mmHg |  |  |  |  |  |  |  |  |  |
| Associated with lowest pH – mean (SD) | 43 (15) | 46 (35) | 43 (16) | 43 (12) | 43 (10) | 43 (10) | 43 (9) | 42 (9) | 42 (9) |
| Associated with lowest PaO_2_ – mean (SD) | 39 (13) | 41 (31) | 39 (14) | 38 (10) | 39 (8) | 39 (8) | 38 (8) | 38 (7) | 38 (7) |
| Missing – no. (%) | 4,423 (9.7) | 389 (9.6) | 583 (11.6) | 522 (9.7) | 803 (11.6) | 790 (10.7) | 621 (9.2) | 428 (6.8) | 286 (7.4) |
| PaCO_2_ ranges – no. (%) |  |  |  |  |  |  |  |  |  |
| 35–45 | 15,037 (36.4) | 1,287 (34.9) | 1,512 (34.1) | 1,672 (34.4) | 2,218 (36.3) | 2,415 (36.6) | 2,229 (36.5) | 2,280 (38.7) | 1,424 (39.9) |
| <35 | 12,356 (29.9) | 1,158 (31.4) | 1,369 (30.9) | 1,494 (30.7) | 1,741 (28.5) | 1,859 (28.2) | 1,832 (30.0) | 1,808 (30.7) | 1,095 (30.7) |
| >45 | 13,869 (33.6) | 1,238 (33.6) | 1,547 (34.9) | 1,698 (34.9) | 2,157 (35.3) | 2,326 (35.2) | 2,048 (33.5) | 1,802 (30.6) | 1,053 (29.5) |
| pH level |  |  |  |  |  |  |  |  |  |
| Mean highest (SD) | 7.39 (0.08) | 7.39 (0.08) | 7.39 (0.08) | 7.39 (0.08) | 7.39 (0.08) | 7.39 (0.08) | 7.39 (0.08) | 7.40 (0.08) | 7.40 (0.08) |
| Mean lowest (SD) | 7.33 (0.09) | 7.33 (0.09) | 7.33 (0.09) | 7.33 (0.09) | 7.33 (0.09) | 7.33 (0.09) | 7.33 (0.09) | 7.34 (0.09) | 7.34 (0.09) |
| Missing – no. (%) | 6,660 (14.6) | 648 (15.9) | 856 (17.1) | 561 (10.4) | 1,224 (17.7) | 1,245 (16.9) | 997 (14.8) | 781 (12.4) | 288 (7.5) |
| pH ranges – no. (%) |  |  |  |  |  |  |  |  |  |
| 7.35–7.45 | 13,849 (35.4) | 1,147 (33.5) | 1,443 (34.7) | 1,651 (34.2) | 1,980 (34.8) | 2,214 (36.0) | 2,068 (36.1) | 2,058 (37.2) | 1,288 (36.1) |
| <7.35 | 17,946 (45.9) | 1,619 (47.3) | 1,964 (47.3) | 2,357 (48.8) | 2,682 (47.1) | 2,782 (45.3) | 2,641 (46.1) | 2,311 (41.7) | 1,590 (44.5) |
| >7.45 | 7,289 (18.6) | 658 (19.2) | 748 (18.0) | 817 (16.9) | 1,033 (18.1) | 1,149 (18.7) | 1,024 (17.9) | 1,168 (21.1) | 692 (19.4) |
| SAP, mmHg |  |  |  |  |  |  |  |  |  |
| Mean highest (SD) | 158 (25) | 159 (25) | 157 (25) | 158 (26) | 157 (25) | 159 (26) | 158 (25) | 157 (25) | 158 (25) |
| Mean lowest (SD) | 100 (19) | 102 (19) | 100 (20) | 100 (19) | 99 (19) | 99 (20) | 99 (19) | 100 (18) | 99 (19) |
| Missing – no. (%) | 386 (0.8) | 72 (1.8) | 67 (1.3) | 42 (0.8) | 46 (0.7) | 51 (0.7) | 48 (0.7) | 29 (0.5) | 31 (0.8) |
| SAP ranges – no. (%) |  |  |  |  |  |  |  |  |  |
| ≥100 | 22,739 (50.2) | 2,257 (56.4) | 2,637 (53.3) | 2,712 (50.7) | 3,317 (48.3) | 3,501 (47.7) | 3,279 (49.1) | 3,126 (49.7) | 1,910 (49.9) |
| <100 | 22,559 (49.8) | 1,743 (43.3) | 2,307 (46.5) | 2,632 (49.1) | 3,556 (51.6) | 3,838 (52.2) | 3,403 (50.8) | 3,163 (50.2) | 1,917 (50.0) |
| Temperature, °C – mean (SD) |  |  |  |  |  |  |  |  |  |
| Mean highest (SD) | 37.6 (0.9) | 37.5 (0.9) | 37.4 (0.9) | 37.5 (0.9) | 37.6 (0.9) | 37.6 (0.9) | 37.6 (0.9) | 37.6 (0.8) | 37.6 (0.8) |
| Mean lowest (SD) | 35.7 (1.2) | 35.7 (1.2) | 35.6 (1.3) | 35.7 (1.2) | 35.7 (1.1) | 35.8 (1.2) | 35.7 (1.2) | 35.7 (1.1) | 35.7 (1.2) |
| Missing – no. (%) | 1,334 (2.9) | 243 (6.0) | 242 (4.8) | 151 (2.8) | 171 (2.5) | 165 (2.2) | 145 (2.2) | 125 (2.0) | 92 (2.4) |
| Temperature ranges – no. (%) |  |  |  |  |  |  |  |  |  |
| 36–38 | 15,613 (35.2) | 1,232 (32.2) | 1,600 (33.6) | 1,775 (33.9) | 2,366 (35.1) | 2,577 (35.7) | 2,334 (35.4) | 2,329 (37.6) | 1,400 (37.2) |
| <36 | 17,532 (39.5) | 1,691 (44.2) | 2,087 (43.8) | 2,168 (41.4) | 2,606 (38.6) | 2,625 (36.3) | 2,538 (38.5) | 2,397 (38.7) | 1,420 (37.7) |
| >38 | 11,205 (25.3) | 906 (23.7) | 1,082 (22.7) | 1,292 (24.7) | 1,776 (26.3) | 2,023 (28.0) | 1,713 (26.0) | 1,467 (23.7) | 946 (25.1) |
| Glucose level, mmol/l |  |  |  |  |  |  |  |  |  |
| Mean highest (SD) | 6.4 (2.0) | 6.2 (2.7) | 6.2 (1.9) | 6.3 (1.9) | 6.4 (1.8) | 6.5 (2.0) | 6.5 (1.9) | 6.3 (1.9) | 6.5 (2.1) |
| Mean lowest (SD) | 9.4 (3.3) | 9 (3.7) | 9.1 (3.3) | 9.5 (3.3) | 9.4 (3.3) | 9.6 (3.3) | 9.6 (3.3) | 9.46 (3.3) | 9.7 (3.5) |
| Missing – no. (%) | 4,179 (9.1) | 1,575 (38.7) | 549 (11.0) | 372 (6.9) | 492 (7.1) | 466 (6.3) | 324 (4.8) | 232 (3.7) | 169 (4.4) |
| Glucose ranges – no. (%) |  |  |  |  |  |  |  |  |  |
| 4.5–10 | 26,463 (63.8) | 1,685 (67.5) | 2,976 (66.7) | 3,173 (63.3) | 4,063 (63.2) | 4,358 (62.9) | 4,012 (62.6) | 3,870 (63.6) | 2,326 (63.1) |
| <4.5 | 2,915 (7.0) | 293 (11.7) | 485 (10.9) | 468 (9.3) | 567 (8.8) | 539 (7.8) | 515 (8.0) | 559 (9.2) | 296 (8.0) |
| >10 | 12,127 (29.2) | 519 (20.8) | 1,001 (22.4) | 1,373 (27.4) | 1,797 (28.0) | 2,027 (29.3) | 1,879 (29.3) | 1,657 (27.2) | 1,067 (28.9) |
| Sodium level, mmol/l |  |  |  |  |  |  |  |  |  |
| Mean highest (SD) | 143 (6) | 143 (6) | 143 (6) | 142 (6) | 142 (6) | 142 (6) | 142 (6) | 143 (7) | 143 (7) |
| Mean lowest (SD) | 138 (5.5) | 138 (5) | 138 (5) | 138 (5) | 138 (5) | 138 (5) | 138 (6) | 138 (6) | 138 (6) |
| Missing – no. (%) | 11,836 (25.9) | 1,033 (25.4) | 1,178 (23.5) | 1,054 (19.6) | 1,621 (23.4) | 1,872 (25.3) | 1,914 (28.4) | 1,717 (27.2) | 1,083 (28.1) |
| Sodium ranges – no. (%) |  |  |  |  |  |  |  |  |  |
| 135–145 | 19,206 (56.1) | 1,753 (57.7) | 2,207 (57.6) | 2,405 (55.5) | 3,028 (57.2) | 3,137 (56.9) | 2,763 (57.4) | 2,500 (54.3) | 1,413 (50.9) |
| <135 | 6,736 (19.7) | 524 (17.2) | 720 (18.8) | 944 (21.8) | 1,041 (19.6) | 1,093 (19.8) | 909 (18.9) | 937 (20.4) | 568 (20.5) |
| >145 | 7,906 (23.1) | 728 (24) | 862 (22.5) | 924 (21.3) | 1,171 (22.1) | 1,232 (22.3) | 1,095 (22.7) | 1,131 (24.6) | 763 (27.5) |
| Lowest haemoglobin, g/dl – mean (SD) | 11.2 (2.2) | 10.7 (2.4) | 10.8 (2.3) | 11.1 (2.3) | 11.3 (2.2) | 11.3 (2.2) | 11.4 (2.1) | 11.4 (2.2) | 11.4 (2.1) |
| Missing – no. (%) | 2,341 (5.1) | 281 (6.9) | 315 (6.3) | 268 (5.0) | 316 (4.6) | 367 (5) | 320 (4.8) | 285 (4.5) | 189 (4.9) |
| Haemoglobin ranges – no. (%) |  |  |  |  |  |  |  |  |  |
| ≥12 | 16,869 (38.9) | 1,168 (30.8) | 1,554 (33.1) | 1,869 (36.5) | 2,647 (40.1) | 2,838 (40.4) | 2,705 (42.2) | 2,548 (42.2) | 1,540 (42.0) |
| 9–11.9 | 19,268 (44.5) | 1,715 (45.2) | 2,063 (43.9) | 2,249 (43.9) | 2,892 (43.8) | 3,127 (44.5) | 2,851 (44.5) | 2,694 (44.7) | 1,677 (45.7) |
| 7–8.9 | 6,053 (14.0) | 684 (18.0) | 863 (18.4) | 859 (16.8) | 936 (14.2) | 922 (13.1) | 726 (11.3) | 685 (11.4) | 378 (10.3) |
| <7 | 1,153 (2.7) | 224 (5.9) | 216 (4.6) | 141 (2.8) | 128 (1.9) | 136 (1.9) | 128 (2.0) | 106 (1.8) | 74 (2.0) |
| Lowest platelet count, G/l – mean (SD) | 195 (89) | 192 (102) | 191 (97) | 191 (94) | 193 (83) | 194 (83) | 198 (84) | 200 (87) | 199 (86) |
| Missing – no. (%) | 2,756 (6.0) | 329 (8.1) | 366 (7.3) | 295 (5.5) | 378 (5.5) | 432 (5.9) | 360 (5.4) | 364 (5.8) | 232 (6.0) |
| Platelet ranges – no. (%) |  |  |  |  |  |  |  |  |  |
| ≥75 | 40,636 (94.7) | 3,493 (93.3) | 4,354 (93.7) | 4,785 (94.0) | 6,237 (95.4) | 6,611 (95.0) | 6,049 (95.0) | 5,656 (95.0) | 3,451 (95.2) |
| <75 | 2,292 (5.3) | 250 (6.7) | 291 (6.3) | 306 (6.0) | 304 (4.6) | 347 (5.0) | 321 (5.0) | 298 (5.0) | 175 (4.8) |

*Abbreviations:* SAP=Systolic Arterial Pressure; SD=Standard Deviation

# References

1. Harrison DA, Brady AR, Rowan K, et al. Case mix, outcome and length of stay for admissions to adult, general critical care units in England, Wales and Northern Ireland: the Intensive Care National Audit & Research Centre Case Mix Programme Database. *Critical Care 2004 8:2* 2004-02-26;8(2) doi: 10.1186/cc2834

2. Young JD, Goldfrad C, Rowan K. Development and testing of a hierarchical method to code the reason for admission to intensive care units: the ICNARC Coding Method. *British Journal of Anaesthesia* 2001/10/01;87(4) doi: 10.1093/bja/87.4.543

3. Shankar-Hari M, Phillips GS, Levy ML, et al. Developing a New Definition and Assessing New Clinical Criteria for Septic Shock: For the Third International Consensus Definitions for Sepsis and Septic Shock (Sepsis-3). *JAMA* 2016;315(8):775-87. doi: 10.1001/jama.2016.0289

4. Singer M, Deutschman CS, Seymour CW, et al. Consensus Definitions for Sepsis and Septic Shock. *JAMA* 2016/02/23;315(8) doi: 10.1001/jama.2016.0287

5. Wagner DP, Knaus WA, Draper EA. Statistical validation of a severity of illness measure. *Am J Public Health* 2011-10-07;73(8) doi: 10.2105/AJPH.73.8.878

6. Steyerberg EW, Mushkudiani N, Perel P, et al. Predicting Outcome after Traumatic Brain Injury: Development and International Validation of Prognostic Scores Based on Admission Characteristics. *PLoS Med* 5 Aug 2008;5(8) doi: 10.1371/journal.pmed.0050165

7. Nathens AB, Cryer HG, Fildes J. The American College of Surgeons Trauma Quality Improvement Program. *Surgical Clinics of North America* 2012/04/01;92(2) doi: 10.1016/j.suc.2012.01.003

8. Perel P, Arango M, Clayton T, et al. Predicting outcome after traumatic brain injury: practical prognostic models based on large cohort of international patients. *BMJ* 2008-02-21;336(7641) doi: 10.1136/bmj.39461.643438.25

9. Dijkland SA, Foks KA, Polinder S, et al. Prognosis in Moderate and Severe Traumatic Brain Injury: A Systematic Review of Contemporary Models and Validation Studies. *Journal of Neurotrauma* 2020;37(1):1-13. doi: 10.1089/neu.2019.6401

10. Eagle SR, Nwachuku E, Elmer J, et al. Performance of CRASH and IMPACT Prognostic Models for Traumatic Brain Injury at 12 and 24 Months Post-Injury. *Neurotrauma Reports* 2023;4(1):118-23. doi: 10.1089/neur.2022.0082

11. Yue JK, Lee YM, Sun X, et al. Performance of the IMPACT and CRASH prognostic models for traumatic brain injury in a contemporary multicenter cohort: a TRACK-TBI study. *J Neurosurg* 2024-03-15;141(2) doi: 10.3171/2023.11.JNS231425

12. Maas AIR, Menon DK, Manley GT, et al. Traumatic brain injury: progress and challenges in prevention, clinical care, and research. *The Lancet Neurology* 2022;21(11):1004-60. doi: 10.1016/S1474-4422(22)00309-X

13. Harrison DA, Griggs KA, Prabhu G, et al. External Validation and Recalibration of Risk Prediction Models for Acute Traumatic Brain Injury among Critically Ill Adult Patients in the United Kingdom. *Journal of Neurotrauma* 2015 Oct 1;32(19) doi: 10.1089/neu.2014.3628

14. Hoiland RL, Fisher JA, Ainslie PN. Regulation of the Cerebral Circulation by Arterial Carbon Dioxide. *Comprehensive Physiology* 2019;9 doi: 10.1002/cphy.c180021

15. Hawryluk GWJ, Aguilera S, Buki A, et al. A management algorithm for patients with intracranial pressure monitoring: the Seattle International Severe Traumatic Brain Injury Consensus Conference (SIBICC). *Intensive Care Med* 2019;45(12):1783-94. doi: 10.1007/s00134-019-05805-9

16. ACS TQP Best Practices Guidelines. *ACS*

17. Gupte R, Brooks W, Vukas R, et al. Sex Differences in Traumatic Brain Injury: What We Know and What We Should Know. *Journal of Neurotrauma* 2019 Oct 23;36(22) doi: 10.1089/neu.2018.6171

18. Ma C, Wu X, Shen X, et al. Sex differences in traumatic brain injury: a multi-dimensional exploration in genes, hormones, cells, individuals, and society. *Chinese Neurosurgical Journal 2019 5:1* 2019-10-04;5(1) doi: 10.1186/s41016-019-0173-8

19. Starkey NJ, Duffy B, Jones K, et al. Sex differences in outcomes from mild traumatic brain injury eight years post-injury. *PLoS One* 2022 May 27;17(5) doi: 10.1371/journal.pone.0269101

20. Haines KL, Nguyen BP, Vatsaas C, et al. Socioeconomic Status Affects Outcomes After Severity-Stratified Traumatic Brain Injury. *J Surg Res* 2019/03/01;235 doi: 10.1016/j.jss.2018.09.072

21. Humphries TJ, Ingram S, Sinha S, et al. The effect of socioeconomic deprivation on 12 month Traumatic Brain Injury (TBI) outcome. *Brain Inj* 2020-2-23;34(3) doi: 10.1080/02699052.2020.1715481

22. Williamson T, Ryser MD, Ubel PA, et al. Withdrawal of Life-supporting Treatment in Severe Traumatic Brain Injury. *JAMA Surg* 2020/08/01;155(8) doi: 10.1001/jamasurg.2020.1790

23. Roberts CJ, Barber J, Temkin NR, et al. Clinical Outcomes After Traumatic Brain Injury and Exposure to Extracranial Surgery. *JAMA Surg* 2024/03/01;159(3) doi: 10.1001/jamasurg.2023.6374

24. Cooper Z, Rivara FP, Wang J, et al. Withdrawal of Life-Sustaining Therapy in Injured Patients: Variations Between Trauma Centers and Nontrauma Centers. *Journal of Trauma and Acute Care Surgery* May 2009;66(5) doi: 10.1097/TA.0b013e31819ea047

25. Huijben JA, Dixit A, Stocchetti N, et al. Use and impact of high intensity treatments in patients with traumatic brain injury across Europe: a CENTER-TBI analysis. *Critical Care 2021 25:1* 2021-02-23;25(1) doi: 10.1186/s13054-020-03370-y

26. Singer M, Deutschman CS, Seymour CW, et al. The Third International Consensus Definitions for Sepsis and Septic Shock (Sepsis-3). *JAMA* 2016;315(8):801-10. doi: 10.1001/jama.2016.0287

# Original Statistical Analysis Plan (SAP)

**Approval**

Name: Xavier Chapalain Role: Chief Investigator

Date: 06-03-2025

Name: David A Harrison Role: Lead Statistician

Date: 06-03-2025

**Review**

Name: David K Menon Role: Scientific Director

Name: Olivier Huet Role: Scientific Director

Name: Kathy M Rowan Role: Scientific Director

Name: Paul R Mouncey Role: Scientific Director

**Objectives**

Primary objective

The primary aim of this study (Research Objective 1: RO1) will be to examine trends in hospital mortality for TBI patients from April 2009 to March 2024.

Secondary objectives

The main secondary objective (RO2) will be to evaluate the proportion of withdrawal of life-sustaining therapy decisions over time and its determinants.

Other objective

Another objective (RO3) will be to describe TBI patients’ exposure to the following brain insults during the first 24 hours, over time: hypotension, hypoxemia, hyperoxia, hyponatremia, hypernatremia, hypothermia, hyperthermia, hypoglycaemia, hyperglycaemia, hypocapnia, hypercapnia, alkalosis, acidosis, anaemia and thrombopenia.

**Methods**

Study design

This is an observational cohort study from the ICNARC (Intensive Care National Audit and Research Centre) Case Mix Program (CMP).

Population

a/TBI cohort:

All TBI patients included in the CMP from the 1^st^ of April 2009 to the 31^st^ of March 2024 will be eligible to participate to the TBI cohort. The period of time was restricted to the patients admitted after March 2009, because a significant proportion of neurocritical care units participated to the CMP since this date. TBI patients were identified on the CMP according the ICNARC coding method used to describe primary and secondary reason for ICU admission. The following code will be retained to identify eligible patients: ‘primary brain injury’, ‘traumatic subdural haemorrhage’, ‘traumatic subarachnoid haemorrhage’, ‘extradural haemorrhage’, ‘focal brain injury’ and ‘non accidental injury to brain’. For all patients, only the first ICU admission will be extracted for data analysis. Subsequent ICU admissions won’t not analysed.

b/Other ICU patients:

All other patients included in the CMP at the same period of time (April 2009 to March 2024) will also be included in the study, and consisted to the comparative cohort. The comparative cohort will be separated in three groups: patients with sepsis (without TBI or trauma), patients with trauma (without sepsis or TBI) and the other ICU patients (without TBI, trauma or sepsis). Sepsis will be defined using a previously derived approximation to the definition of sepsis that can be applied consistently over the full time period ^3 26^. Trauma patients will be identified on the CMP according the ICNARC coding method used to describe primary and secondary reason for ICU admission. For all patients, only the first ICU admission will be extracted for data analysis. Subsequent ICU admissions won’t be analysed. This comparative cohort will only be used to illustrate the trend of in-hospital mortality in all patients admitted concomitantly to the TBI patients in the ICU. Except a descriptive analysis, no other statistical analysis will be performed on the comparative cohort. Three descriptive analysis of mortality rate will be performed:

One describing hospital mortality for TBI patients, trauma patients (without TBI or sepsis), sepsis patients (without TBI or trauma) and all other ICU patients

One describing hospital mortality for TBI patients, trauma patients (without TBI or sepsis), sepsis patients (without TBI or trauma) and all other ICU patients, excluding all COVID 19 patients

One describing hospital mortality for TBI patients and matching ICU patients (Sepsis, trauma and other patients). Matching will be performed according to four characteristics: age, type of hospital, year of ICU admission and baseline APACHE 2 score. Matching will be performed using MatchIt package (R statistical software), without replacement, with the ‘exact’ method for categorical variables (type of hospital and year of ICU admission) and the ‘nearest’ method for continuous variables (age and APACHE 2 score) with a caliper of 0.1. Ratios between TBI patients and each comparative population will be 1:2 for matching with trauma patients, 1:3 for matching with sepsis patients and 1:5 for all other ICU patients.

Outcome definitions

The primary outcome (RO1) will be in-hospital mortality. This outcome was prospectively recorded on the CMP, and corresponded to the ultimate status (died or alive) at hospital discharge.

The main secondary outcome (RO2) will be the proportion of therapeutic withdrawal. Therapeutic withdrawals were defined as where all clinically indicated treatments, other than comfort measures, were withdrawn on the grounds of lack of benefit to the patient.

Additional analysis (RO3): We will evaluate the proportion of patients exposed to each of the following brain insults during the first 24 hours in ICU:

- Hypotension defined by a Systolic Arterial blood Pressure (SAP) < 100 mmHg
- Hypoxemia defined by a PaO_2_ < 80 mmHg
- Hyperoxia defined by a PaO_2_ > 100 mmHg
- Hyponatremia defined by a sodium level < 135 mmol/l
- Hypernatremia defined by a sodium level > 145 mmol/l
- Hypothermia defined by temperature < 36°C
- Hyperthermia defined by temperature > 38°C
- Hypoglycaemia defined by a glucose level < 80 mg/dl
- Hyperglycaemia defined by a glucose level > 180 mg/dl
- Hypocapnia defined by a PaCO_2_ < 35 mmHg
- Hypercapnia defined by a PaCO_2_ > 45 mmHg
- Alkalosis defined by a pH level > 7.45
- Acidosis defined by a pH level < 7.35
- Anaemia defined by a haemoglobin level < 7 g/dl
- Thrombopenia defined by a platelet count < 75G/l

Each of the brain insults were defined regarding international guidelines from the American College of Surgeon from their Trauma Quality Improvement Program. These guidelines delineate early management principles for TBI patients and are broadly recognised as a gold standard. We will also explore their association with hospital mortality.

Covariate definitions

For the primary outcome analysis (RO1: in-hospital mortality), we will study the effect of the following covariates:

- Socio-demographic characteristics: age and sex.
- Type of ICU divided in: ‘general ICU without neurosurgery’, ‘general ICU with neurosurgery’, and ‘specialist neuroscience ICU’. For hospital with one ‘specialist neuroscience ICU’ and one ‘general ICU’, these general ICU will be considered as general ICU with neurosurgery.
- Any surgical procedure before ICU admission (yes vs. no).
- Two important predictors for mortality after TBI: pupil reactivity and motor component of the Glasgow Coma Scale (GCS), both recorded during the first 24 hours.
- Some physiological parameters associated with secondary brain insults recorded in the first 24 hours: SAP, PaO_2_, glucose level and/or an haemoglobin.

These main covariates were chosen regarding previous studies reported an association with mortality for TBI patients, and a plausible impact on mortality in the TBI cohort.

Some other covariates will eventually be included in the final model if there will be associated with hospital mortality in univariable analysis, increase regression model fitting and if there will not introduce collinearity. These candidate variables will be: Deprivation index (from 1 to 5), Size of ICU (defined by the number of beds: <10, 10-20 and >20) and PaCO_2_. Further details are provided in ‘Statistical analysis’ part.

For secondary outcome analysis (RO2: therapeutic withdrawal), we will describe the effect of the following covariate:

- Socio-demographic characteristics:
  - Age: calculated age at ICU admission
  - Dependence at home defined by the degree of autonomy at home: living without assistance, some assistance or total assistance
- Type of ICU divided in: ‘general ICU without neurosurgery’, ‘general ICU with neurosurgery’, and ‘specialist neuroscience ICU’. For hospital with one ‘specialist neuroscience ICU’ and one ‘general ICU’, these general ICU will be considered as general ICU with neurosurgery.
- Two important predictors for poor neurological prognosis after TBI: pupils reactivity and motor component of the GCS, both recorded during the first 24 hours.
- Any surgical procedure before ICU admission (yes vs. no)

**Statistical analysis**

Descriptive analysis

Continuous variables will be described as mean and standard deviation (SD) or median and interquartile range (IQR) according to the distribution and clinical relevance. Some continuous variables will also be transformed in categorical variables if it will be needed for description or multivariable analysis.

Categorical variables will be described as number and percentage. For key outcomes measures (hospital mortality and therapeutic withdrawals), we will also use 95% confidence interval (95% CI) to illustrate our results. For these outcomes, 95% CI will be calculated according to Wald method.

The descriptive analysis will be performed to analyse trends of patients’ characteristics, units’ characteristics and main outcome over time. These findings will be displayed year by year on two tables.

Continuous physiological variables

Relationship between primary outcome (hospital mortality) and physiological variables will be assessed. A restricted cubic spline method will be performed to test non-linearity of relationship between physiological variables and outcome. Likelihood ratio test will be used to inform about functional form of each parameter, and test linearity. If null hypothesis of linearity will be rejected, restricted cubic spline approach will be applied according to the following principles. Three, four and five knots will be applied. The Akaike Information Criterion (AIC) and Bayesian Information Criterion (BIC) will be calculated and compared to compare the fit in every cases, taking three-knots approach as reference. For each physiological parameter, the best relationship will be decided regarding fitting, plausibility and previous knowledge of the literature. In case of non-linearity, this relationship will then be used to perform multivariable analysis (for RO1 and RO2). Thus, modality of each physiological variables could potentially be changed along the analysis compare to those presented on the tables in the original SAP.

Derived score

Three scores will be used to describe the study population: APACHE 2 score and the core TBI-IMPACT score.

The APACHE 2 score is calculated in routine on the CMP according to physiological data recorded during the first 24 hours.

For the TBI-IMPACT score, we will calculate the core version of the score. This score was originally developed to predict the outcomes for TBI patients. According to the original score developed by Steyerberg et al., the core TBI-IMPACT will be calculated the following components: age, pupil reactivity and GCS motor component.

Handling of missing data

We anticipate a low rate of missing data range from 0% to 10% for the majority of variables. All primary statistical analyses will be performed on complete case data. A secondary analysis will be performed on the imputed dataset, as confirmatory analysis. Multiple imputation method will be used for every variables used in multivariable analysis (dependent and independent covariates). Multiple imputations will be performed with MICE package (R statistical software), consisting of multivariate imputation by Gibbs sampling. Five repeated multiple imputation will be performed. For other variables, no imputation method will be used.

Primary outcome

a/General principles:

An analytical logistic regression model will be performed to analyse relationship between hospital mortality and prespecified covariates. The dependant variable will be hospital mortality (Died or Alive). The main independent covariate will be the year of ICU admission, considering year 2009-2010 as reference. Other independent covariates will be those described in the ‘Methods’.

b/Univariable analysis:

The effect of each independent covariate will be estimated. Results will be displayed with unadjusted odds ratio (OR) and its 95% confidence interval (95% CI).

c/Multivariable analysis:

This analysis will include all prespecified variables regardless of the results on univariable analysis. The multivariable logistic regression model will fit the effect for all of these variables. Results will be displayed with adjusted odds ratio (OR) and 95% CI.

d/Additional covariates:

Other independent covariates, described in the ‘Method’ part, will be included in logistic model if they will be associated with hospital mortality and if they improve model fitting based on AIC value.

Secondary outcome

a/General principles:

An analytical logistic regression model will be performed to analyse relationship between therapeutic withdrawal and prespecified covariates. The dependant variable will be therapeutic withdrawal (Yes or No).

The main independent covariate will be the year of ICU admission, considering year 2009-2010 as reference. Other independent covariates will be those described in the ‘Methods’ part.

b/Univariable analysis:

Each independent covariate will be tested. Results will be displayed with unadjusted odds ratio (OR) and its 95% confidence interval (95% CI).

c/Multivariable analysis:

This analysis will include all variables associated with hospital mortality regarding the results on univariable analysis. The multivariable logistic regression model will fit the effect for all of these variables. Results will be displayed with adjusted odds ratio (OR) and 95% CI.

Other analysis

We will describe the evolution of TBI patients’ exposure to brain insults, according the same principles exposed below (cf. ‘Descriptive analysis’ part). Then, we will analyse individually each brain insult to evaluate its association with hospital mortality. This univariable analysis will follow the same principles exposed below. Finally, we will perform a multivariable analysis to evaluate their association with hospital mortality. The logistic regression model will follow the same principles exposed above. Firstly, only multivariable analysis will only include each of the brain insults as covariate. Subsequently, we will also adjust the final model on the following optional covariates: Age, surgical procedure (yes vs. no), type of ICU, pupil reactivity and GCS (motor component). As exposed for the other objectives, we will include these covariates in logistic model if they will be associated with hospital mortality and if they improve model fitting based on AIC value, without introducing collinearity. Interaction between each physiological parameters will also be tested.

Software

All analysis will be performed on R statistical software (version 4.4.1)
